# GPA: A Statistical Approach to Prioritizing GWAS Results by Integrating Pleiotropy and Annotation

**DOI:** 10.1371/journal.pgen.1004787

**Published:** 2014-11-13

**Authors:** Dongjun Chung, Can Yang, Cong Li, Joel Gelernter, Hongyu Zhao

**Affiliations:** 1 Department of Biostatistics, Yale School of Public Health, New Haven, Connecticut, United States of America; 2 Department of Public Health Sciences, Medical University of South Carolina, Charleston, South Carolina, United States of America; 3 Department of Psychiatry, Yale School of Medicine, New Haven, Connecticut, United States of America; 4 Department of Mathematics, Hong Kong Baptist University, Hong Kong, China; 5 Program in Computational Biology and Bioinformatics, Yale University, New Haven, Connecticut, United States of America; 6 VA CT Healthcare Center, West Haven, Connecticut, United States of America; 7 Department of Genetics, Yale School of Medicine, West Haven, Connecticut, United States of America; 8 Department of Neurobiology, Yale School of Medicine, New Haven, Connecticut, United States of America; 9 VA Cooperative Studies Program Coordinating Center, West Haven, Connecticut, United States of America; Stanford University, United States of America

## Abstract

Results from Genome-Wide Association Studies (GWAS) have shown that complex diseases are often affected by many genetic variants with small or moderate effects. Identifications of these risk variants remain a very challenging problem. There is a need to develop more powerful statistical methods to leverage available information to improve upon traditional approaches that focus on a single GWAS dataset without incorporating additional data. In this paper, we propose a novel statistical approach, GPA (Genetic analysis incorporating Pleiotropy and Annotation), to increase statistical power to identify risk variants through joint analysis of multiple GWAS data sets and annotation information because: (1) accumulating evidence suggests that different complex diseases share common risk bases, i.e., pleiotropy; and (2) functionally annotated variants have been consistently demonstrated to be enriched among GWAS hits. GPA can integrate multiple GWAS datasets and functional annotations to seek association signals, and it can also perform hypothesis testing to test the presence of pleiotropy and enrichment of functional annotation. Statistical inference of the model parameters and SNP ranking is achieved through an EM algorithm that can handle genome-wide markers efficiently. When we applied GPA to jointly analyze five psychiatric disorders with annotation information, not only did GPA identify many weak signals missed by the traditional single phenotype analysis, but it also revealed relationships in the genetic architecture of these disorders. Using our hypothesis testing framework, statistically significant pleiotropic effects were detected among these psychiatric disorders, and the markers annotated in the central nervous system genes and eQTLs from the Genotype-Tissue Expression (GTEx) database were significantly enriched. We also applied GPA to a bladder cancer GWAS data set with the ENCODE DNase-seq data from 125 cell lines. GPA was able to detect cell lines that are biologically more relevant to bladder cancer. The R implementation of GPA is currently available at http://dongjunchung.github.io/GPA/.

## Author Summary

In the past 10 years, many genome wide association studies (GWAS) have been conducted to identify the genetic bases of complex human traits. As of January, 2014, more than 12,000 single-nucleotide polymorphisms (SNPs) have been reported to be significantly associated with at least one complex trait/disease. On one hand, about 85% of identified risk variants are located in non-coding regions, which motivates a systematic understanding of the function of non-coding variants in regulatory elements in the human genome. On the other hand, complex diseases are often affected by many genetic variants with small or moderate effects. To address these issues, we propose a statistical approach, GPA, to integrating information from multiple GWAS datasets and functional annotation. Notably, our approach only requires marker-wise *p*-values as input, making it especially useful when only summary statistics, instead of the full genotype and phenotype data, are available. We applied GPA to analyze GWAS datasets of five psychiatric disorders and bladder cancer, where the central nervous system genes, eQTLs from the Genotype-Tissue Expression (GTEx), and the ENCODE DNase-seq data from 125 cell lines were used as functional annotation. The analysis results suggest that GPA is an effective method for integrative data analysis in the post-GWAS era.

## Introduction

Hundreds of genome-wide association studies (GWAS) have been conducted to study the genetic bases of complex human traits. As of January, 2014, more than 12,000 single-nucleotide polymorphisms (SNPs) have been reported to be significantly associated with at least one complex trait (see the web resource of GWAS catalog [1] http://www.genome.gov/gwastudies/). Despite of these successes, these significantly associated SNPs can only explain a small portion of genetic contributions to complex traits/diseases [2]. For example, human height is a highly heritable trait whose heritability is estimated to be around 80%, i.e., 80% of variation in height within the same population can be attributed to genetic effects [3]. Based on large-scale GWAS, about 180 SNPs have been reported to be significantly associated with human height [4]. However, these loci together only explain about 5-10% of variation in height [2], [4], [5]. This phenomenon is referred to as the “missing heritability” [2], [6], [7].

Identifying the source of this missing heritability has drawn much attention from researchers, and progress has been made towards explaining the apparent discrepancy. The role of a much greater-than-expected set of common variants (minor allele frequency (MAF)
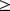
0.01) has been shown to be critical in explaining the phenotypic variance [8]. Instead of only using genome-wide significant SNPs, Yang et al. [9] reported that, by using all genotyped common SNPs, 45% of the variance for human height can be explained. This result suggests that a large proportion of the heritability is not actually missing: given the limited sample size, many individual effects of genetic markers are too weak to pass the genome-wide significance, and thus those variants remain undiscovered. So far, people have found similar genetic architectures for many other complex traits [10], such as metabolic syndrome traits [11] and psychiatric disorders [12]–[14]. That is, the phenotype is affected by many genetic variants with small or modest effects effects that cannot be confirmed individually via statistical significance, which is usually referred to as “polygenicity”. The polygenicity of complex traits is further supported by recent GWAS with larger sample sizes, in which more associated common SNPs with moderate effects have been identified (e.g., [15]). Clearly, the emerging polygenic genetic architecture imposes a great challenge of identifying risk genetic variants: a larger sample size is required to identify genetic variants with smaller effect sizes. However, sample recruitment may be expensive and time-consuming. It would be desirable to find a way to increase power to detect variants that miss significance on standard GWAS without extensive additional subject recruitment requirements. Integrative analysis of genomic data could be a promising direction, including combining GWAS data of multiple genetically related phenotypes and incorporating relevant biological information.

The last few years have seen concrete demonstrations of “pleiotropy”, i.e. the sharing of genetic factors, between human complex traits. For example, a systematic analysis of the NHGRI catalog of published GWAS (http://www.genome.gov/gwastudies/) showed that 16.9% of the reported genes and 4.6% of the reported SNPs are associated with multiple traits [16]. Through a “pleiotropic enrichment” method, Andreassen et al showed that it is possible to improve the power to detect schizophrenia-associated genetic variants by using the pleiotropy between schizophrenia (SCZ) and cardiovascular-disease [17]. A more recent study identified four significant loci (*p*-value 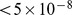) to have pleiotropic effects by analyzing GWAS data of 33,332 cases and 27,888 controls for five psychiatric disorders [18]. Further analysis suggested a very significant genetic correlation between schizophrenia and bipolar disorder (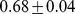 s.e.) [12]. Pleiotropy has also been demonstrated among several other types of traits, for example, metabolic syndrome traits [11] and cancers [19].

An increasing number of studies also suggest that functionally annotated SNPs are generally more biologically important than those that are not annotated, and henceforth more likely to be associated with complex traits. To name a few, using GWAS data of different traits (e.g., Crohn's disease and SCZ), Schork et al. [20] demonstrated a consistent pattern of enrichment of GWAS signals among functionally annotated SNPs. Yang et al. [21] showed that SNPs in genic regions could explain more variance of height and body mass index (BMI) than SNPs in intergenic regions. Nicolae et al. [22] found that SNPs associated with complex traits were more likely to be expression quantitative trait loci (eQTL). In addition, public availability of a vast amount of functional annotation data also provides unprecedented opportunities to investigate the enrichment of GWAS signals among these various types of functional annotations. For example, the Encyclopedia of DNA Elements (ENCODE) Consortium has recently generated extensive experimental data on gene expression (RNA-seq), DNA methylation status (RRBS-seq), chromatin modifications (ChIP-seq), chromatin accessibility (DNase-seq and FAIRE-seq), transcription factor (TF) binding sites (ChIP-seq), and long-range chromatin interactions (ChIA-PET, Hi-C, and 5C). As of September 2012, more than 1,600 data sets from 147 cell lines had been produced to annotate the human genome, including 2.89 million unique, non-overlapping DNase I hypersensitivity sites (DHSs) in 125 cell lines using DNase-seq and 630K binding regions of 119 DNA-binding proteins in 72 cell lines using ChIP-seq, among many [23]. The ENCODE Consortium [23] examined 4,492 risk-associated SNPs from the NHGRI GWAS catalog and found that 12% of them overlap with TF binding regions and 34% overlap with DHSs.

The increasing evidence of pleiotropy between complex traits and the increasing functional annotation data call for novel statistical methods to effectively analyze multiple GWAS data sets and functional annotation data simultaneously. Statistical methods to investigate pleiotropy have been actively researched (reviewed in [24] and [25]), for example, using linear mixed models [26], [27] or the conditional False Discovery Rate (FDR) approach [17], [28]. However, these methods do not allow use of functional annotation data for prioritization of GWAS results. On the other hand, various statistical methods have been proposed to make use of functionally annotated SNPs in recent years (reviewed in [29], [30], and [31]). For example, GSEA [32] identifies potentially important pathways in which target genes of risk-associated SNPs are involved while RegulomeDB [33] allows nucleotide-level annotations of risk-associated SNPs, especially for those located in non-coding regions. Stratified FDR methods have been applied to incorporate annotation into GWAS data analysis [20]. However, each of these methods was designed for the analysis of a single phenotype and hence, they do not use functional annotation data fully efficiently for the genetic variants shared by multiple phenotypes. There is a need to develop a coherent statistical framework for the integration of functional annotation data for joint analysis of genetically correlated GWAS information.

In this article, we propose a unified statistical framework, named GPA (Genetic analysis incorporating Pleiotropy and Annotation), to prioritize GWAS results based on pleiotropy and annotation information. GPA also provides statistically rigorous and biologically interpretable inference tools for this purpose. The method can easily be used for other purposes as well, as we will discuss below. This article is organized as follows. First, we investigate the properties of GPA using extensive simulation studies and illustrate the versatility and utility of GPA with the analysis of real data. Specifically, we apply GPA to the five psychiatric disorder GWAS data from the Psychiatric Genomics Consortium with central nervous system gene expression data and show that GPA can accurately identify pleiotropy structure among these diseases. We further apply GPA to the bladder cancer GWAS data with the ENCODE DNase-seq data from 125 cell lines and show that GPA can detect cell lines that are biologically more relevant to bladder cancer. Lastly, we discuss many issues related to GPA. The details of our GPA model and its statistical inference procedures are provided in the Materials and Methods section.

## Results

### Simulation study

We conducted comprehensive simulation studies to evaluate GPA performance. The *p*-values for non-risk SNPs can be simulated easily from a uniform distribution 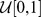. For risk-SNPs, we can simulate their *p*-values via different approaches. The most favorable simulation for our GPA model is to simulate them from the Beta distribution. To examine the robustness of our GPA model, we adopted an alternative simulation scheme under the framework of the linear mixed model and liability threshold model that has gained increasing interest recently (e.g., [9], [13]). The detailed procedures will be described later. But we emphasize that there is substantial discrepancy between the generative model used in simulation and the GPA model. The primary purpose of our simulation study is to investigate whether the GPA model can robustly improve the power to detect risk SNPs by integrating multiple GWAS data sets and annotation data despite this discrepancy.

To simulate case-control GWAS data for two genetically correlated diseases, we followed the classical liability threshold model [13]. For each disease, we first simulated a large cohort of individuals with genotypes of 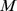 independent SNPs. The MAFs of these SNPs were drawn uniformly from [0.05, 0.5]. Then we randomly designated 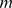 SNPs as risk SNPs. The per-minor-allele effect of each risk SNP was drawn from a normal distribution with zero-mean and variance of 
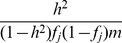
, where 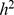 is the desired level of variance explained by all SNPs on the liability scale and 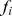 is the MAF of the corresponding risk SNP. We also simulated the environmental effect on the liability scale for each individual from a standard normal distribution (zero mean and unit variance). The total liability for each individual was then obtained by adding up all the genetic effects and the environmental effect. Given a desired disease prevalence 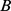, individuals with liabilities greater than the 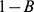 quantile were classified as cases and others were classified as controls. Then equal numbers of cases and controls were drawn from the cohort as a GWAS data set. When simulating two diseases simultaneously, we simulated two disjoint cohorts with the same set of SNPs. To reflect the pleiotropy effects between the two diseases, 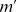 risk SNPs (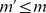) were chosen to be shared by the two diseases. The annotation status of each risk and non-risk SNP was simulated from a Bernoulli distribution with probability of 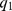 and 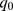, respectively.

In our simulation study, the total number of SNPs, 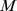, was set to be 20,000, and the sample size of each data set, 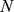, was set at 2000, 5000 or 10000, respectively. The number of risk SNPs 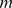 was the same for the two diseases and was set at 500, 1000 or 2000, respectively. We varied the proportion of shared risk SNPs between the two diseases, 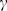, from 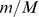 to 1. Note that 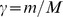 corresponds to the absence of pleiotropy. The disease prevalence, 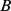, was fixed at 0.1 and the variance explained by all the SNPs, 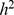, was fixed at 0.6 for each of the two diseases. Here 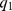 and 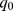 were fixed at 0.4 and 0.1, respectively.

**Figure 1:**
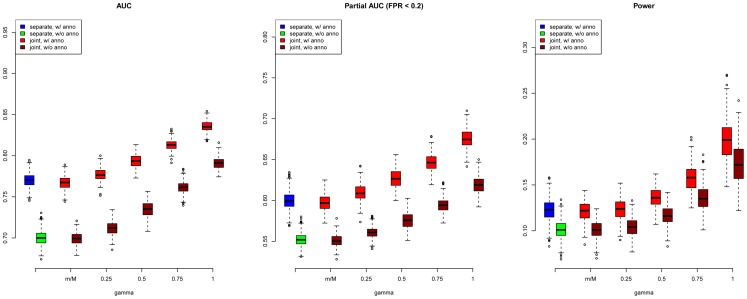
AUC (left), partial AUC (Middle) and power (right) of GPA for SNP prioritization with sample size 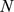 = 5000 and number of risk SNPs 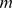 = 1000. The results are based on 200 simulations.

**Figure 2:**
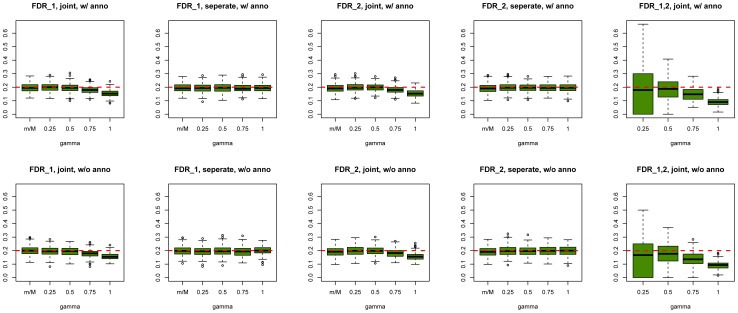
Global false discovery rates of GPA at sample size 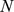 = 5000 and number of risk SNPs 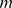 = 1000. Upper panel: Global false discovery rates of GPA with annotation. Lower panel: Global false discovery rates of GPA without annotation. From left to right: FDR of first GWAS (joint analysis), FDR of second GWAS (joint analysis), FDR of first GWAS (separate analysis), FDR of second GWAS (separate analysis) and FDR of risk variants shared by both GWAS. For all scenarios, the global false discovery rates of GPA are controlled at the nominal level.

We first evaluated SNP prioritization performance of GPA. Specifically, after the two data sets were simulated, we obtained the *p*-value for each SNP in each disease using a 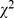 test with one degree of freedom. Then we analyzed the simulated data using our GPA method in the following four modes: 1. analyzing the two diseases separately without the annotation data; 2. analyzing the two diseases separately with the annotation data; 3. analyzing the two diseases jointly without the annotation data; and 4. analyzing the two diseases jointly with the annotation data. In each mode, we compared the order of the local FDR obtained using GPA against the actual risk status of the SNPs to calculate the area under the *receiver operating characteristic* curve (AUC) as a measure of risk SNP prioritization accuracy. The left panel of Figure 1 shows the AUCs from the four modes with 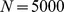 and 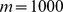 (results for other scenarios are shown in Figures S10-S20 in Text S1). Because all the simulation parameters were the same for the two diseases, only the results for the first disease are shown. We can see that incorporating either annotation information or pleiotropy between the two diseases improved the prioritization performance. In particular, as the proportion of shared risk SNPs increased, the prioritization performance also improved. Given the local FDR obtained using GPA, we controlled the global FDR at 0.2 and calculated the average power to identify the true risk SNPs. GPA performance measured by partial AUC and power for 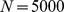 and 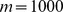 are shown in the middle and right panels of Figure 1, respectively (results for other scenarios are shown in Figures S10–S20 in Text S1). We also evaluated the actual FDR and found that the FDR was indeed controlled at 0.2 with occasional slight conservativeness (Figure 2 and Figures S21–S31 in Text S1). In addition, we evaluated the actual FDR in the presence of linkage disequilibrium (LD) among SNPs. The details of the simulations and results are given in Figure S1 in Text S1. These results suggest that GPA can improve the power of identifying risk variants while controling FDR at the nominal level despite the mismatch between GPA and the generative model in our simulation study.

Regarding parameter estimation, we found that GPA provided a satisfactory estimate of 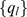, the probability of being annotated for a certain group of SNPs, as long as there are enough SNPs in that group (Figures S32–S44 in Text S1). But we note that the estimates of the proportion of risk and non-risk SNPs, 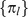, may be biased (Figures S45–S66 in Text S1). This is no surprise because the distribution of the *p*-values of the risk SNPs obtained from the generative model in the simulation study may differ from the Beta distribution assumed in GPA. If the *p*-values of the risk SNPs are indeed generated from the Beta distribution, our GPA model can give fairly accurate estimates of 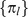 (Figures S67-S72 in Text S1).

**Figure 3:**
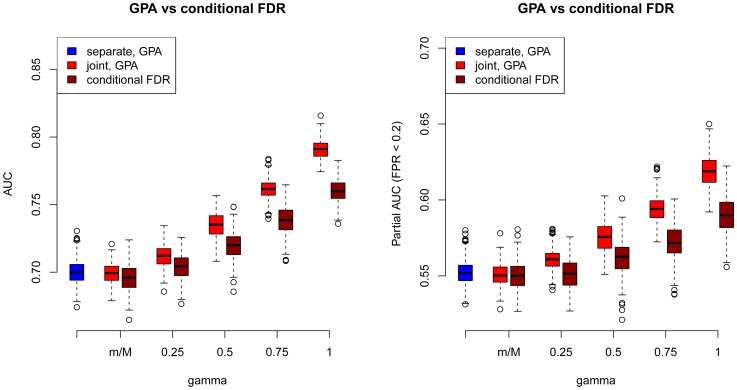
Comparisons of receiver operating characteristic curves measured by AUCs (Left) and partial AUCs (Right) between GPA and the conditional FDR approach at sample size 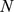 = 5000 and number of risk SNPs 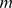 = 1000. The results are based on 200 simulations.

As a comparison, we also used the “conditional FDR” approach proposed by Andreassen et al. [17] to prioritize SNPs in our simulations. The comparison results between GPA and the conditional FDR at 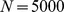 and 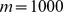 are shown in Figure 3 (other results are provided in Figures S77–S87 in Text S1). GPA significantly outperformed the conditional FDR approach in SNP prioritization. More importantly, in the absence of pleiotropy, the conditional FDR approach had worse accuracy than single-GWAS analysis using the standard FDR approach, whereas GPA achieved comparable accuracy with single-GWAS analysis in this scenario. This suggests that GPA was able to take advantage of pleiotropy when it exists while, in clear contrast to the conditional FDR approach, it does not sacrifice much statistical power than the conditional FDR approach when it is absent.

**Figure 4:**
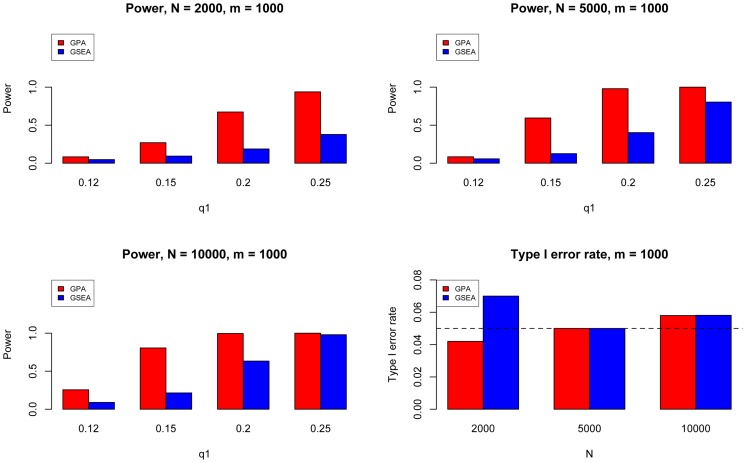
The comparison between GPA and GSEA at number of risk SNPs 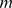 = 1000. Here we fixed 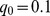 and varied 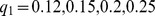 to evaluate the power for sample size 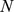 = 2000 (Upper Left panel), 5000 (Upper Right panel), 10000 (Lower Left panel), respectively. We used 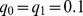 to evaluate the type I errors (Lower Right panel). The results are based on 500 simulations.

Next, we evaluated the type I error and power of GPA for hypothesis testing on the significance of annotation enrichment for risk SNPs. Gene Set Enrichment Analysis (GSEA) [34] is a popular method to accomplish a similar task. Although GSEA typically is used for gene expression data analysis, its input can be a list of *p*-values obtained from any source. Therefore we implemented the GSEA method to test the enrichment of the 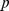-values of a set of SNPs being annotated and compared it with GPA. We followed the previous simulation scheme and simulated one GWAS data set with 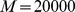, 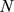 varying from 2000 to 10000, and 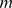 varying from 500 to 2000. Here 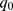 was fixed at 0.1 and 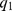 was varied from 0.1 to 0.5. We set the statistical significance level at 0.05. Type I error rate was evaluated at 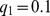 and power was evaluated at 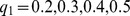. The results for 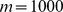 are shown in Figure 4. In general, GPA provided much higher power than GSEA while both methods appropriately controlled the type I error rate.

**Figure 5:**
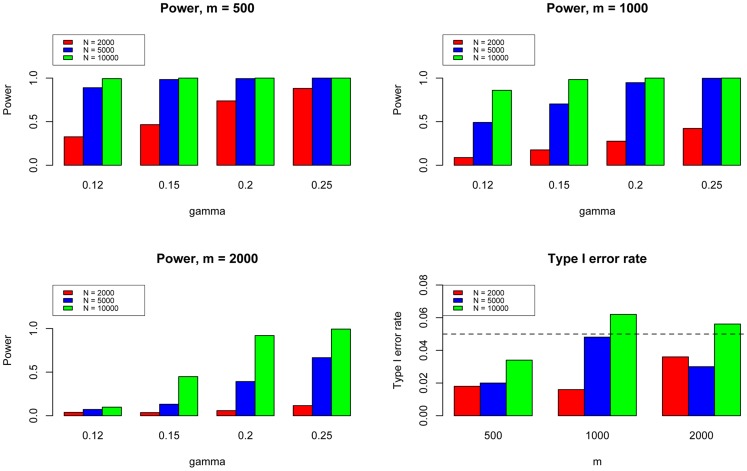
The type I error rate and power of the pleiotropy test. Here we varied 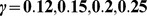 to evaluate the power for sample size 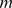 = 500 (Upper Left panel), 1000 (Upper Right panel), and 2000 (Lower Left panel), respectively. We used 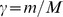 to evaluate the type I errors of the pleiotropy test (Lower Right panel). In each setting, we also varied sample size 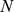 = 1000, 2000, and 10000. Note that type I error rate and power of the pleiotropy test remain almost the same in presence of annotation (see Figure S9 in Text S1).

We further evaluated the type I error rate and power of GPA for the test of pleiotropy in our simulations. The simulation parameters were the same as those in the previous simulations. Power was evaluated at 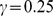, 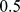, 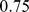, and 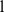. The type I error rate was evaluated at 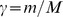, corresponding to the expected shared proportion of risk SNPs in the absence of pleiotropy. As shown in Figure 5, power increased as 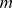 decreased and as 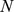 and 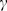 increased, whereas the type I error rate was appropriately controlled in all cases. Please note that type I errors and power remained almost the same for hypothesis testing of pleiotropy in the presence of annotation (see Figures S2–S4 in Text S1).

Lastly, we performed additional simulations with moderate heritability (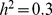) and pleiotropy (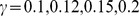). The results shown in Figures S73–S76 in Text S1 demonstrate that, with moderate heritability and pleiotropy, GPA can still effectively improve the power by leveraging pleiotropy between related traits. Therefore, GPA can serve as a more powerful tool for integrative analysis in the post-GWAS era.

### Real data analysis

#### GWAS of five psychiatric disorders

We applied our GPA model to the analysis of five psychiatric disorders, as has been done in previous publications using different methodologies [12], [18]. Traits included were attention deficit-hyperactivity disorder (ADHD), autism spectrum disorder (ASD), bipolar disorder (BPD), major depressive disorder (MDD), and schizophrenia (SCZ). Detailed information about these data sets is provided in [12], [18]. Summary statistics of the five psychiatric disorders were downloaded from the section for cross-disorder analysis at the Psychiatric Genomics Consortium (PGC) website. The *p*-values were available for 1,230,535 SNPs in ADHD, 1,245,864 SNPs in ASD, 1,233,533 SNPs in BPD, 1,232,794 SNPs in MDD, and 1,237,959 SNPs in SCZ, respectively. We took the intersection of those SNPs, resulting in a *p*-value matrix 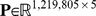 for the five psychiatric disorders.

First, we performed single-GWAS data analysis using genes preferentially expressed in the central nervous system (CNS) [13], [34] as the annotation data. Specifically, we generated the annotation vector 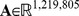 as follows: The entries in 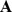 corresponding to SNPs within 50-kb of the genes from the CNS set were set to be 1. Among all the SNPs, 21.9% were thus annotated to be CNS related. The analysis results of these five psychiatric disorders are given in Table 1. The estimated fold enrichment 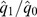 of the CNS set was 1.749 (s.e. 0.447), 1.261 (s.e. 0.055), 1.467 (s.e. 0.033), 1.177 (s.e. 0.058) and 1.391 (s.e. 0.022) for ADHD, ASD, BPD, MDD and SCZ, respectively. PGC investigators also evaluated enrichment of the CNS gene set by variance component estimation using linear mixed models (LMM) [12], suggesting about 1.6 and 1.5 fold enrichments in BPD and SCZ, respectively. Two explanations may contribute to these minor differences in fold enrichment estimation between GPA and LMM: First, GPA only used summary statistics while LMM used both phenotype and genotype data. Second, GPA and LMM employed different mathematical definitions of fold enrichment: GPA used the ratio between 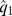 and 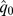, while LMM used the ratio between the proportion of the variance explained by SNPs in the CNS set and the proportion of the CNS set in entire genome. Furthermore, we evaluated the significance of enrichment of the CNS set by hypothesis testing. As shown in Table 1, enrichment of the CNS gene set was strong in BPD and SCZ, moderate in ASD and MDD, and nonsignificant in ADHD.

**Table 1:** Single-GWAS analysis of five psychiatric disorders using the CNS gene set as the annotation data.

	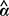	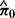	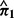	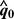	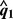	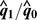	*p*-value
ADHD	0.694 (0.103)	0.991 (0.006)	0.009 (0.006)	0.218 (0.001)	0.381 (0.055)	1.749 (0.447)	0.083
ASD	0.710 (0.014)	0.909 (0.007)	0.091 (0.007)	0.214 (0.001)	0.270 (0.009)	1.261 (0.055)	8.408e-07
BPD	0.697 (0.007)	0.821 (0.007)	0.179 (0.007)	0.202 (0.001)	0.297 (0.004)	1.467 (0.033)	1.439e-48
MDD	0.837 (0.019)	0.807 (0.027)	0.193 (0.027)	0.212 (0.003)	0.249 (0.008)	1.177 (0.058)	0.005
SCZ	0.596 (0.004)	0.804 (0.004)	0.196 (0.004)	0.203 (0.001)	0.283 (0.003)	1.391 (0.022)	7.742e-79

Here 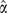 is the estimate of 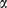 parameter of Beta distribution (4), 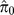 and 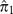 are the estimated proportion of null-SNPs and non-null-SNPs defined in (4), 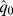 and 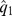 are the estimated proportion of SNPs annotated by the CNS gene set among null and non-null SNPs, respectively. Enrichment fold 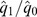 and *p*-value given by hypothesis testing of enrichment in annotation data are provided in last two columns. The values in the brackets are standard errors of the estimates.

Next, we applied GPA to study pairwise pleiotropy of these five psychiatric disorders without using annotation data. As shown in Table 2, our results suggest that the pleiotropy effect was strong between BPD and SCZ (*p*-value is essentially zero), MDD and SCZ (*p*-value 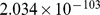), BDP and MDD (*p*-value 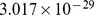), ASD and SCZ (*p*-value 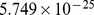), and ASD-BPD (*p*-value 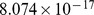); moderate between ADHD and BPD (*p*-value 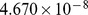), ADHD and SCZ (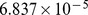); and non-significant for all other pairs. Our results largely agree with those reported in [12] and the disagreements mainly came from those between ADHD and other disorders. The pleiotropy between ADHD and MDD was reported to be moderate in [12], while GPA did not detect this moderate effect. From single GWAS analysis of ADHD, given in Table 1, the estimated parameters (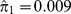
(s.e. 0.006), 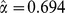) indicate that its GWAS signals were very weak. For MDD, the estimated parameter 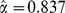 also indicates the weak marginal signals for MDD. Consequently, the marginal GWAS signals of ADHD and MDD were too weak to allow GPA to detect the pleiotropic effect between them. Since the data analysis performed in [12] used genotype data, the bivariate LMM could still have enough power to detect a moderate genetical correlation between ADHD and MDD.

**Table 2:** Pleiotropy estimated among five psychiatric disorders.

	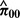	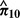	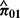	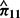	LRT	*p*-value
ADHD-ASD	0.900 (0.009)	0.007 (0.006)	0.093 (0.009)	0.001 (0.004)	0.913	0.339
ADHD-BPD	0.822 (0.008)	0.001 (0.005)	0.164 (0.009)	0.013 (0.007)	29.849	4.670e-08
ADHD-MDD	0.776 (0.036)	0.006 (0.010)	0.217 (0.036)	0.001 (0.010)	−0.005	1
ADHD-SCZ	0.804 (0.005)	0.001 (0.004)	0.183 (0.008)	0.012 (0.007)	15.855	6.837e-05
ASD-BPD	0.791 (0.008)	0.027 (0.007)	0.115 (0.009)	0.067 (0.008)	69.391	8.074e-17
ASD-MDD	0.727 (0.033)	0.049 (0.016)	0.180 (0.033)	0.044 (0.016)	2.717	0.099
ASD-SCZ	0.771 (0.006)	0.035 (0.006)	0.131 (0.007)	0.064 (0.006)	106.493	5.749e-25
BPD-MDD	0.793 (0.014)	0.011 (0.026)	0.030 (0.015)	0.166 (0.027)	126.037	3.017e-29
BPD-SCZ	0.821 (0.004)	0.001 (0.005)	0.013 (0.006)	0.165 (0.007)	1851.727	0
MDD-SCZ	0.809 (0.009)	0.001 (0.010)	0.001 (0.025)	0.189 (0.025)	466.312	2.034e-103

The values in the brackets are standard errors of the estimates. The last two columns provide the LRT statistics and *p*-values of hypothesis testing (15).

We further applied GPA to study all pairs of disorders using the CNS gene set as the annotation data. The estimated 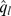 (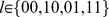) are given in Table 3 and 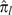 remained almost the same as those estimated without the annotation data. The *p*-values of hypothesis test (14) are provided in the last column of Table 3. The *p*-value should be interpreted with caution: as shown in Table 1, the CNS gene set is enriched in all these disorders except ADHD. Hence, the significant *p*-values listed in Table 3 may be simply due to the significant enrichments for individual traits. On the other hand, the ratio between 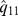 and 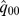 could be more interesting. Take BPD-SCZ as an example. The ratio between 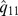 and 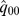 is 1.503 (s.e. 0.025), which suggests that enrichment of the CNS set for the BDP-SCZ shared risk variants was even stronger than that for BPD-only (1.467 (s.e. 0.033)) or SCZ-only (1.391 (s.e. 0.022)), although the differences are not statistically significant.

**Table 3:** GPA results for all pairs of the five psychiatric disorders with the CNS set as annotation.

	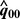	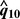	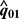	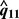	*p*-value
ADHD-ASD	0.212 (0.002)	0.425 (0.146)	0.272 (0.013)	0.022 (1.879)	4.205e-06
ADHD-BPD	0.202 (0.003)	0.975 (0.261)	0.304 (0.010)	0.216 (0.143)	2.965e-47
ADHD-MDD	0.209 (0.004)	0.490 (0.411)	0.251 (0.014)	0.001 (2.357)	0.005
ADHD-SCZ	0.204 (0.002)	0.001 (2.349)	0.261 (0.015)	0.511 (0.100)	7.132e-79
ASD-BPD	0.204 (0.003)	0.164 (0.073)	0.285 (0.015)	0.318 (0.022)	2.058e-51
ASD-MDD	0.193 (0.006)	0.470 (0.069)	0.309 (0.018)	0.002 (0.171)	3.251e-09
ASD-SCZ	0.199 (0.002)	0.295 (0.028)	0.296 (0.008)	0.255 (0.018)	2.078e-81
BPD-MDD	0.195 (0.004)	0.568 (0.025)	0.367 (0.066)	0.222 (0.023)	7.314e-48
BPD-SCZ	0.206 (0.001)	0.001 (6.907)	0.026 (0.720)	0.309 (0.006)	2.860e-130
MDD-SCZ	0.204 (0.002)	0.001 (16.882)	0.732 (1.019)	0.260 (0.011)	7.324e-78

The estimated 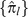 almost remain the same as Table 2 and 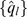 are shown in the table. The values in the brackets are standard errors of the estimates. The *p*-values of hypothesis testing (14) are provided in the last column.

**Figure 6:**
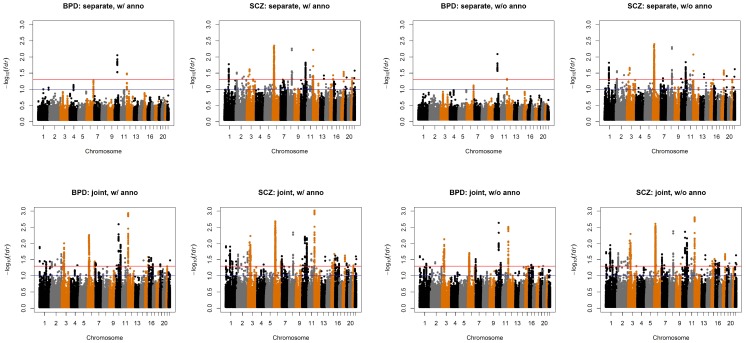
Manhattan plots of BPD and SCZ. Top left panel: separate analysis without annotation. Top right panel: separate analysis with CNS annotation. Bottom left panel: joint analysis without annotation. Bottom right panel: joint analysis with CNS annotation. The red and blue lines indicate local 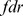 = 0.05 and 0.1, respectively.

**Figure 7:**
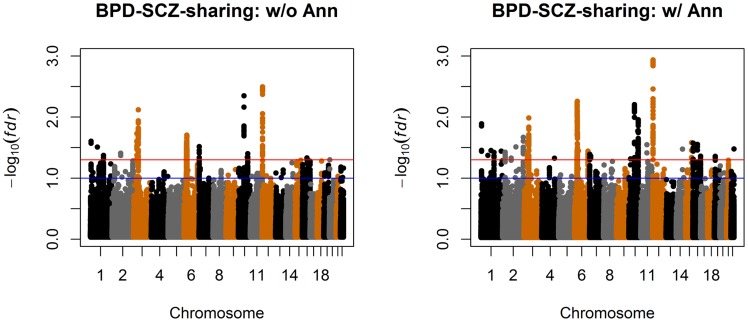
Manhattan plots of local false discovery rates 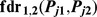 and 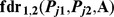 (Equations (11) and (12)) for detecting BPD-SCZ-sharing SNPs. Left panel: joint analysis without annotation. Right panel: joint analysis with annotation. The red and blue lines indicate local 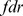 = 0.05 and 0.1, respectively.

We also compared the results given by four different analysis approaches: single-GWAS analysis with or without annotation, and two-GWAS joint analysis with or without annotation data. The Manhattan plots are shown in Figures 6 and 7. For single-GWAS analysis without annotation, GPA identified 13 SNPs and 391 SNPs with local false discovery rate 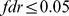 for BPD and SCZ, respectively. By using the CNS set as annotation, GPA was able to identify 14 and 409 SNPs for BPD and SCZ, respectively, with the same fdr control. For joint analysis without annotation, the number of identified SNPs increased to 383 and 821 for BPD and SCZ, respectively. By using the CNS set as annotation, the number of identified SNPs further increased to 385 and 837 for BPD and SCZ, respectively. We investigated the BPD results in detail to evaluate the power of GPA in identification of functionally important SNPs. For single-GWAS analysis of BPD, GPA was able to identify SNPs located in the *ANK3* gene. By using annotation data, the *CACNA1C* gene, which encodes an alpha-1 subunit of a voltage-dependent calcium channel, was identified by GPA. After incorporating pleiotropy information between SCZ and BPD, additional functionally relevant genes, such as *PBRM1*, *C6orf136*, *DPCR1*, *SYNE1*, were identified by GPA. For instance, *SYNE1* encodes the synaptic nuclear envelope protein 1, and codes the protein Syne-1 that is found in many tissues and is especially critical in the brain. The Syne-1 protein is active (expressed) in Purkinje cells, which are located in the cerebellum and are involved in signaling between neurons. Mutations in the *SYNE1* gene have been found to cause autosomal recessive cerebellar ataxia type 1 (ARCA1) and *SYNE1* has recently been implicated as a susceptibility gene for BPD in a large collaborative GWAS study [35]. Clearly, the present results indicate that the statistical power to identify associated SNPs increased by making use of pleiotropy and functional annotation (in this real data example, pleiotropy played a more important role than functional annotation).

We also applied GPA with multiple annotation datasets to improve its performance. Beside the CNS gene set, we considered eQTL annotation from GTEx [36] and transcription factor binding site (TFBS) annotation by ANNOVAR [37]. Specifically, we downloaded the available eQTL data from the GTEx website (http://www.ncbi.nlm.nih.gov/gtex/GTEX2/gtex.cgi) and took the intersection of these eQTL with the markers of the psychiatric disorders, resulting in 23,505 SNPs annotated as eQTL. To obtain our TFBS annotation, we used the key word “TFBS” to query the ANNOVAR database, resulting in 19,029 SNPs annotated as TFBS (More details can be found at http://www.openbioinformatics.org/annovar/annovar_faq.html#tfbs). We performed joint analysis of BPD and SCZ with these three annotations (CNS, eQTL and TFBS). The estimated parameters in the GPA model and their standard errors are shown in Table 4. Notice that 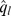 hit the boundary of the parameter space in presence of the eQTL annotation. This made the EM algorithm converge in fewer iterations, resulting in the minor differences between the estimated parameters 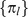 in Table 4 and those in Table 2. With the local FDR 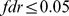, GPA identified 724 and 977 risk SNPs for BPD and SCZ, respectively. Clearly, the enrichment of eQTL is high with fold 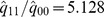 (s.e. 0.152) and the enrichment of TFBS is moderate with 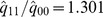 (s.e. 0.063). These results demonstrate that GPA is able to incorporate multiple annotation datasets for prioritization of GWAS results with good effects.

**Table 4:** The estimated parameters and their standard errors for the joint analysis of BPD and SCZ, together with multiple annotation data: The CNS gene set, eQTL and TFBS.

BPD-SCZ	00	10	01	11
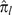	0.808 (0.004)	0.025 (0.188)	0.033 (0.189)	0.134 (0.005)
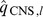	0.211 (0.001)	0.001 (5.911)	0.001 (4.386)	0.359 (0.006)
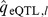	0.013 ( 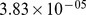 )	0.001 (0.125)	0.001 (0.092)	0.066 (3.23 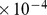 )
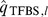	0.016 ( 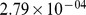 )	0.001 (0.110)	0.001 (0.068)	0.021 (0.001)

The first row (“00”, “10”, “01” and “11”) corresponds to the 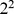 combinatorial states.

Besides the evaluation of the statistical power of GPA on real data, we examined whether the Beta distribution of the GPA model fit the real data. We compared the *p*-values of real data and the *p*-values simulated from the fitted GPA model to examine the goodness of fit. The results are given in Figures S88–S97 in Text S1, suggesting that our GPA model fitted the real data well.

Regarding computational time, the GPA algorithm takes less than 20 minutes to analyze two traits with one annotation data file. The speed of convergence depends on the strength of the GWAS signals. For example, it took about 7 mins and 3 mins to analyze ADHD and SCZ, repectively, as SCZ has stronger GWAS signals than ADHD. For joint analysis of BPD and SCZ, it took about 20 mins. All timings were carried out on a desktop with 3.0 GHz CPU and 16G memory.

### Bladder cancer GWAS and ENCODE annotation data

DNase I hypersensitive sites (DHSs) are regions where DNA degradation by enzymes like DNase I occur more frequently than elsewhere. As a result, DHSs can mark active transcription regions across genome and these patterns are known to be tissue or cell specific. The ENCODE project analyzed the DHSs in 125 human cell lines with the intention of cataloging human regulatory DNA [38]. In this section, we applied GPA to assess how bladder cancer [39] risk associated SNPs are enriched in DHSs region across these 125 human cell lines.

We downloaded genotype data for bladder cancer from dbGaP (NCI Cancer Genetic Markers of Susceptibility (CGEMS) project; accession number phs000346.v1.p1). We used samples genotyped from both Illumina 1 M chip and 610K chip for our analysis. For quality control, we removed SNPs with missing rates 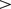
0.01. We checked Hardy-Weinberg Equilibrium and excluded SNPs with *p*-value 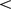
0.001. SNPs with minor allele frequencies (MAF) 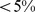 were also removed. After quality control, 490,614 SNPs from 3,631 cases and 3,356 controls of European descent were used in the analysis. SNP-level association p-values were calculated for this bladder cancer data using logistic regression by assuming an additive genetic model. We also downloaded the uniform peak files for DHSs in 125 cell lines from the ENCODE project (http://genome.ucsc.edu/cgi-bin/hgFileUi?db=hg19&g=wgEncodeAwgDnaseUniform). Note that the DHSs for these 125 cell lines were identified with a uniform analysis workflow by the ENCODE Consortium; this facilitates fair and unbiased comparison among cell lines as annotation for our GPA model.

**Figure 8:**
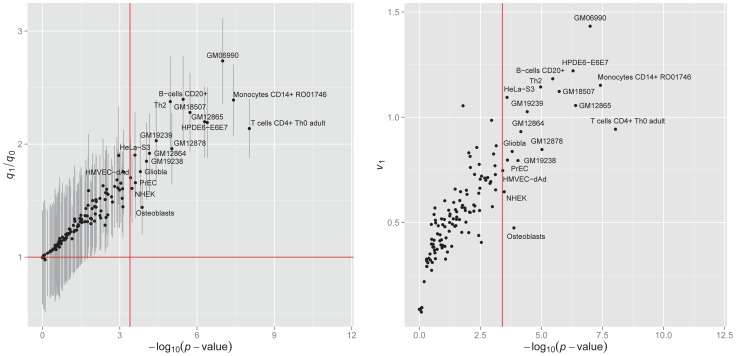
Enrichment of the DNase I hypersenstivity site annotation data from 125 cell lines for bladder cancer. Left panel: 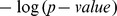 of hypothesis testing (13) vs. fold enrichment 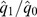. The vertical red line corresponds to the significance level (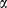 = 0.05) after Bonferroni correction. The horizontal red line corresponds to ratio = 1. Right panel: The normalized variance component 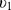 (2) given by LMM v.s. 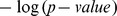 given by GPA.

We applied GPA to analyze the bladder cancer GWAS 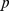-values with one annotation dataset at a time, and performed hypothesis testing to assess the significance of enrichment. The results are shown in the left panel of Figure 8. Under significance level 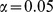 after Bonferroni correction, annotations from 19 cell lines were statistically significantly enriched for bladder cancer risk associated SNPs. Most of these cell lines were derived from lymphocytes from normal blood (e.g., T cells CD4+ Th0 adult, Monocytes CD14+ RO01746), while some cell lines came from cancer patients (e.g., Gliobla and HeLa-S3). The above results suggest that involvement of the immune system and carcinoma pathways in bladder cancer. These results also demonstrate that GPA may be an effective way to explore functional roles of GWAS hits by testing enrichment on phenotype-related annotations or user-specified annotations.

We also compared GPA with the LMM-based approach [21], [40] for this dataset. Specifically, we considered the following genome-partitioning LMM: 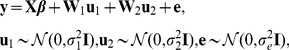
(1)where 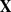 are covariates (the first five principal components from genotype data), and 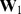 and 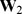 are sets of SNPs overlapping DHSs in each cell line and the remaining SNPs, respectively. We denote the numbers of SNPs in 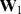 and 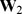 as 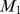 and 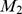, respectively. The median number of SNPs that overlap DHS in each cell line is about 60K and 90% of cell lines have the number of DHSs ranging between 40K and 80K. In order to take into account such variation in DHS number among cell lines, we define a scaled version of the proportion of phenotype variance explained by SNPs overlapping DHSs in each cell line as 
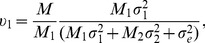
(2)where 
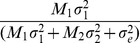
 is the proportion of the explained variance and 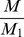 is the scaling factor. The right panel of Figure 8 shows that the (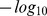)-transformed *p*-value of the GPA annotation enrichment test is linearly related to 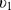. This indicates that our GPA model captures enrichment of annotation almost as accurately as LMM even without the original genotype data, implying its broader applicability than methods requiring individual genotype and phenotype data.

## Discussion

Many GWAS have been conducted in the past 10 years that have led to the discoveries of thousands of genomic regions associated with many traits, and many more discoveries are expected from ongoing GWAS. As GWAS data accumulate, there is an urgent need to perform a systematic analysis of available GWAS datasets for a comprehensive understanding of the genetic architecture of complex traits, and provide new insights for functional roles of the implicated variants. To achieve this goal, there is a great interest in developing computational and statistical approaches to exploring genomic data in the post-GWAS era.

In the following, we briefly discuss the relationship between GPA and other related methods, such as LMM, conditional FDR and GSEA. LMM is an effective method for exploring genetic architecture of complex traits and it has been implemented in a popular software package, GCTA [41]. Compared with LMM, GPA has the following distinctive features:

- LMM explores the genetic architecture underlying complex traits/diseases by estimating the gross phenotypic variance that can be explained by whole genome or a certain subset of SNPs. In contrast, GPA provides more “fine-grained” analysis by giving estimates of the local false discovery rate of each SNP, the proportion of SNPs that are associated with the phenotype (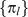), the overall effect strength of the associated SNPs (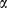), and enrichment of a particular functional annotation (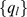). GPA also provides the standard errors to measure the uncertainty of those estimates.
- Application of LMM requires the availability of genotype data, while GPA only needs the summary statistics (*p*-values) as its input. Typically, the genotype data may not be accessible as easily as the summary statistics. For example, when researchers want to implement integrative analysis for their own GWAS data at hand with related GWAS studies, it is much easier for them to obtain the summary statistics than the whole data sets of related GWAS studies. In this sense, GPA may greatly simplify the procedure of integrative analysis.

To our best knowledge, the conditional FDR approach is the first approach that statistically addresses the issue of pleiotropy between two GWAS. and GSEA is presently the most popular approach to evaluating the enrichment of gene sets. In fact, GPA provides a unified framework for systematically integrating both sources of information, pleiotropy and annotation. Rigorous statistical inference of pleiotropic effects and annotation enrichment has been established in this framework. As demonstrated in our extensive simulation study, GPA has better performance of identifying disease-associated markers than the conditional FDR approach, and it shows greater power of evaluating annotation enrichment than GSEA as well. With real data analysis, we have demonstrated how to use GPA to incorporate pleiotropy information and multiple annotation data for prioritizing GWAS results.

Here we briefly discuss some key assumptions made in GPA.

- GPA assumes that the *p*-values from null SNPs and non-null SNPs follow the uniform distribution and the Beta distribution, respectively. Although GPA performs robustly when *p*-values are obtained from the random-effects model, as shown via simulation study, preparation of valid *p*-values as GPA input is critical for its successful application. For example, if population stratification and cryptic relatedness have not been accounted for in GWAS data analysis, the obtained *p*-value of null SNPs will not follow the uniform distribution, resulting in inflated type I errors of GPA. Therefore, these confounding effects in GWAS data analysis should be correctly accounted for before applying GPA. Principal component analysis based approaches [42] or linear mixed models [43]–[45] can be used to address these issues effectively.
- We assumed independence between SNPs in our model. This independence assumption greatly simplified our model and led to very efficient computation, making GPA useful in practice. We also evaluated the LD effects on our GPA model using simulation. As expected, a risk SNP can propagate its effect to SNPs in LD with itself. In this case, the type I error of GPA depends on the definition of a “true” risk SNP. If we regard the SNPs identified in the flanking region of the risk SNP as true positives, then the type I error of GPA can be controlled at the nominal level. On the other hand, we compared performance between GPA and the commonly accepted random-effects model for enrichment analysis for bladder-cancer GWAS data, where GPA provided consistent results with the commonly accepted random-effects model.
- We implicitly assumed the proportion of risk SNPs should not be extremely small to enable GPA to work well. We performed simulations to investigate GPA performance when the proportion of risk SNPs was extremely small (Figures S5–S8 in Text S1). The simulation results suggest that the proportion can be poorly estimated when the true 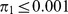. However, the type I error of GPA can be still controlled at the nominal level even in this case. Regarding real data analysis, we have observed that 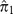 can range from 0.009 to 0.196 with relatively small standard errors (Table 1).
- We assumed the conditional independence of multiple annotation datasets (see GPA model (9)). In the presence of multiple highly correlated annotations, the current version of GPA may not perform well. Hence, when multiple sources of annotation data are available, the correlation among the annotation datasets should be evaluated before using GPA with them. In fact, in the analysis of psychiatric disorders, the correlation among the three annotation data (CNS, eQTL and TFBS) is nearly zero, and thus we can analyze them simultaneously. If they are highly correlated, we suggest incorporating them into GPA one at a time, as shown in our analysis of bladder cancer GWAS data with ENCODE annotation. We realize that simultaneous analysis of multiple correlated annotation data may be more powerful and will investigate this issue in our future work.

The parameters in the GPA model should be interpreted with caution because parameter estimation is based on the model assumption as discussed above. For example, as we showed in simulation study, the estimated 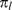 can be biased due to the mismatch of GPA model and the random-effects model.

There are also some limitations of the current GPA model. Although extensions to three or more GWAS are straightforward in principle (from four-groups model to 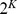-groups model, 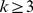), the number of groups will increase exponentially as the number of GWAS increases. This makes many 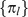 close to zero, resulting in poor parameter estimation (large variance) and thus reduced power. Currently, we suggest doing pairwise-GWAS analysis to explore the genetic relationship of different phenotypes. It is of great interest to develop statistical models to handle the multiple-GWAS case more effectively. Another limitation is that current GPA version can only handle binary annotation. Allowing more annotation structures (e.g., continuous annotation) in GPA is an important extension of current GPA model. We will investigate this issue in the future.

In summary, we have presented a statistical approach, named GPA, that can integrate information from multiple GWAS data sets and functional annotation data. Not only does GPA have better statistical power than related methods, it also provides interpretable model parameters offering insights to our understanding of the genetic architecture of complex traits. We have successfully applied GPA to analyze GWAS data of five psychiatric disorders from PGC, and showed that GPA is able to identify pleiotropic effects among psychiatric disorders and detect enrichment of the CNS gene set. We have also applied GPA to analyze a bladder cancer GWAS dataset with ENCODE data as annotation, where significant enrichments of immune system and carcinoma pathways were observed. Compared to LMM that requires individual genotype and phenotype data as input, GPA has similar results of enrichment analysis without requirements of the genotype data. This makes GPA an attractive and effective tool for the integrative analysis of multiple GWAS data with functional annotation data, when genotype data are not available.

## Materials and Methods

### GPA probabilistic model

Throughout this paper, we shall use 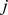 to index SNPs, 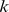 to index GWAS data sets, and 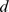 to index the annotation data sets. We first consider the simplest case where we only have summary statistics (*p*-values) from just one GWAS data set, and then extend our model to handle multiple GWAS data sets and annotation data. Suppose we have performed hypothesis testing of genome-wide SNPs and obtained their *p*-values: 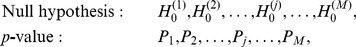
(3)where 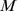 is the number of SNPs. Consider the “two-groups model” [46], i.e., the obtained *p*-values are assumed to come from the mixture of null and non-null, with probability 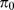 and 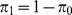, respectively. Let 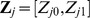 be the latent variables indicating whether the *j*-th SNP is null or non-null, where 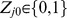, 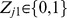, and 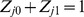, because a SNP can only be either null or non-null. Here 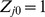 means un-associated (null) and 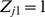 means associated (non-null). Then we have the following two-groups model: 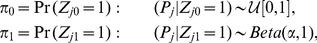
(4)where the *p*-values from the null group are from the Uniform distribution on [0,1], denoted as 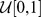, and the *p*-values from the non-null group are from the Beta distribution with parameters (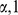), where 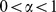. We put the constraint 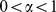 to model that a smaller *p*-value is more likely than a larger *p*-value when it is from the non-null group [47].

To incorporate information from functional annotation data, we extend the basic model as follows. Suppose we have collected information from 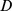 functional annotation sources in the annotation matrix: 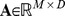, where 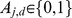 indicates whether the *j*-th SNP is annotated in the *d*-th functional annotation source. For example, when there are two annotation sources – eQTL data and DNase I hypersensitivity sites (DHS) data – then 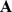 is an 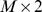 matrix. If the *j*-th SNP is an eQTL, then 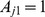, otherwise 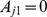; if it is located in a DHS, then 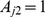, otherwise 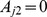. Now we model the relationship between 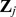 and 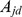 as 
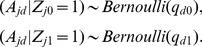
(5)Clearly, 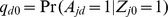 can be interpreted as the proportion of null SNPs being annotated in the *d*-th annotation, and 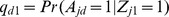 corresponds to the proportion of non-null SNPs being annotated in the *d*-th annotation. Therefore, 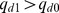 implies that there exists enrichment for the *d*-th annotation. The statistical inference about enrichment of annotation data will be discussed in details in Section “Hypothesis testing of annotation enrichment and pleiotropy”.

Now we extend the above model to handle multiple GWAS data sets. To keep the notation uncluttered, we present the model for the case of two GWAS data sets. Suppose we have *p*-values from two GWAS: 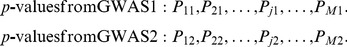
(6)Let 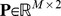 be the matrix collecting all the *p*-values, where 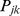 denotes the *p*-value of the *j*-th SNP in the *k*-th GWAS. Similarly, we introduce latent variables 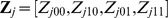 indicating the association between the *j*-th SNP and the two phenotypes: 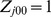 means the *j*-th SNP is associated with neither of them, 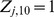 means it is only associated with the first one, 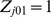 means it is only associated with the second one, and 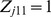 means it is associated with both. The two-groups model (4) is extended to the following “four-groups model”: 
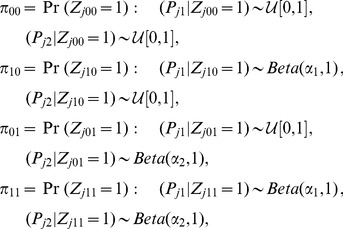
(7)where 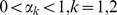. When the genetic bases of the two phenotypes are independent of each other (i.e., no pleiotropy), then we have 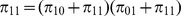 by expectation. Therefore, the difference between 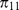 and 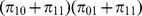 can be used to characterize pleiotropy. Statistical inference on pleiotropy is given in Section “Hypothesis testing of annotation enrichment and pleiotropy”.

To incorporate annotation information into the multiple GWAS model (7), similarly, we model the relationship between 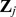 and 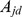 as 
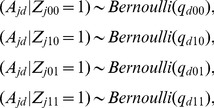
(8)where 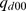 is the probability of a null SNP being annotated, 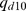 is the probability of the first phenotype associated-SNP being annotated, 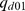 is the probability of the second phenotype associated-SNP being annotated, and 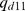 is the probability of jointly associated-SNP being annotated. Assuming the independence of SNP markers, the joint distribution 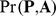 can be written as 
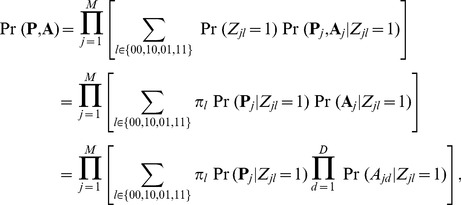
(9)where 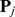 and 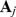 are the *j*-th row of 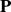 and 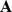; the second equation holds by assuming the independence between 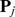 and 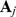, conditional on 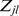; and the third equation holds by further assuming the independence between 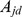 and 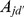 for 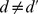, conditional on 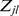.

Parameters in the GPA model can be estimated using the Expectation-Maximization (EM) algorithm [48], which is computationally efficient because we have explicit solutions for estimation of all the parameters in the M-step. Standard errors for parameter estimates can be approximated using the empirical observed information matrix [49]. Note that in the GPA model, the sample size for estimating the empirical observed information matrix corresponds to the number of SNPs and as a result, we have a very large sample size (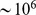) to estimate standard errors accurately. More details of the EM algorithm and the estimation of standard errors are provided in Sections 1 and 3 in Text S1.

### Statistical inference

#### False discovery rate

After we estimate the parameters in the GPA model, SNPs can be prioritized based on their local false discovery rates [50]. Note that separate analysis of single GWAS data based on their summary statistics is equivalent to the analysis of single GWAS data using GPA without any annotation data. Hence, separate analysis of single GWAS data can be considered as a special case of our GPA approach.

To present the local false discovery rate based on our GPA model, we begin with the simplest case: single GWAS without annotation data. In this case, there are only two groups: null and non-null. The false discovery rate is defined as the probability that the *j*-th SNP belongs to the null group given its *p*-value, i.e., 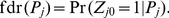
(10)For joint analysis of two GWAS data sets, we are interested in the local false discovery rate of the *j*-th SNP, if it is claimed to be associated with the first phenotype, the second one, and both, i.e., 
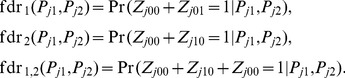
(11)Similarly, when annotation data are available, the false discovery rates can be calculated as 
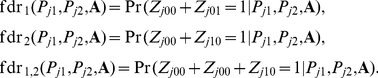
(12)Then, we use the *direct posterior probability approach* [51] to control global false discovery rates. More details for the estimation of false discovery rates are provided in Section 2 in Text S1.

#### Hypothesis testing of annotation enrichment and pleiotropy

Given an annotation file, we may be interested in whether there is statistical evidence for enrichment of GWAS signals in this annotation file. We propose to use the likelihood ratio test (LRT) to assess the statistical significance. To keep the notation simple, we drop the index of annotation files. Specifically, the significance of enrichment of an annotation for single-GWAS data analysis can be assessed by the following test: 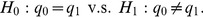
(13)The LRT statistic is: 
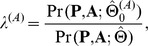
where 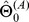 collects the parameter estimates obtained under 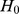, and the superscript *A* indicates the Annotation enrichment test. Note that 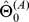 can be easily obtained by running the GPA algorithm without incorporating the annotation data. Under the null, the test statistic 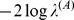 asymptotically follows the 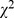 distribution with 1 degree of freedom [52]. We reject 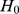 if 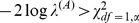, where 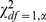 is the 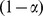-th quantile of 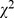 distribution with 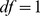.

For joint analysis of two GWAS with annotation data, test (13) becomes 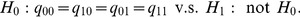
(14)Under the null, the test statistic asymptotically follows a 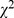 distribution with 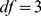. Similarly, the test of annotation enrichment can be extended to handle 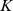 GWAS. In this case, the test statistic asymptotically follows 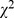 distribution with 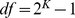 under the null.

Now we consider testing pleiotropy between two GWAS. When there is no pleiotropy, i.e., the signals from the two GWAS are independent of each other, testing pleiotropy can be formulated by testing the following hypothesis: 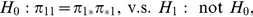
(15)where 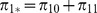 and 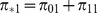. The LRT statistic is constructed as follows: 
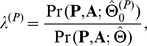
where 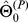 represents the parameter estimates obtained under 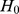, and the superscript *P* indicates the Pleiotropy test. The test statistic (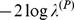) asymptotically follows 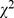 distribution with 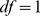 under the null.

## Supporting Information

Text S1 Supporting information for GPA.
(PDF)
