# Supplementary material for: GPA: A Statistical Approach to Prioritizing GWAS Results by Integrating Pleiotropy and Annotation
Source: PLoS Genet. 2014 Nov 13;10(11):e1004787. doi: 10.1371/journal.pgen.1004787 (PMC4230845; doi:10.1371/journal.pgen.1004787)
Supplement: Text S1 — Supporting information for GPA. (PDF) [file pgen.1004787.s001.pdf]

Text S1: Supporting information for GPA

Dongjun Chung[1,2]*, Can Yang[1,3,4]*, Cong Li[5],
Joel Gelernter[3,6,7,8] and Hongyu Zhao[1,5,7,9]†

[1]Department of Biostatistics, Yale School of Public Health,
New Haven, Connecticut, USA.

2 Department of Public Health Sciences,
Medical University of South Carolina, USA.

[3]Department of Psychiatry, Yale School of Medicine,
New Haven, Connecticut, USA.

[4]Department of Mathematics,
Hong Kong Baptist University, Hong Kong.

[5]Program in Computational Biology and Bioinformatics, Yale University,
New Haven, Connecticut, USA.

[6]VA CT Healthcare Center, West Haven, Connecticut, USA.

[7]Department of Genetics, Yale School of Medicine,
West Haven, Connecticut, USA.

[8]Department of Neurobiology, Yale School of Medicine,
New Haven, Connecticut, USA.

[9]VA Cooperative Studies Program Coordinating Center,
West Haven, Connecticut, USA.

September 4, 2014

1 The Expectation-Maximization algorithm

In order to be consistent with the main text, we present the EM algorithm for the case that we have two GWAS data but the generalization to more than two GWAS data is straightforward and the actual GPA algorithm is not limited to the number of GWAS data. In the GPA model, we have the parameter vector of length $(2^K + K + 2^K D)$ as

$$\Theta = (\pi_{00}, \pi_{10}, \pi_{01}, \pi_{11}, \alpha_1, \alpha_2, [q_{d00}, q_{d10}, q_{d01}, q_{d11}]_{d \in \{1, \cdots, D\}})$$

*These authors contributed equally to this work.
†Corresponding author

and the complete likelihood as

$$L_c(\Theta) = \prod_{j=1}^{M} \prod_{l \in \{00,10,01,11\}} \left[\pi_l \Pr(P_{j1}, P_{j2} | Z_{jl} = 1; \Theta) \prod_{d=1}^{D} \Pr(A_{jd} | Z_{jl} = 1; \Theta) \right]^{Z_{jl}}.$$

If annotation data are not incorporated, this complete likelihood is simplified to

$$L_c(\Theta) = \prod_{j=1}^{M} \prod_{l \in \{00,10,01,11\}} \left[\pi_l \Pr(P_{j1}, P_{j2} | Z_{jl} = 1; \Theta) \right]^{Z_{jl}}.$$

Based on this complete likelihood, the E- and M-steps in the t-th iteration of the EM algorithm are as follows.

E-step:

For $l \in \{00, 10, 01, 11\}$, the posterior probabilities for association of the j-th SNP are obtained as:

$$\begin{aligned} z_{jl}^{(t)} &= \Pr(Z_{jl} = 1 | \mathbf{P}, \mathbf{A}; \Theta^{(t)}) \\ &= \frac{\pi_l^{(t)} \Pr(P_{j1}, P_{j2} | Z_{jl} = 1; \Theta^{(t)}) \prod_{d=1}^{D} \Pr(A_{jd} | Z_{jl} = 1; \Theta^{(t)})}{\sum_{l' \in \{00,10,01,11\}} \pi_{l'}^{(t)} \Pr(P_{j1}, P_{j2} | Z_{jl'} = 1; \Theta^{(t)}) \prod_{d=1}^{D} \Pr(A_{jd} | Z_{jl'} = 1; \Theta^{(t)})}. \end{aligned}$$

If annotation data are not incorporated, for $l \in \{00, 10, 01, 11\}$, we have

$$z_{jl}^{(t)} = \Pr(Z_{jl} = 1 | \mathbf{P}; \Theta^{(t)}) = \frac{\pi_l^{(t)} \Pr(P_{j1}, P_{j2} | Z_{jl} = 1; \Theta^{(t)})}{\sum_{l' \in \{00,10,01,11\}} \pi_{l'}^{(t)} \Pr(P_{j1}, P_{j2} | Z_{jl'} = 1; \Theta^{(t)})}.$$

M-step:

The parameters for the proportion of SNPs in each association status category are estimated as:

$$\pi_{00}^{(t+1)} = \frac{1}{M} \sum_{j=1}^{M} z_{j00}^{(t)}, \pi_{10}^{(t+1)} = \frac{1}{M} \sum_{j=1}^{M} z_{j10}^{(t)}, \pi_{01}^{(t+1)} = \frac{1}{M} \sum_{j=1}^{M} z_{j01}^{(t)}, \pi_{11}^{(t+1)} = \frac{1}{M} \sum_{j=1}^{M} z_{j11}^{(t)}.$$

The parameters for the enrichment of the d-th annotation data for association of SNPs are estimated as:

$$q_{d00}^{(t+1)} = \frac{\sum_{j=1}^{M} z_{j00}^{(t)} A_{jd}}{\sum_{j=1}^{M} z_{j00}^{(t)}}, q_{d10}^{(t+1)} = \frac{\sum_{j=1}^{M} z_{j10}^{(t)} A_{jd}}{\sum_{j=1}^{M} z_{j10}^{(t)}}, q_{d01}^{(t+1)} = \frac{\sum_{j=1}^{M} z_{j01}^{(t)} A_{jd}}{\sum_{j=1}^{M} z_{j01}^{(t)}}, q_{d11}^{(t+1)} = \frac{\sum_{j=1}^{M} z_{j11}^{(t)} A_{jd}}{\sum_{j=1}^{M} z_{j11}^{(t)}}.$$

The parameters for the signal strength of the GWAS data are estimated as:

$$\alpha_1^{(t+1)} = \frac{\sum_{j=1}^{M} (z_{j10}^{(t)} + z_{j11}^{(t)})}{\sum_{j=1}^{M} (z_{j10}^{(t)} + z_{j11}^{(t)})(-log P_{j1})}, \alpha_2^{(t+1)} = \frac{\sum_{j=1}^{M} (z_{j01}^{(t)} + z_{j11}^{(t)})}{\sum_{j=1}^{M} (z_{j01}^{(t)} + z_{j11}^{(t)})(-log P_{j2})}.$$

The M step remains the same when annotation data are not incorporated, except that we do not need to calculate $q_{d00}^{(t+1)}$, $q_{d10}^{(t+1)}$, $q_{d01}^{(t+1)}$, and $q_{d11}^{(t+1)}$.

2 Estimation of false discovery rate

For analysis of single GWAS without annotation data, the local false discovery rate can be calculated as

$$\mathrm{fdr}(P_j) = \hat{\Pr}(Z_{j0} = 1|P_j) = \frac{\hat{\pi}_0 \Pr(P_j|Z_{j0} = 1; \hat{\Theta})}{\hat{\pi}_0 \Pr(P_j|Z_{j0} = 1; \hat{\Theta}) + \hat{\pi}_1 \Pr(P_j|Z_{j1} = 1; \hat{\Theta})}, \tag{1}$$

where $\hat{\pi}_0$, $\hat{\pi}_1$, and $\hat{\Theta}$ are estimated from the EM algorithm.

For joint analysis of two GWAS data sets, we are interested in the local false discovery rate of the j-th SNP, if it is claimed to be associated with the first phenotype and the second one, i.e.,

$$\begin{aligned}
\mathrm{fdr}_1(P_{j1}, P_{j2}) &= \hat{\Pr}(Z_{j00} + Z_{j01} = 1|P_{j1}, P_{j2}) = \frac{\Pr(P_{j1}, P_{j2}, Z_{j00} + Z_{j01} = 1; \hat{\Theta})}{\Pr(P_{j1}, P_{j2}; \hat{\Theta})}, \\
\mathrm{fdr}_2(P_{j1}, P_{j2}) &= \hat{\Pr}(Z_{j00} + Z_{j10} = 1|P_{j1}, P_{j2}) = \frac{\Pr(P_{j1}, P_{j2}, Z_{j00} + Z_{j10} = 1; \hat{\Theta})}{\Pr(P_{j1}, P_{j2}; \hat{\Theta})},
\end{aligned} \tag{2}$$

where

$$\begin{aligned}
\Pr(P_{j1}, P_{j2}; \hat{\Theta}) &= \sum_{l \in \{00,10,01,11\}} \hat{\pi}_l \Pr(P_{j1}, P_{j2}|Z_{jl}; \hat{\Theta}), \\
\Pr(P_{j1}, P_{j2}, Z_{j00} + Z_{j01} = 1; \hat{\Theta}) &= \sum_{l \in \{00,01\}} \hat{\pi}_l \Pr(P_{j1}, P_{j2}|Z_{jl}; \hat{\Theta}), \\
\Pr(P_{j1}, P_{j2}, Z_{j00} + Z_{j10} = 1; \hat{\Theta}) &= \sum_{l \in \{00,10\}} \hat{\pi}_l \Pr(P_{j1}, P_{j2}|Z_{jl}; \hat{\Theta}),
\end{aligned} \tag{3}$$

and $\{\hat{\pi}_l\}_{l \in \{00,10,01,11\}}$ and $\hat{\Theta}$ are estimated parameters from the GPA model.

When annotation data are available, the false discovery rates can be calculated as

$$\begin{aligned}
\mathrm{fdr}_1(P_{j1}, P_{j2}, \mathbf{A}; \hat{\Theta}) &= \hat{\Pr}(Z_{j00} + Z_{j01} = 1|P_{j1}, P_{j2}, \mathbf{A}) = \frac{\Pr(P_{j1}, P_{j2}, \mathbf{A}, Z_{j00} + Z_{j01} = 1; \hat{\Theta})}{\Pr(P_{j1}, P_{j2}, \mathbf{A}; \hat{\Theta})}, \\
\mathrm{fdr}_2(P_{j1}, P_{j2}, \mathbf{A}; \hat{\Theta}) &= \hat{\Pr}(Z_{j00} + Z_{j10} = 1|P_{j1}, P_{j2}, \mathbf{A}) = \frac{\Pr(P_{j1}, P_{j2}, \mathbf{A}, Z_{j00} + Z_{j10} = 1; \hat{\Theta})}{\Pr(P_{j1}, P_{j2}, \mathbf{A}; \hat{\Theta})},
\end{aligned} \tag{4}$$

where

$$\begin{aligned}
\Pr(P_{j1}, P_{j2}, \mathbf{A}; \hat{\Theta}) &= \sum_{l \in \{00,10,01,11\}} \left(\hat{\pi}_l \Pr(P_{j1}, P_{j2}|Z_{jl}; \hat{\Theta}) \prod_{d=1}^{D} \Pr(A_{jd}|Z_{jl}); \hat{\Theta} \right), \\
\Pr(P_{j1}, P_{j2}, \mathbf{A}, Z_{j00} + Z_{j01} = 1; \hat{\Theta}) &= \sum_{l \in \{00,01\}} \left(\hat{\pi}_l \Pr(P_{j1}, P_{j2}|Z_{jl}; \hat{\Theta}) \prod_{d=1}^{D} \Pr(A_{jd}|Z_{jl}; \hat{\Theta}) \right), \\
\Pr(P_{j1}, P_{j2}, \mathbf{A}, Z_{j00} + Z_{j10} = 1; \hat{\Theta}) &= \sum_{l \in \{00,10\}} \left(\hat{\pi}_l \Pr(P_{j1}, P_{j2}|Z_{jl}; \hat{\Theta}) \prod_{d=1}^{D} \Pr(A_{jd}|Z_{jl}; \hat{\Theta}) \right).
\end{aligned} \tag{5}$$

Finally, we use the *direct posterior probability approach* [3] to control global false discovery rates to determine associated SNPs. Specifically, given the GPA model fitting, we first sort SNPs by their local false discovery rates from the smallest one to the largest one. Denote local false discovery rates of these sorted SNPs by f_j. We increase the threshold for local false discovery rates, κ, from zero to one until

$$Fdr = \frac{\sum_{j=1}^{J} f_j 1\{f_j \leq \kappa\}}{\sum_{j=1}^{J} 1\{f_j \leq \kappa\}} \leq \tau, \tag{6}$$

where τ is the pre-determined bound of global false discovery rates. Finally, we determine SNPs with corresponding $f_j < \kappa$ to be associated with the phenotype.

3 Estimation of standard error

To understand the genetic architecture of complex traits, we are interested in the accuracy of the estimated parameters ($\{\pi_l\}, \{q_{d,l}\}$) from the GPA model. Here we consider the standard errors of those parameters which can be calculated from covariance matrix estimated using the empirical observed information matrix [2]. Specifically, the empirical observed information matrix is defined as

$$I_e(\hat{\Theta}; \mathbf{P}, \mathbf{A}) = \sum_{j=1}^{M} s_j(\mathbf{P}_j, \mathbf{A}_j; \hat{\Theta}) s_j^T(\mathbf{P}_j, \mathbf{A}_j; \hat{\Theta}),$$

where $\hat{\Theta}$ are the parameter estimates from the EM algorithm (i.e., maximum likelihood estimator (MLE)),

$$s_j(\mathbf{P}_j, \mathbf{A}_j; \Theta) = E_\Theta \left\{ \frac{\partial \log L_{cj}(\Theta)}{\partial \Theta} | \mathbf{P}, \mathbf{A} \right\},$$

and $\log L_{cj}(\Theta)$ is the complete log likelihood for the j-th SNP. $s_j(\mathbf{P}_j, \mathbf{A}_j; \Theta)$ is a column vector of length $(2^K - 1 + K + 2^K D)$. The first three $(2^K - 1)$ components of s_j correspond to $\pi_{10}, \pi_{01}, \pi_{11}$:

$$s_j^{(1)}(\mathbf{P}_j, \mathbf{A}_j; \hat{\Theta}) = \frac{\hat{z}_{j10}}{\hat{\pi}_{10}} - \frac{\hat{z}_{j00}}{\hat{\pi}_{00}}, s_j^{(2)}(\mathbf{P}_j, \mathbf{A}_j; \hat{\Theta}) = \frac{\hat{z}_{j01}}{\hat{\pi}_{01}} - \frac{\hat{z}_{j00}}{\hat{\pi}_{00}}, s_j^{(3)}(\mathbf{P}_j, \mathbf{A}_j; \hat{\Theta}) = \frac{\hat{z}_{j11}}{\hat{\pi}_{11}} - \frac{\hat{z}_{j00}}{\hat{\pi}_{00}},$$

where $\hat{z}_{jl} = P\left(Z_{jl} = 1 | \mathbf{P}, \mathbf{A}, \hat{\Theta}\right), l \in \{00, 10, 01, 11\}$. The next two (K) components of s_j correspond to α_1, α_2:

$$s_j^{(4)}(\mathbf{P}_j, \mathbf{A}_j; \hat{\Theta}) = (\hat{z}_{j10} + \hat{z}_{j11}) \{\log P_{j1} + 1/\hat{\alpha}_1\},$$
$$s_j^{(5)}(\mathbf{P}_j, \mathbf{A}_j; \hat{\Theta}) = (\hat{z}_{j01} + \hat{z}_{j11}) \{\log P_{j2} + 1/\hat{\alpha}_2\}.$$

The remaining $(2^K D)$ components of s_j correspond to $q_{d00}, q_{d10}, q_{d01}, q_{d11}, d = 1, \cdots, D$:

$$s_j^{(6)}(\mathbf{P}_j, \mathbf{A}_j; \hat{\Theta}) = (\hat{z}_{j00})(\frac{A_{jd}}{\hat{q}_{d00}} - \frac{1 - A_{jd}}{1 - \hat{q}_{d00}}), s_j^{(7)}(\mathbf{P}_j, \mathbf{A}_j; \hat{\Theta}) = (\hat{z}_{j10})(\frac{A_{jd}}{\hat{q}_{d10}} - \frac{1 - A_{jd}}{1 - \hat{q}_{d10}}),$$
$$s_j^{(8)}(\mathbf{P}_j, \mathbf{A}_j; \hat{\Theta}) = (\hat{z}_{j01})(\frac{A_{jd}}{\hat{q}_{d01}} - \frac{1 - A_{jd}}{1 - \hat{q}_{d01}}), s_j^{(9)}(\mathbf{P}_j, \mathbf{A}_j; \hat{\Theta}) = (\hat{z}_{j11})(\frac{A_{jd}}{\hat{q}_{d11}} - \frac{1 - A_{jd}}{1 - \hat{q}_{d11}}).$$

The last part is simply ignored when annotation data is not incorporated.

We do not include a component corresponding to π_{00} in $s_j(\mathbf{P}_j, \mathbf{A}_j; \Theta)$ because of the relationship that $\pi_{00} = 1 - \pi_{10} - \pi_{01} - \pi_{11}$. Instead, after we estimate the empirical observed information matrix, we estimate the standard error for π_{00} using the Delta method [5] as

$$se(\hat{\pi}_{00}) = \sqrt{\{g\prime(\Theta)\}^T \left\{ I_e(\hat{\Theta}; \mathbf{P}, \mathbf{A}) \right\}^{-1} \{g\prime(\Theta)\}},$$

where $g(\Theta) = 1 - \pi_{10} - \pi_{01} - \pi_{11}$, $g\prime(\Theta) = [-1, -1, -1, \mathbf{0}]$, and $\mathbf{0}$ is the zero vector of length $(K + 2^K D)$.

4 Simulation study for evaluating the LD effects on GPA

To study the LD effects on our GPA model, we used the observed genotype data from 3,356 control samples in the bladder cancer GWAS data set. In our simulation, we partitioned these samples into two groups of equal size to mimic the two-GWAS case, and only used 37,352 SNPs in chromosome 1.

We considered the case that the risk SNPs were more or less uniformly distributed along the chromosome. We simulated a risk SNP every 1000 SNPs. As a result, we had 37 risk SNPs potentially shared between the two GWAS. Typically, about 5% phenotypic variance can be explained by a chromosome, but sometime this value can go up to about 15% (e.g., Yang J. et al Genome partitioning of genetic variation for complex traits using common SNPs, Nature Genetics, 2011). To simulate different signal strengths, we adopted the random-effects model and set heritability $h^2 = 5\%, 10\%, 20\%$ jointly contributed by the 37 risk SNPs.

In the presence of LD effects, it would be difficult to distinguish the casual signals from the associated signals due to LD statistically. It is expected that only the local genomic region in LD with the identified risk genetic variants can be pinpointed through GWAS analysis. Here we used different distance thresholds to define the flanking region of a risk variant. We only regarded the identified SNPs outside the flanking region of the true risk SNPs as false positives.

We evaluated GPA performance for different h^2 and γ, where γ is the proportion of shared risk SNPs between the two GWAS. We also considered three cases for annotation: without annotation, with useful annotation (SNPs within 1Mb of risk SNPs are annotated), and with random annotation (i.e., SNPs are annotated randomly). All the results are summarized based on 100 simulations, as shown in Figure S1. First, the observed false positive rate (FDR) decreased as the distance threshold increased, which suggests that the false positives happen in the local regions of risk SNPs. Second, the observed FDR was higher for larger h^2. This is because a risk SNP of a larger effect size can propagate its effect to SNPs in weaker LD with itself. In summary, we believe that, for the complex diseases considered by us, where the individual chromosomal contribution may be between 5% and the shared pleiotropy is around 50% of less, GPA can provide a satisfactory FDR control in term of pinpointing a local genomic region associated with the phenotype of interest.

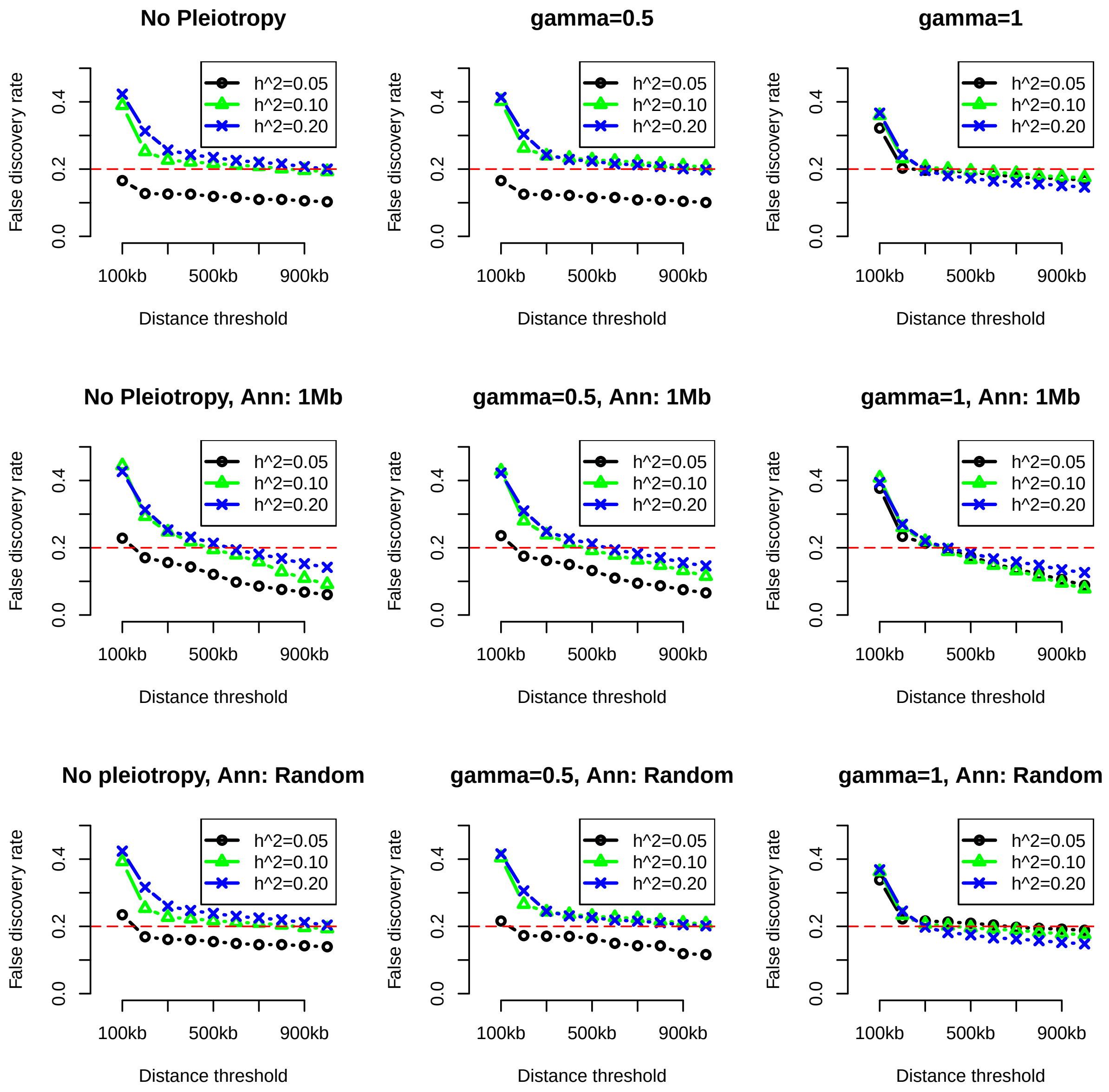

Figure S1: Upper Panel: Results without annotation. Middle Panel: Results with annotation (SNPs within 1Mb of risk SNPs are annotated). Lower Panel: Results with random annotation (SNPs are annotated randomly). Here γ is the proportion of shared risk SNPs between the two GWAS. We reported the GPA results by controlling global false discovery rate at 0.2, as indicated by the dashed red line. In each subplot, the observed false discovery rates at different signal strength levels ($h^2 = 5\%, 10\%, 20\%$) are shown based on different distance thresholds for the flanking region.

5 Simulation study for evaluating noise annotation on GPA

To evaluate GPA performance when irrelevant annotation is used, we performed some additional simulation studies. He we also used the random-effects model as described in the main article. We varied the number of annotations from 1 to 4. As shown in Figures S2-S4, incorporation

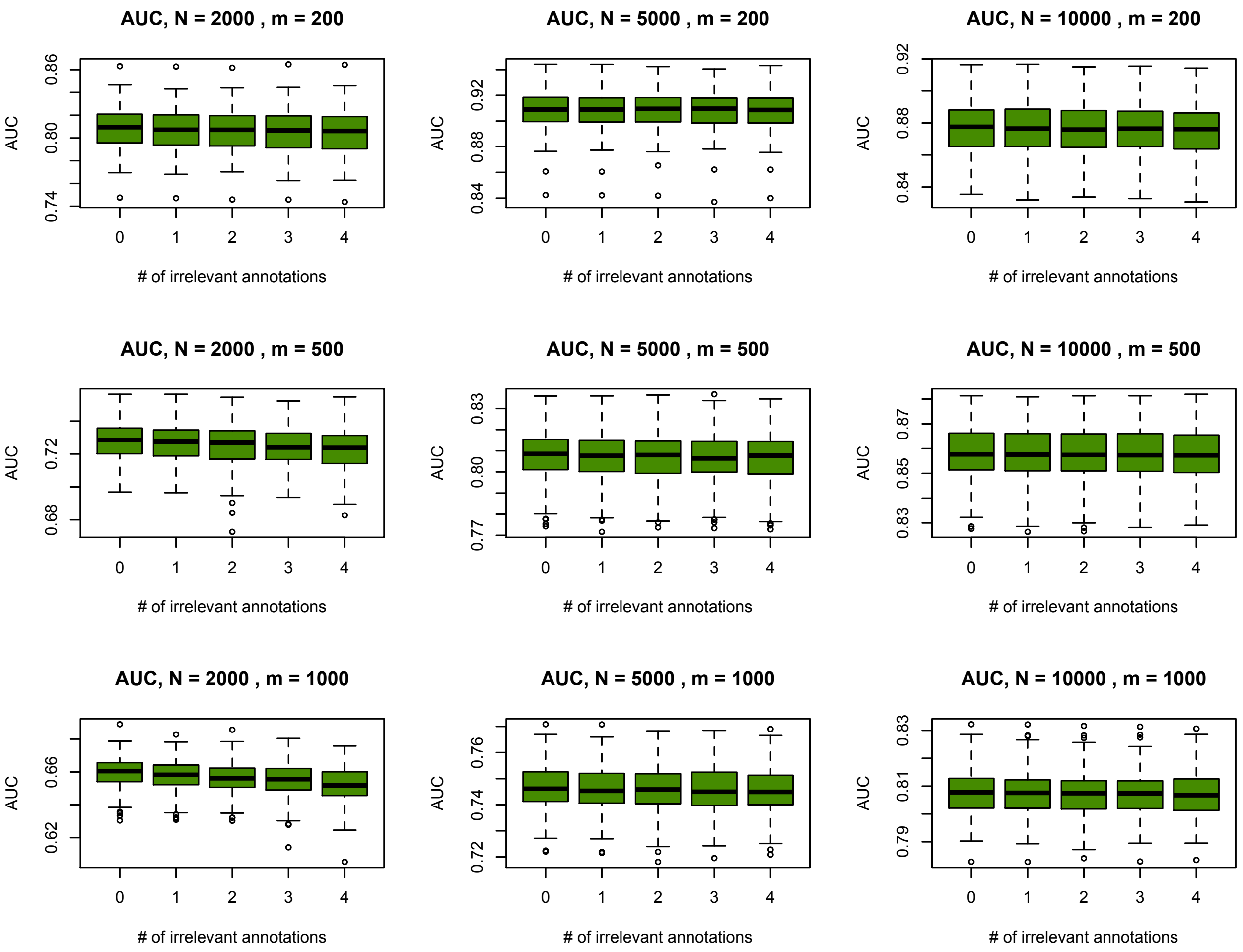

Figure S2: GPA performance measured by AUC when a few irrelevant annotations are incorporated. Here $q_1 = q_0 = 0.1$, N and m are the sample size and the number of risk SNPs, respectively.

of irrelevant annotations do not lead to reduced power because q_0 and q_1 can be estimated accurately.

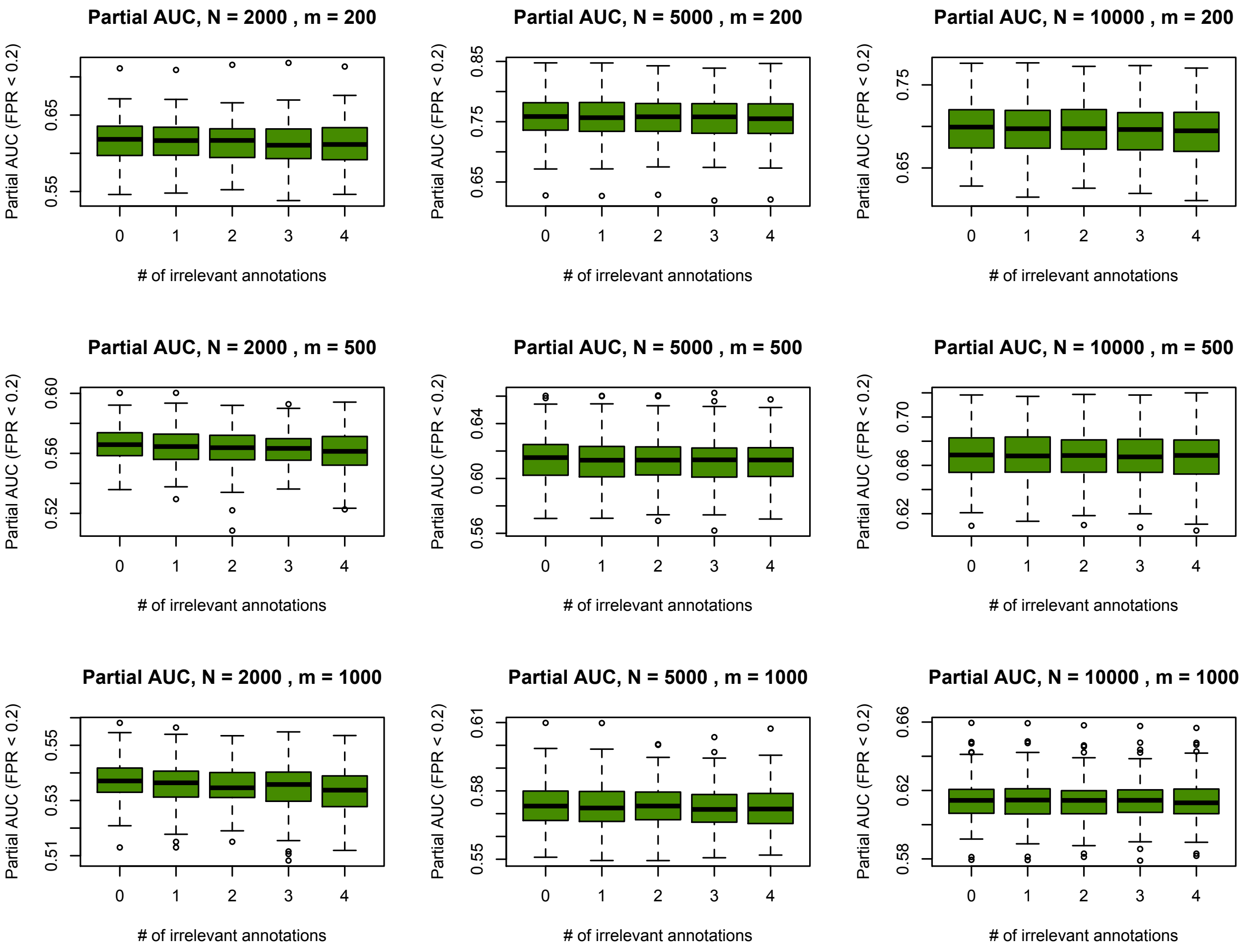

Figure S3: GPA performance measured by partial AUC when a few irrelevant annotations are incorporated. Here $q_1 = q_0 = 0.1$, N and m are the sample size and the number of risk SNPs, respectively.

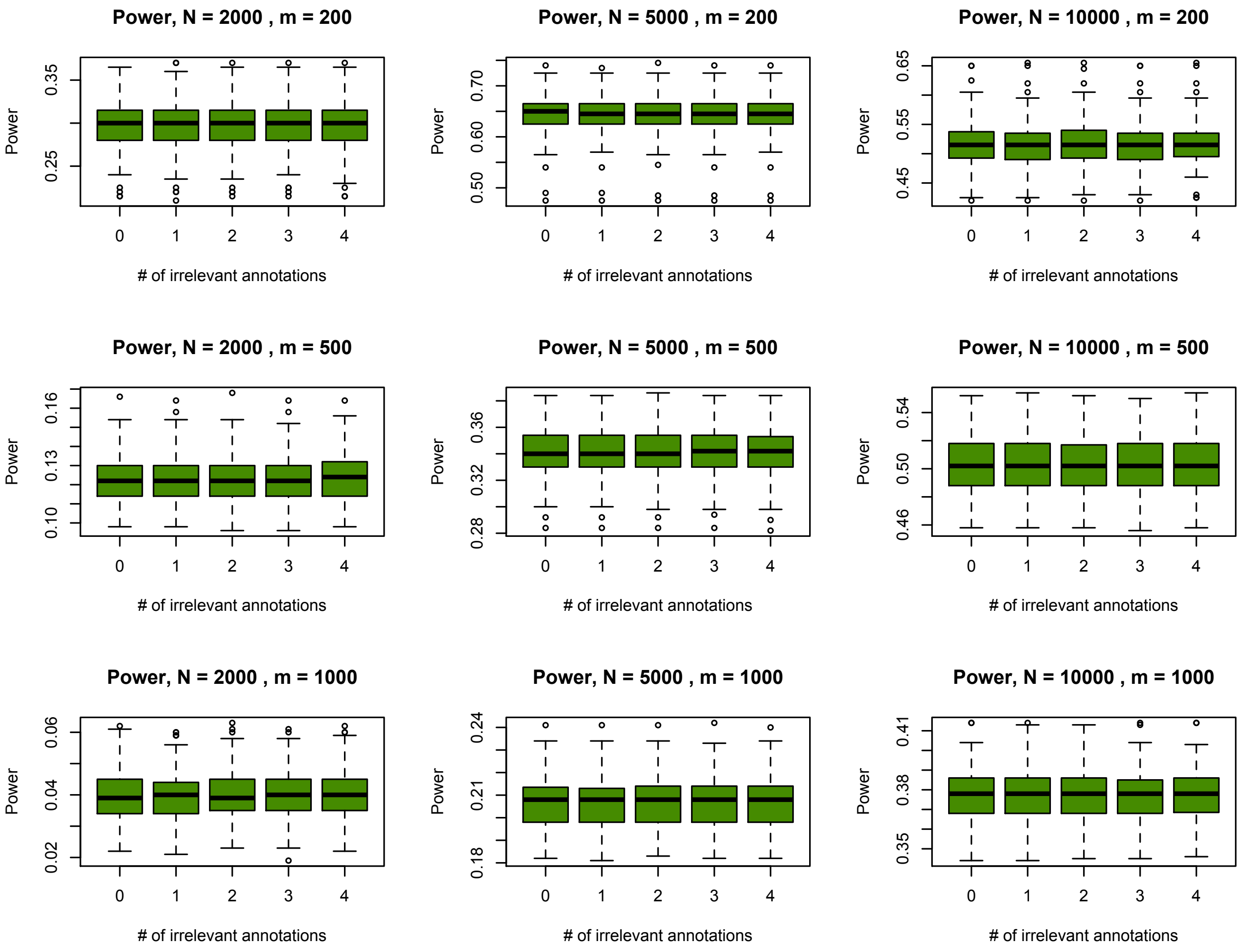

Figure S4: GPA performance measured by power when a few irrelevant annotations are incorporated. Here $q_1 = q_0 = 0.1$, N and m are the sample size and the number of risk SNPs, respectively.

6 Simulation study when the proportion of risk SNPs should not be extremely small

We implicitly assumed the proportion of risk SNPs should not be extremely small to enable GPA work well. This assumption is made based on the polygenic genetic architecture of complex traits. We used simulation to investigate GPA performance when the proportion of risk SNPs was extremely small. In the main article, we used the liability model to evaluate whether our GPA model could work well in presence of model mismatch (The Beta distribution used in the GPA model is different from the distribution of the p-values from the liability model). To avoid the effect of model mismatch, here we used the GPA model to check whether our EM algorithm could give accurate estimates when the proportion of non-null SNPs is small, in which we only needed to specify $\{\pi_l\}$ and the α parameter in the Beta distribution. For convenience, we first provide more details for single-GWAS case and then discuss about the two-GWAS case. The results of parameter estimation and FDR control for the single-GWAS case are shown in Figure S5, where α was fixed at 0.6 (corresponding to a moderate GWAS signal). We can see that, although the proportion of risk SNPs may not be estimated accurately if the true proportion of risk SNPs is extremely small ($\pi_1 \leq 0.001$), GPA can provide a valid FDR control.

Notice that the power of GPA is low when π_1 is smaller. This is because the α parameter was fixed at 0.6. The effect size of each risk SNP remained the same while π_1 became smaller, resulting in weaker and weaker signals. However, if we adopted the liability model with *fixed* heritability h^2, smaller π_1 will imply a larger individual effect size of a risk SNP. Thus, the power of GPA can be higher. To examine this, we used the liability model (as the same as the main article) for simulation studies. The performance of GPA at different heritability h^2 is shown in Figure S6. We did observe that the power was even higher when π_1 was smaller.

We also conducted a simulation study checking the effect of lower bounds of π in the GPA algorithm when some of $\{\pi_l\}$ are small in the joint analysis of two GWAS data. Specifically, we considered the joint analysis of two GWAS data with $(\pi_{00}, \pi_{01}, \pi_{10}, \pi_{11})$ = (0.8999, 0.0001, 0.0001, 0.0999) and generated the p-values from the mixture of the uniform distribution and the Beta distribution (The α values in the two Beta distributions were fixed at 0.6). We evaluated the GPA model fitting results for different lower bounds of the π parameters, 10^{-3}, 10^{-4}, 10^{-5} and 10^{-6}. As shown in Figure S7, the true value was between the first and third quartiles of each of the parameter estimates and the estimates were only weakly affected by the lower bounds of π parameters. More importantly, all the false discovery rates for the first phenotype (fdr_1), the second phenotype (fdr_2), and both phenotypes ($\mathrm{fdr}_{1,2}$), were well controlled at a nominal level, regardless of the lower bound for π parameters (Figure S8), where fdr_1, fdr_2 and $\mathrm{fdr}_{1,2}$ were defined as the same as in the main article. In summary, the GPA algorithm works robustly even when some of proportions are small and it is affected only weakly by the lower bound for π parameters.

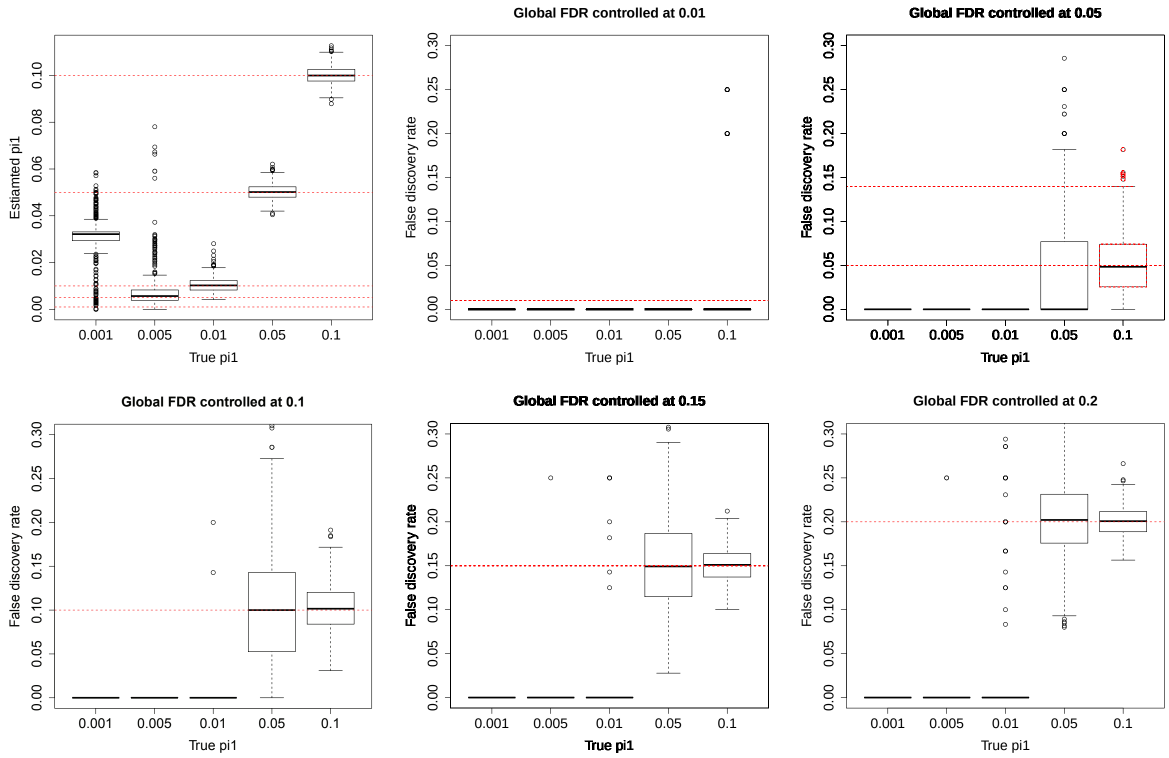

Figure S5: Simulation study for the single-GWAS case. Upper panel: the subfigures from left to right are parameter estimation ($\hat{\pi}_1$ v.s. true π_1), the GPA results for controlling FDR at 0.01, the GPA results for controlling FDR at 0.05. Lower panel: the subfigures from left to right are the GPA results for controlling FDR at 0.1, the GPA results for controlling FDR at 0.15 and the GPA results for controlling FDR at 0.2. All the results are summarized based on 100 simulations, where the α parameter in the Beta distribution was fixed at 0.6.

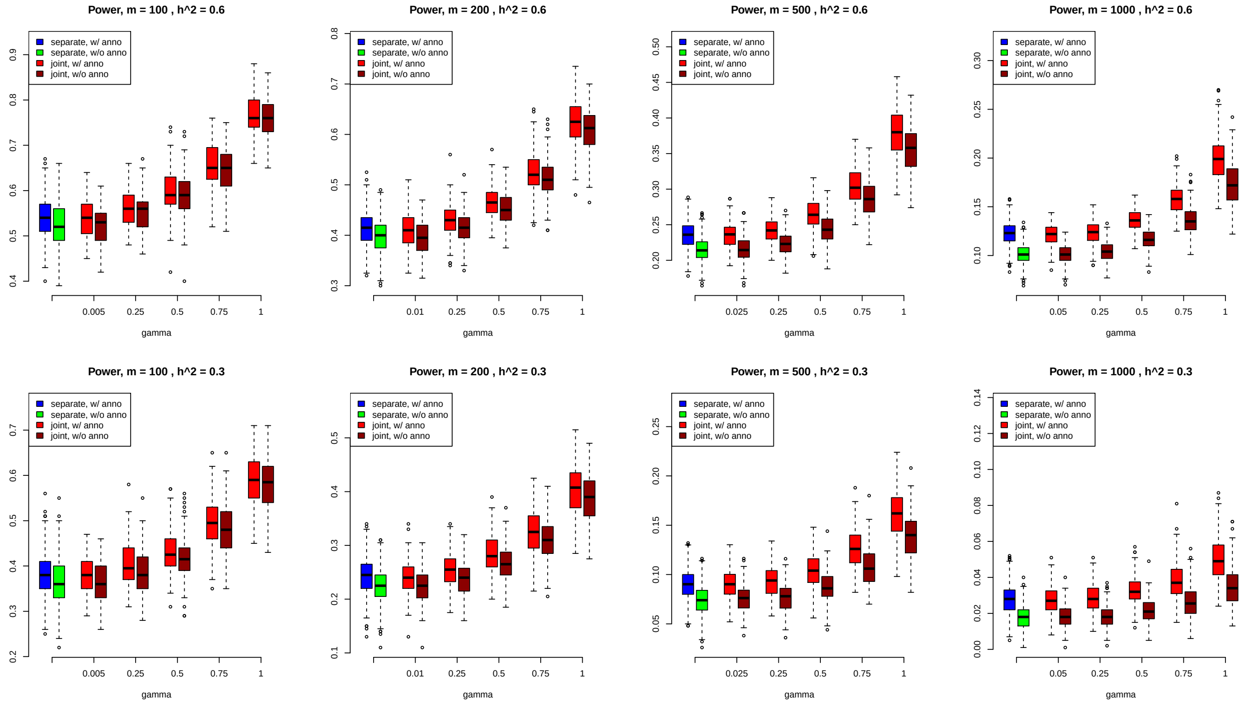

Figure S6: The power of GPA at different π values (sample size $N = 5000$). Here $m = 100, 200, 500, 1000$ corresponds to $\pi = 0.005, 0.01, 0.02, 0.05$ respectively. Upper Panel: High heritability scenario ($h^2 = 0.6$). Lower Panel: Moderate heritability scenario ($h^2 = 0.3$). Here γ is the proportion of shared risk SNPs between the two GWAS.

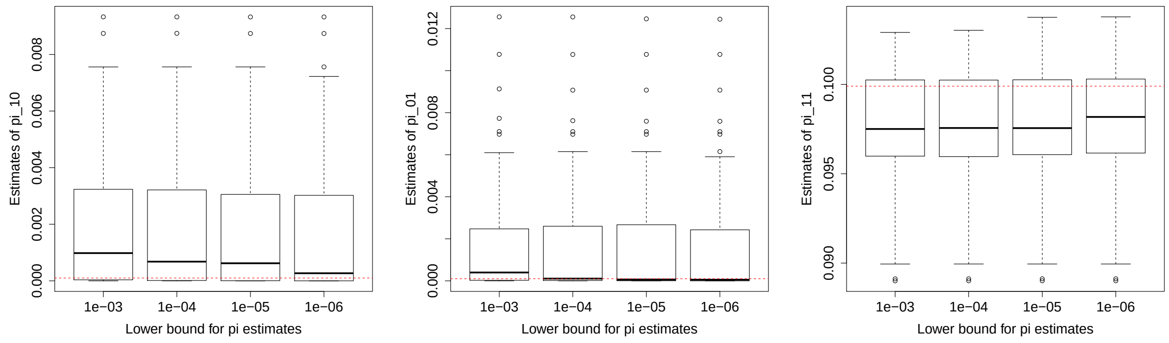

Figure S7: Estimates of π parameter for different lower bounds of π estimates in the GPA algorithm. Left panel: Estimates of π_{10}. Middle Panel: Estimates of π_{01}. Right Panel: Estimates of π_{11}.

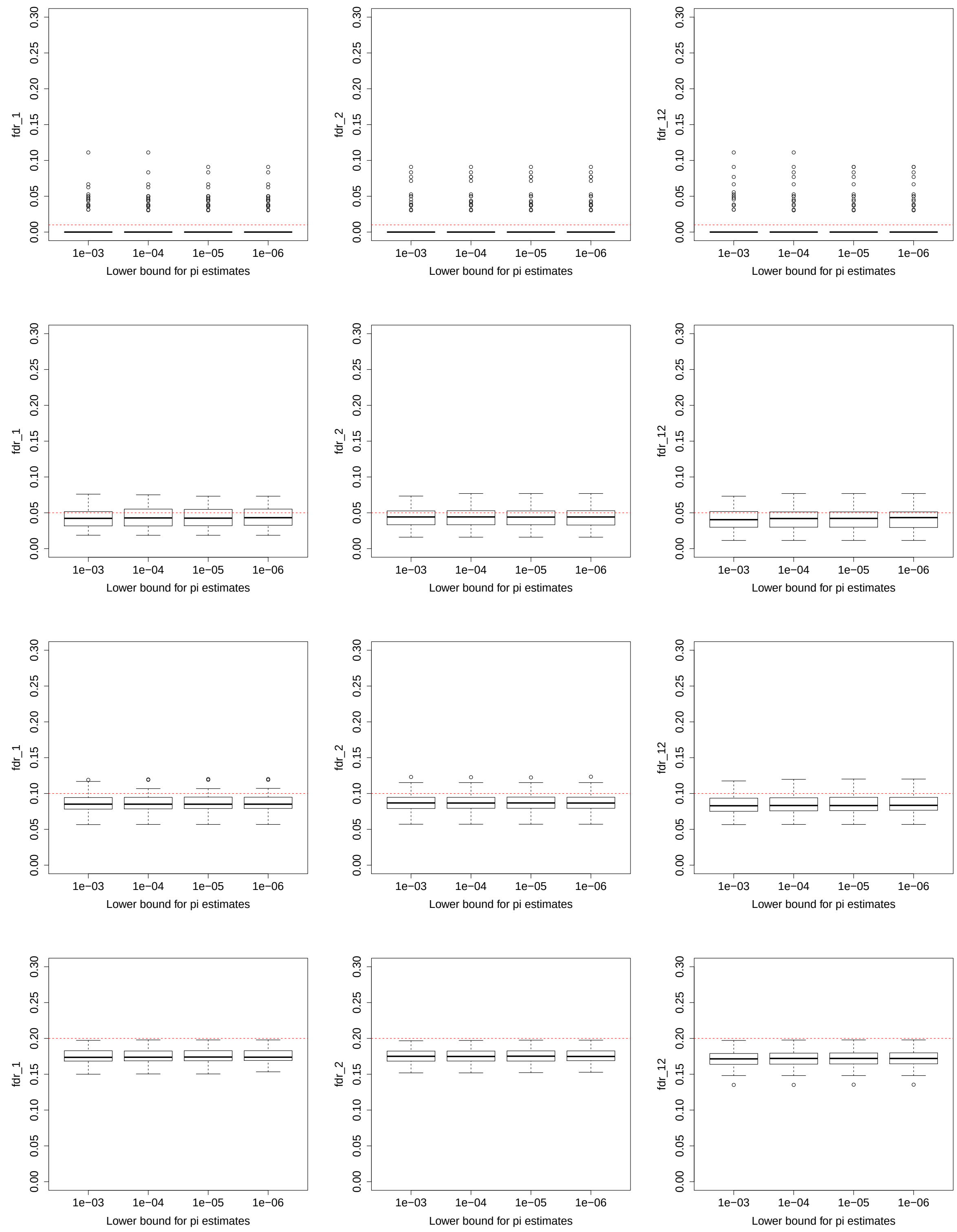

Figure S8: False discovery rates controlled at different levels for different lower bounds of π estimates. Left panel: False discovery rates for the first phenotype (fdr_1). Middle panel: False discovery rates for the second phenotype (fdr_2). Right panel: False discovery rates for both phenotypes ($\mathrm{fdr}_{1,2}$). The dashed red lines indicate the desired FDR levels.

7 Hypothesis testing of pleiotropy in the presence of annotation

In the main article, we have shown the power and type I error rate of the pleiotropy test in the absence of annotation. We used simulation to evaluate whether pleiotropy test could be affected by annotation. Comparing Figure S9 (with annotation) with Figure 5 (without annotation) in the main article, the results almost remain the same.

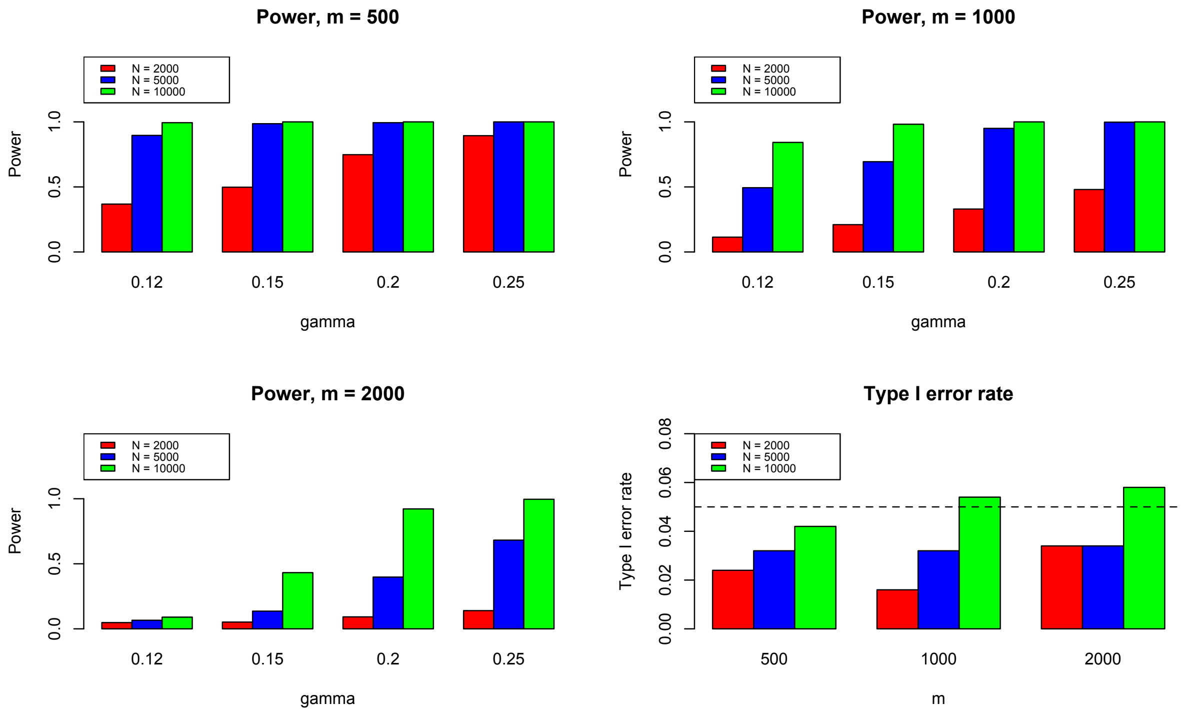

Figure S9: Hypothesis testing of pleiotropy with annotation.

8 More simulation results for different parameter settings

8.1 Performance of GPA

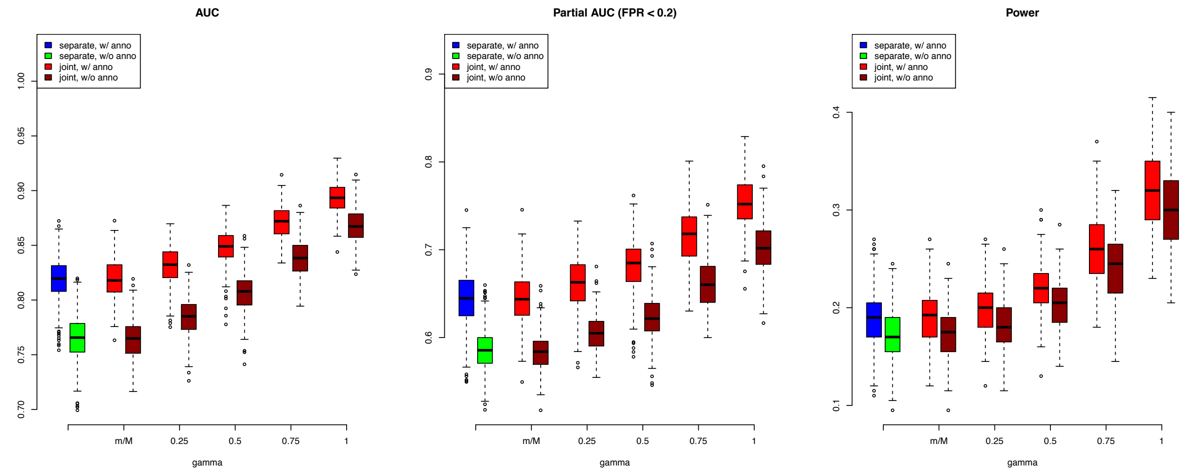

Figure S10: The AUC (left), Partial AUC (middle) and Power (right) of GPA at N=2000, m = 200.

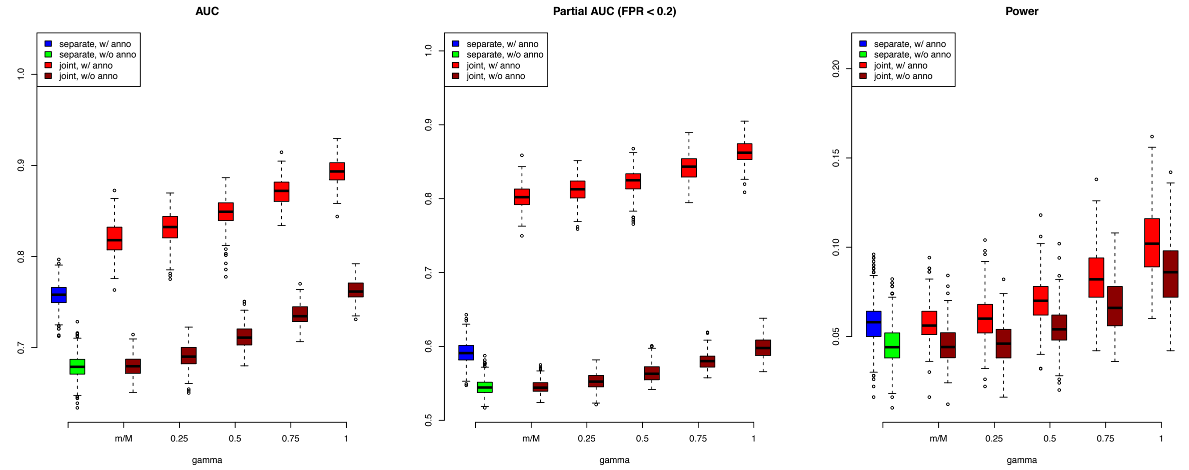

Figure S11: The AUC (left), Partial AUC (middle) and Power (right) of GPA at N=2000, m = 500.

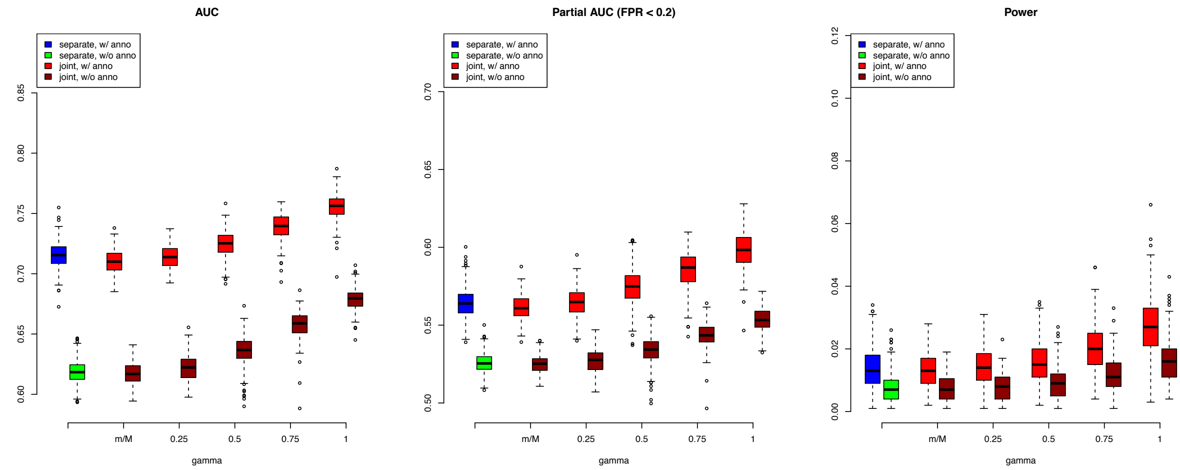

Figure S12: The AUC (left), Partial AUC (middle) and Power (right) of GPA at N=2000, m = 1000.

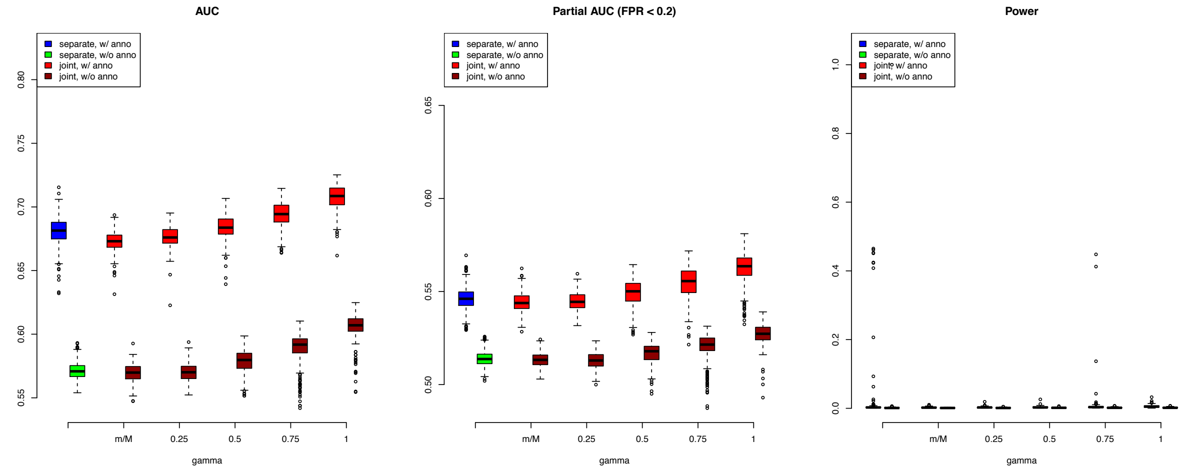

Figure S13: The AUC (left), Partial AUC (middle) and Power (right) of GPA at N=2000, m = 2000.

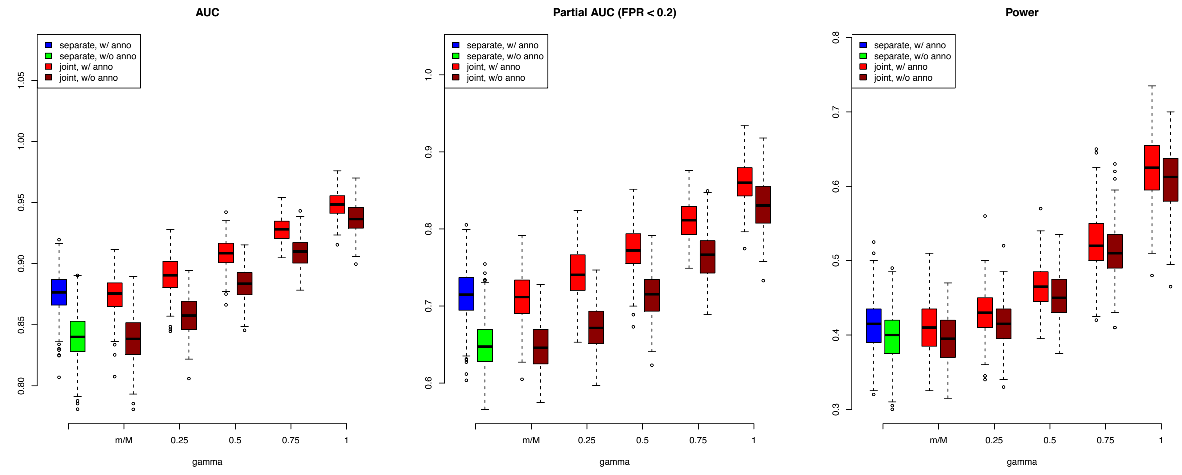

Figure S14: The AUC (left), Partial AUC (middle) and Power (right) of GPA at N=5000, m = 200.

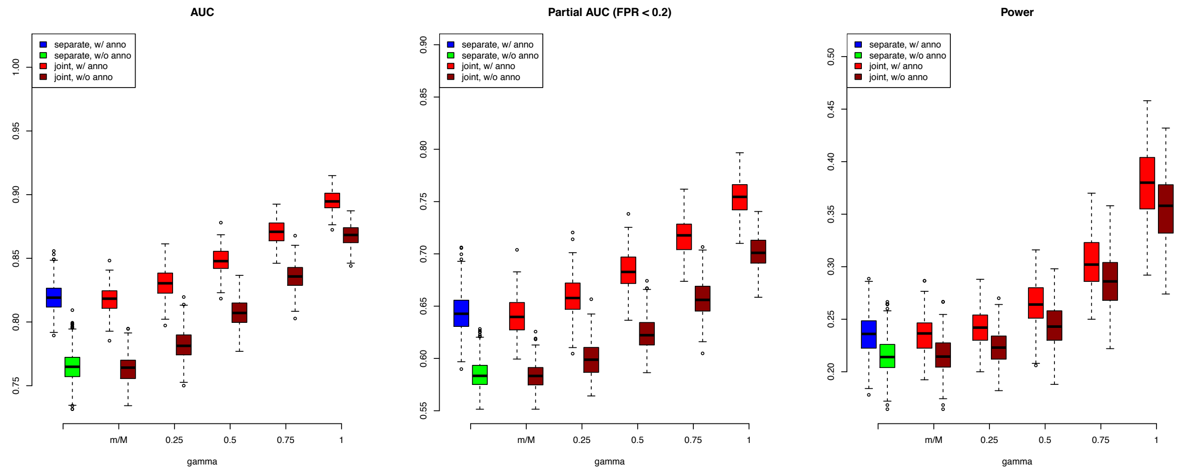

Figure S15: The AUC (left), Partial AUC (middle) and Power (right) of GPA at N=5000, m = 500.

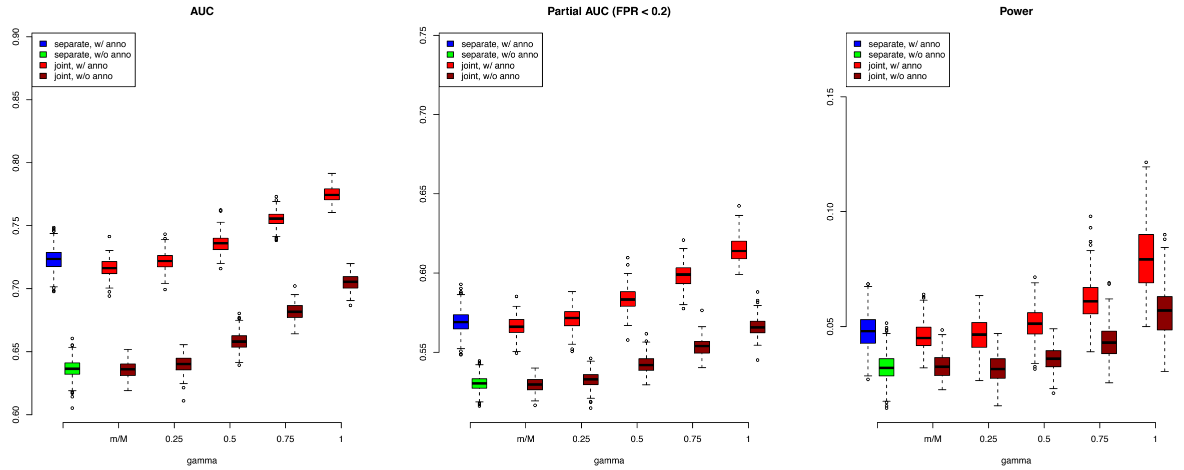

Figure S16: The AUC (left), Partial AUC (middle) and Power (right) of GPA at N=5000, m = 2000.

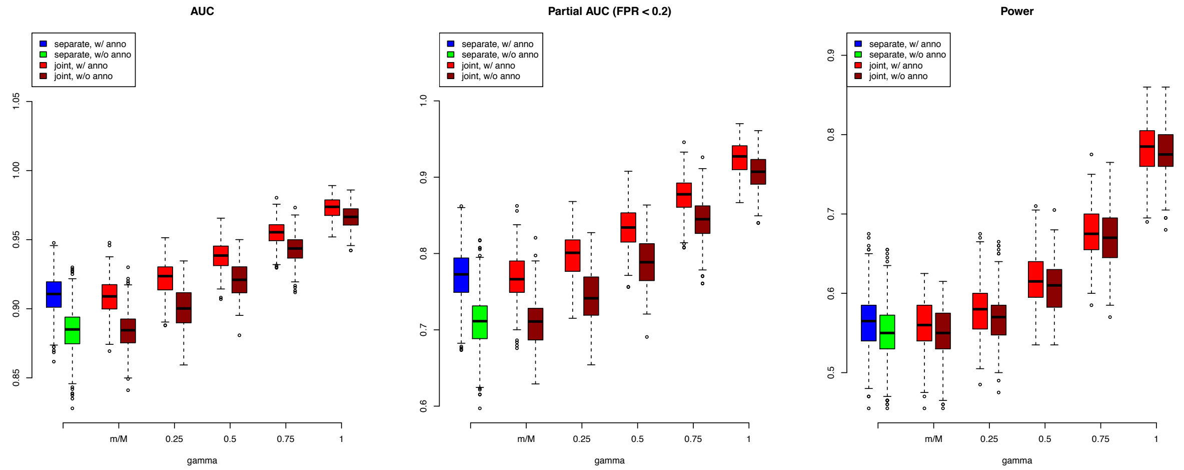

Figure S17: The AUC (left), Partial AUC (middle) and Power (right) of GPA at N=10000, m = 200.

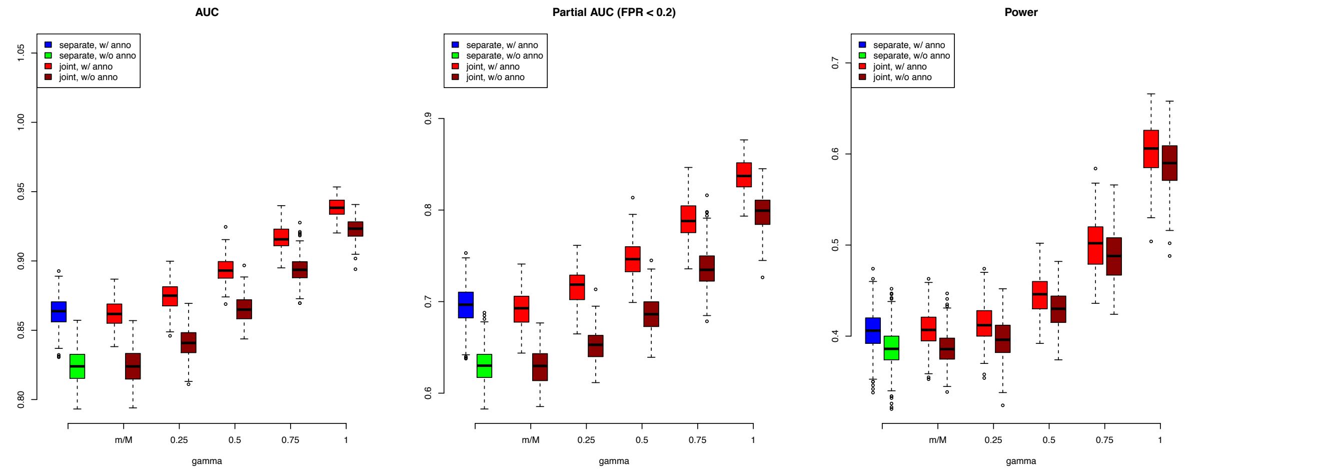

Figure S18: The AUC (left), Partial AUC (middle) and Power (right) of GPA at N=10000, m = 500.

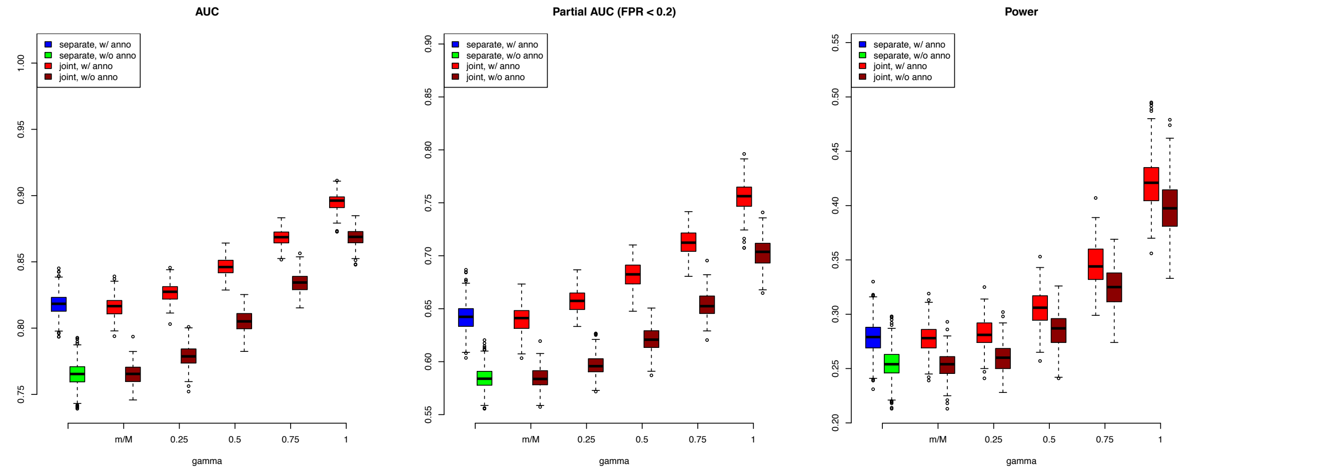

Figure S19: The AUC (left), Partial AUC (middle) and Power (right) of GPA at N=10000, m = 1000.

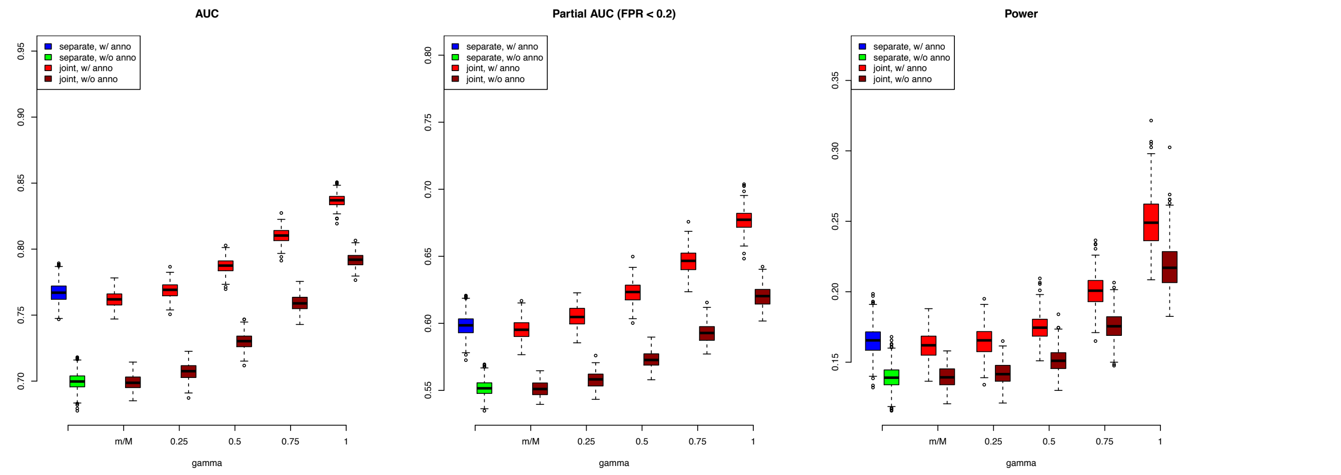

Figure S20: The AUC (left), Partial AUC (middle) and Power (right) of GPA at N=10000, m = 2000.

8.2 Evaluation of the global false discovery rates of GPA

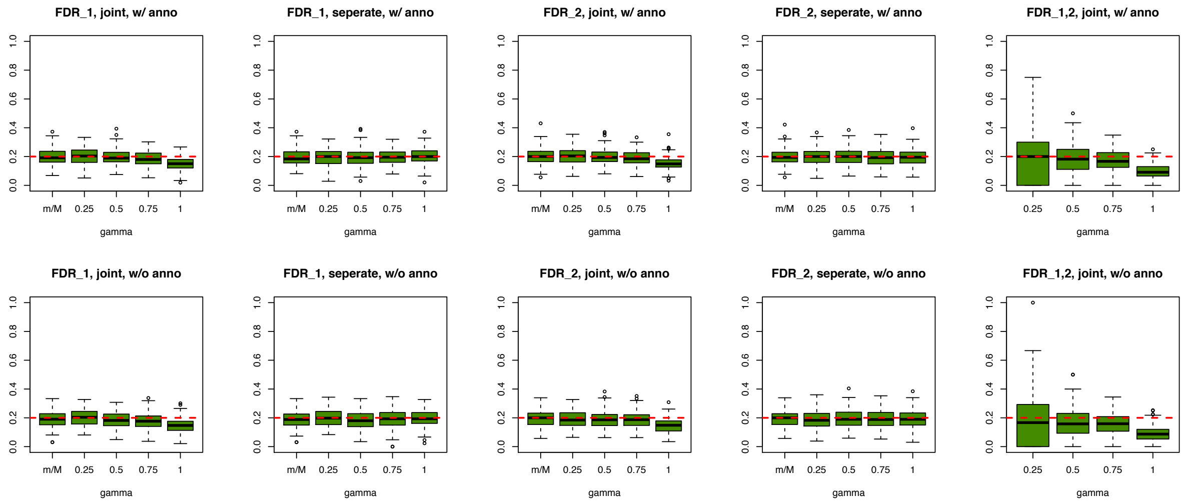

Figure S21: The global false discovery rates of GPA at $N = 2000$, $m = 200$. The upper and lower panels show the results (joint and separate analysis for the SNPs associated with the first and second diseases, as well as joint analysis for the pleiotropic SNPs) with and without annotation, respectively.

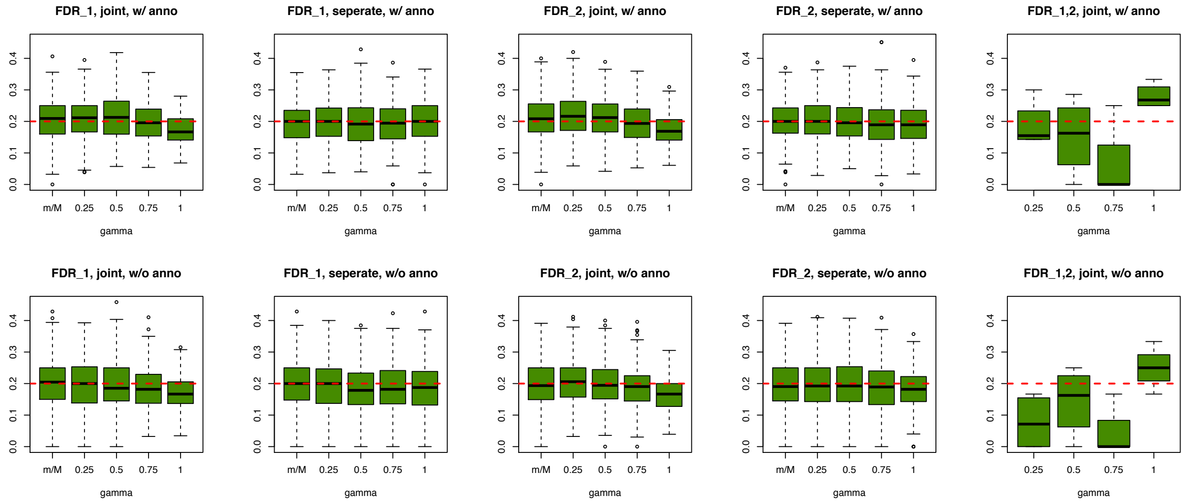

Figure S22: The global false discovery rates of GPA at $N = 2000$, $m = 500$. The upper and lower panels show the results (joint and separate analysis for the SNPs associated with the first and second diseases, as well as joint analysis for the pleiotropic SNPs) with and without annotation, respectively.

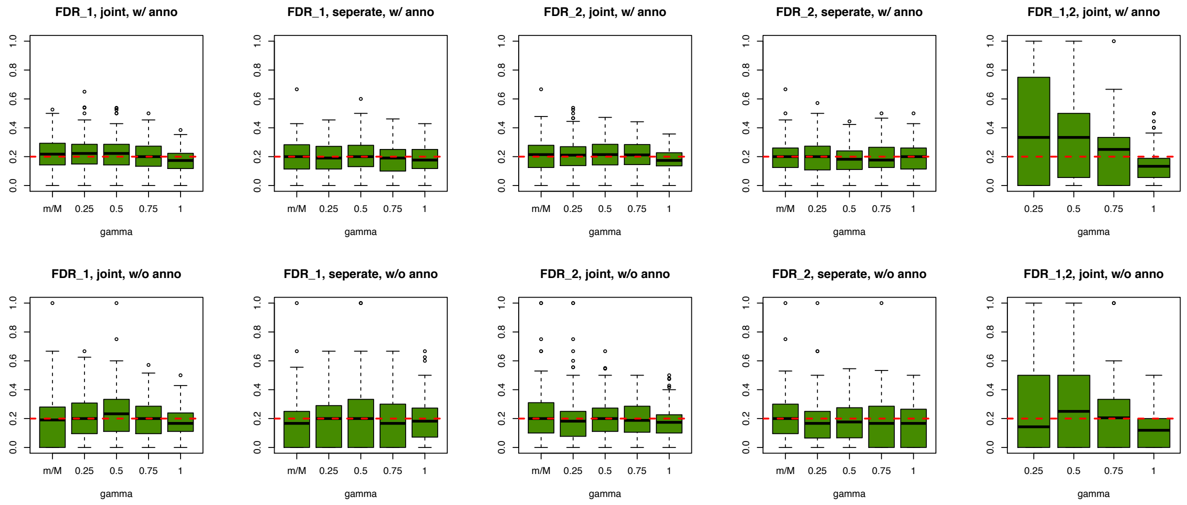

Figure S23: The global false discovery rates of GPA at $N = 2000$, $m = 1000$. The upper and lower panels show the results (joint and separate analysis for the SNPs associated with the first and second diseases, as well as joint analysis for the pleiotropic SNPs) with and without annotation, respectively.

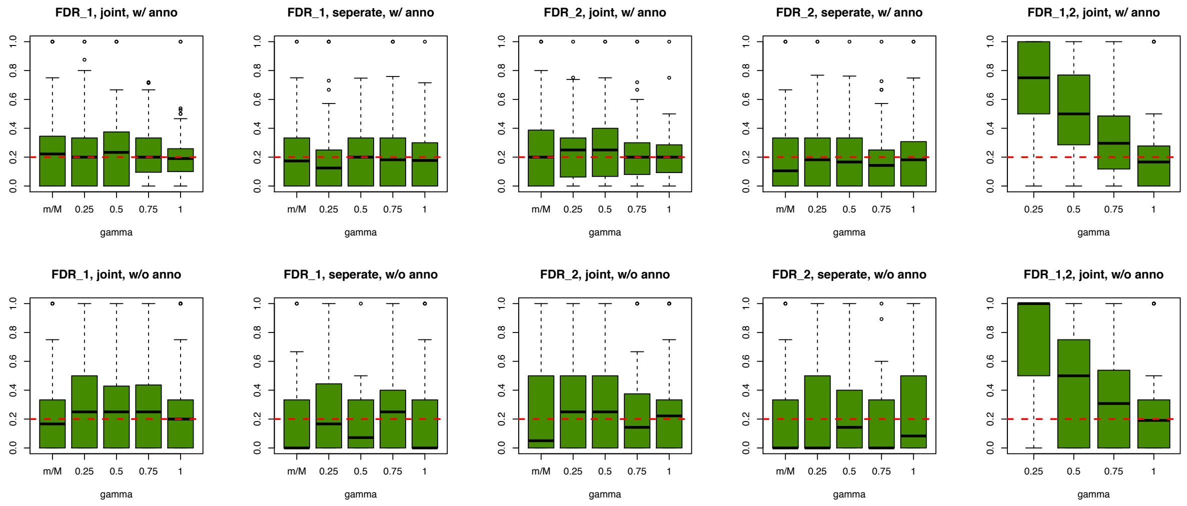

Figure S24: The global false discovery rates of GPA at $N = 2000$, $m = 2000$. The upper and lower panels show the results (joint and separate analysis for the SNPs associated with the first and second diseases, as well as joint analysis for the pleiotropic SNPs) with and without annotation, respectively.

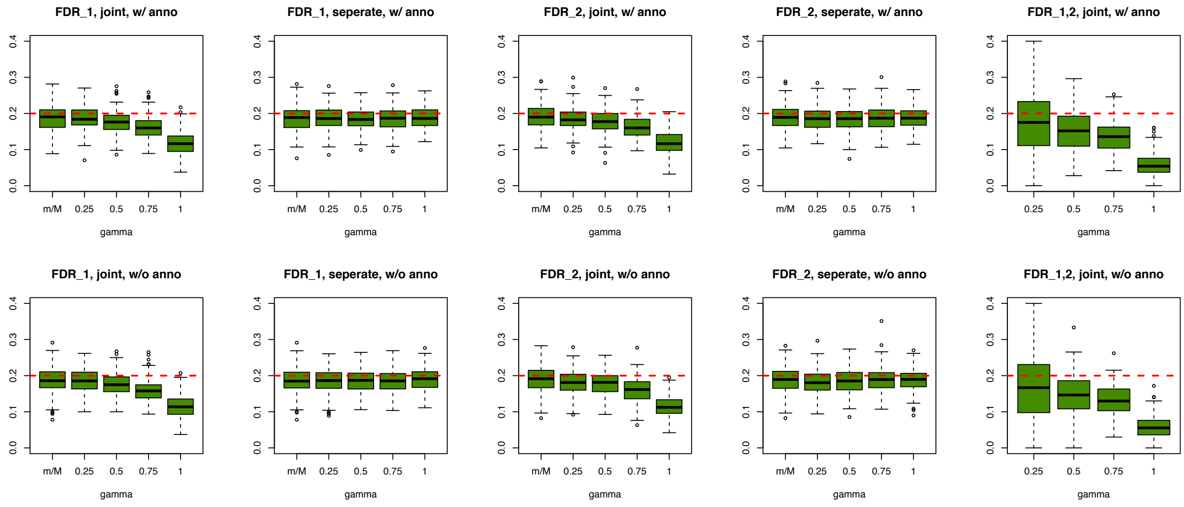

Figure S25: The global false discovery rates of GPA at $N = 5000$, $m = 200$. The upper and lower panels show the results (joint and separate analysis for the SNPs associated with the first and second diseases, as well as joint analysis for the pleiotropic SNPs) with and without annotation, respectively.

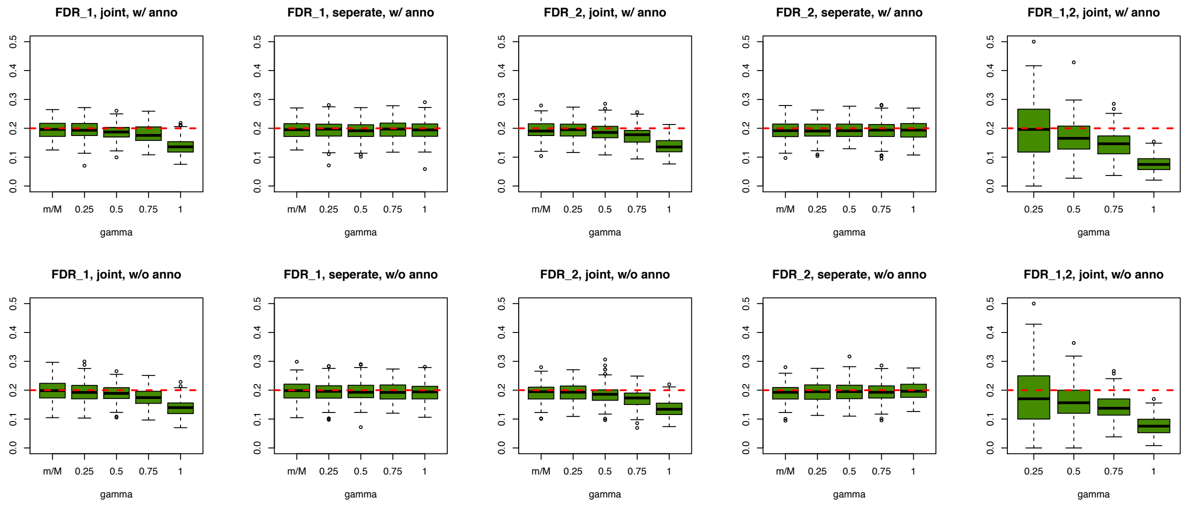

Figure S26: The global false discovery rates of GPA at $N = 5000$, $m = 500$. The upper and lower panels show the results (joint and separate analysis for the SNPs associated with the first and second diseases, as well as joint analysis for the pleiotropic SNPs) with and without annotation, respectively.

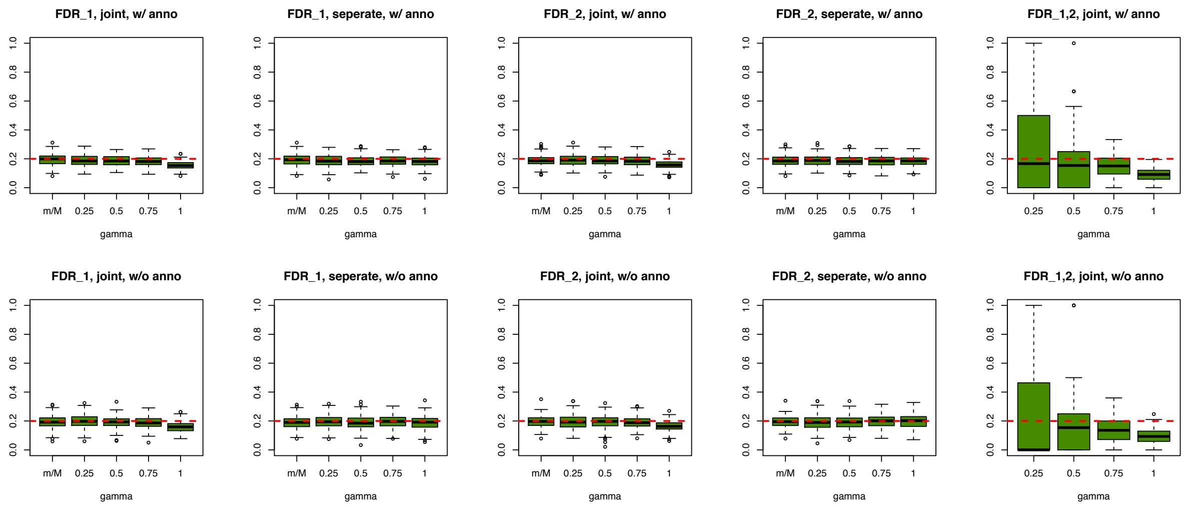

Figure S27: The global false discovery rates of GPA at $N = 5000$, $m = 2000$. The upper and lower panels show the results (joint and separate analysis for the SNPs associated with the first and second diseases, as well as joint analysis for the pleiotropic SNPs) with and without annotation, respectively.

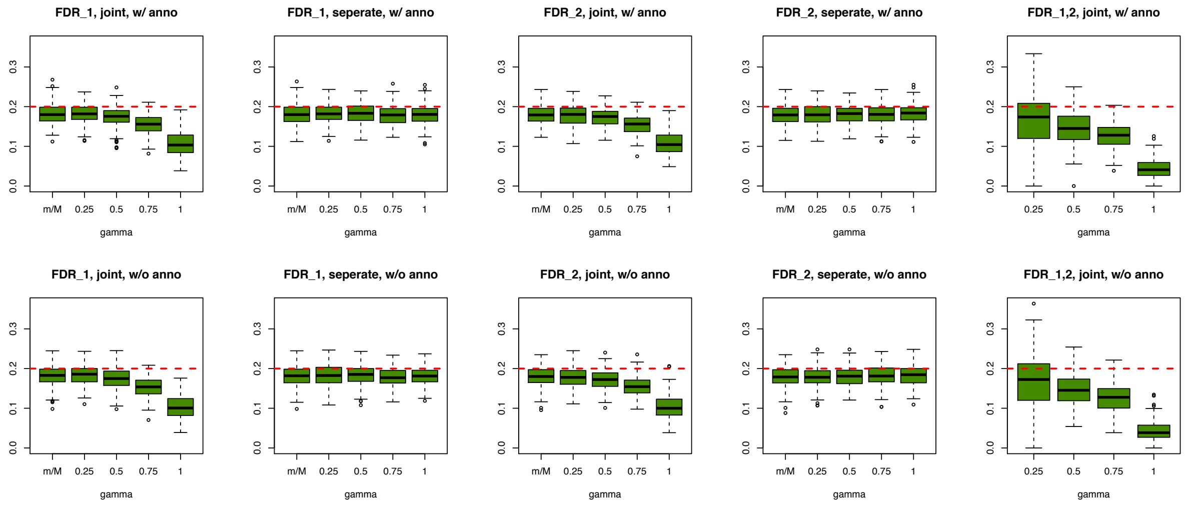

Figure S28: The global false discovery rates of GPA at $N = 10000$, $m = 200$. The upper and lower panels show the results (joint and separate analysis for the SNPs associated with the first and second diseases, as well as joint analysis for the pleiotropic SNPs) with and without annotation, respectively.

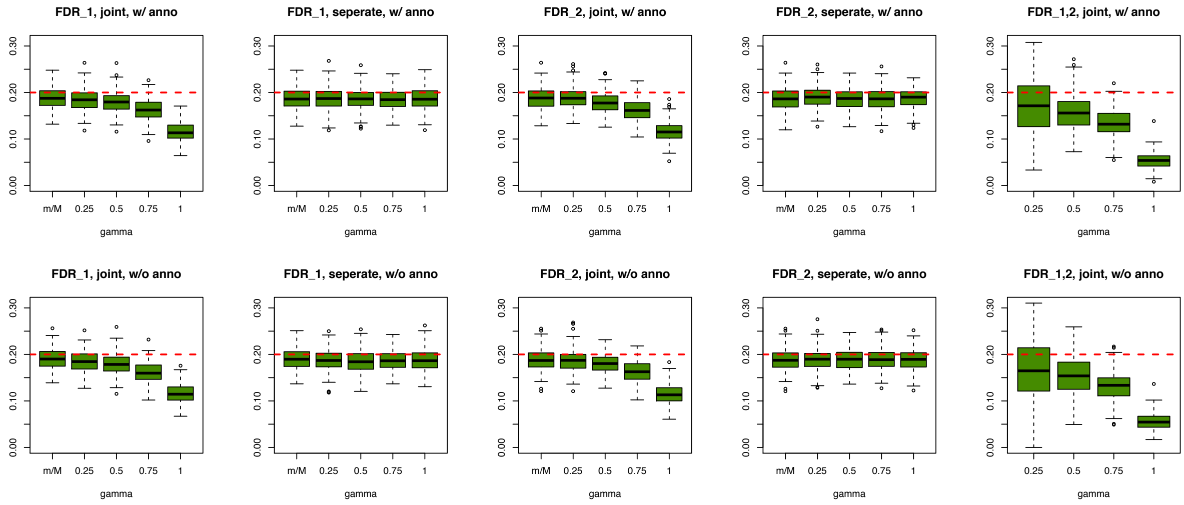

Figure S29: The global false discovery rates of GPA at $N = 10000$, $m = 500$. The upper and lower panels show the results (joint and separate analysis for the SNPs associated with the first and second diseases, as well as joint analysis for the pleiotropic SNPs) with and without annotation, respectively.

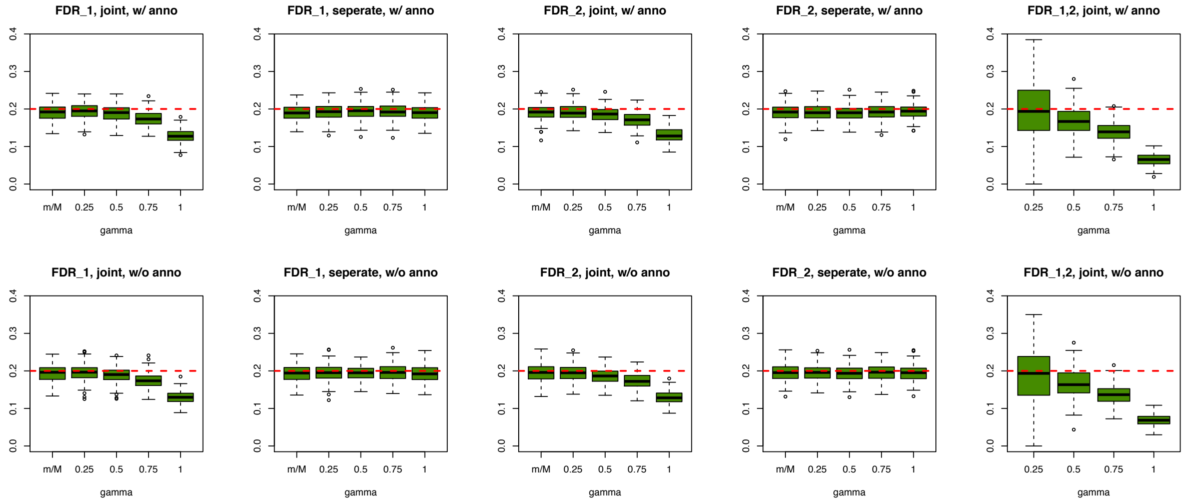

Figure S30: The global false discovery rates of GPA at $N = 10000$, $m = 1000$. The upper and lower panels show the results (joint and separate analysis for the SNPs associated with the first and second diseases, as well as joint analysis for the pleiotropic SNPs) with and without annotation, respectively.

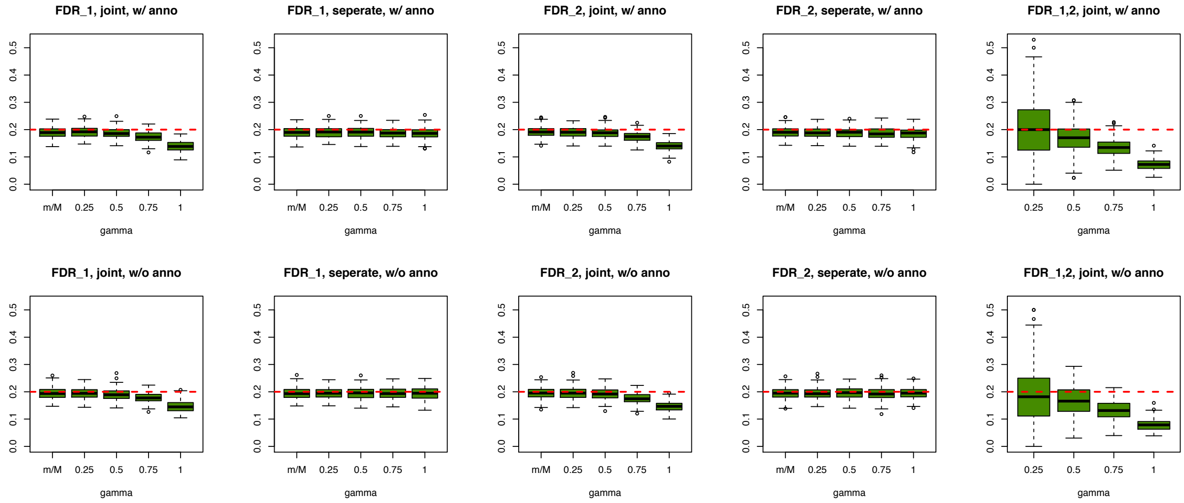

Figure S31: The global false discovery rates of GPA at $N = 10000$, $m = 2000$. The upper and lower panels show the results (joint and separate analysis for the SNPs associated with the first and second diseases, as well as joint analysis for the pleiotropic SNPs) with and without annotation, respectively.

8.3 Parameter $\{q_l\}$ estimation

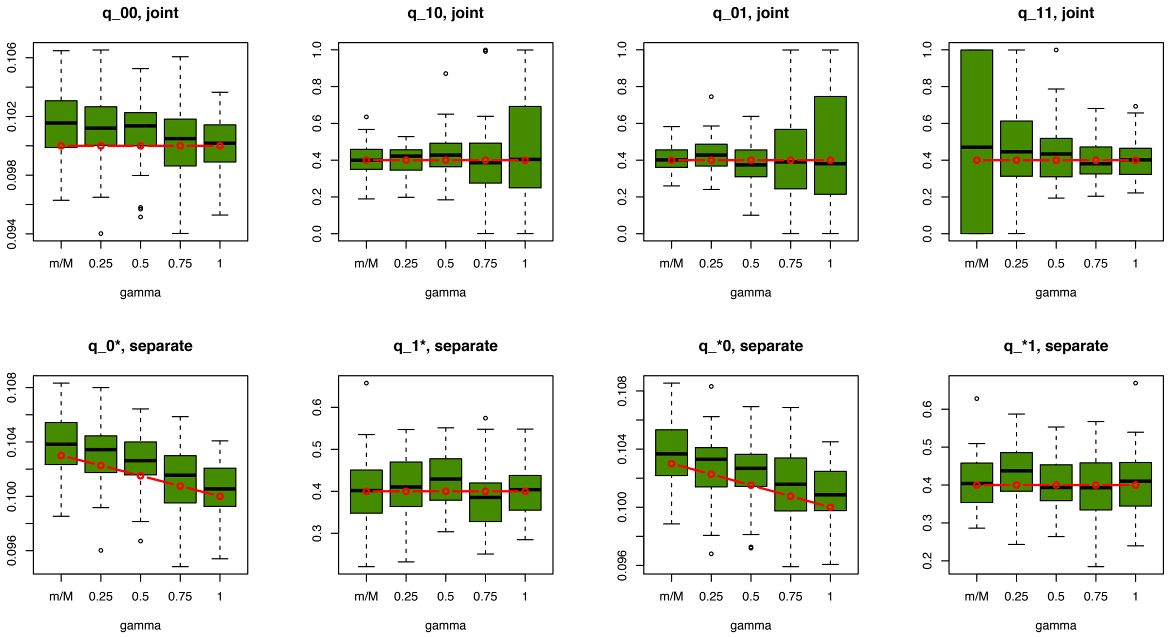

Figure S32: The estimated q at $N = 2000$, $m = 200$. The upper panel shows the estimated q_{00}, q_{10}, q_{01}, q_{11} in the joint analysis of two GWAS. The lower panel shows the estimated q_{0*}, q_{1*} and q_{*0}, q_{*1} in the separate analysis for the first and second GWAS, respectively. The red line represents the true values.

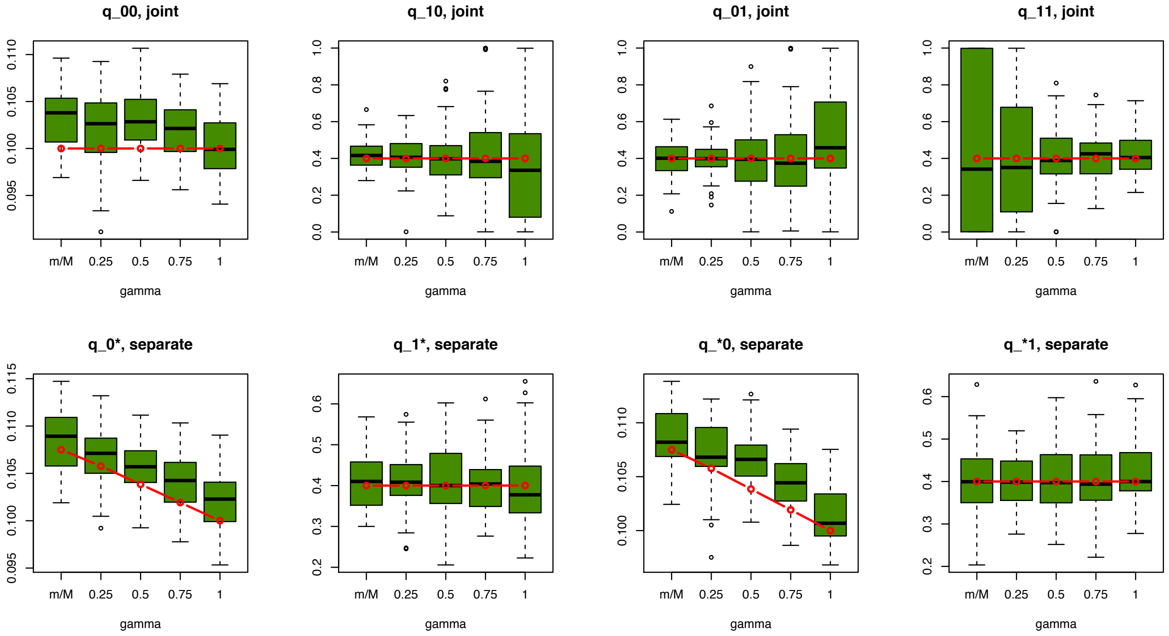

Figure S33: The estimated q at $N = 2000$, $m = 200$. The upper panel shows the estimated q_{00}, q_{10}, q_{01}, q_{11} in the joint analysis of two GWAS. The lower panel shows the estimated q_{0*}, q_{1*} and q_{*0}, q_{*1} in the separate analysis for the first and second GWAS, respectively. The red line represents the true values.

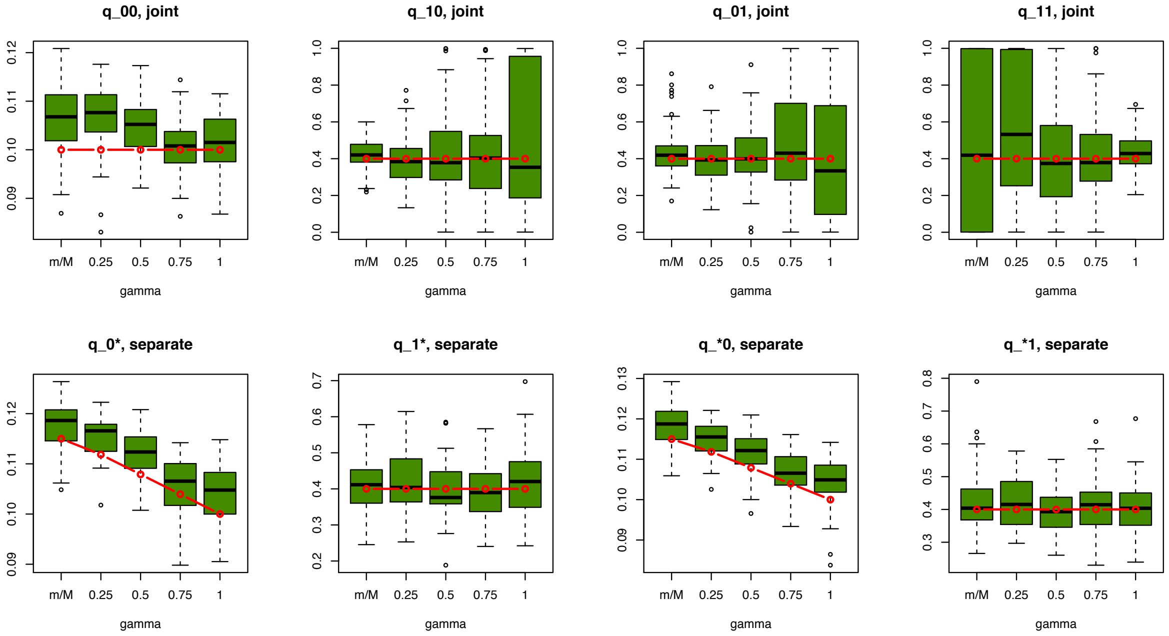

Figure S34: The estimated q at $N = 2000$, $m = 200$. The upper panel shows the estimated q_{00}, q_{10}, q_{01}, q_{11} in the joint analysis of two GWAS. The lower panel shows the estimated q_{0*}, q_{1*} and q_{*0}, q_{*1} in the separate analysis for the first and second GWAS, respectively. The red line represents the true values.

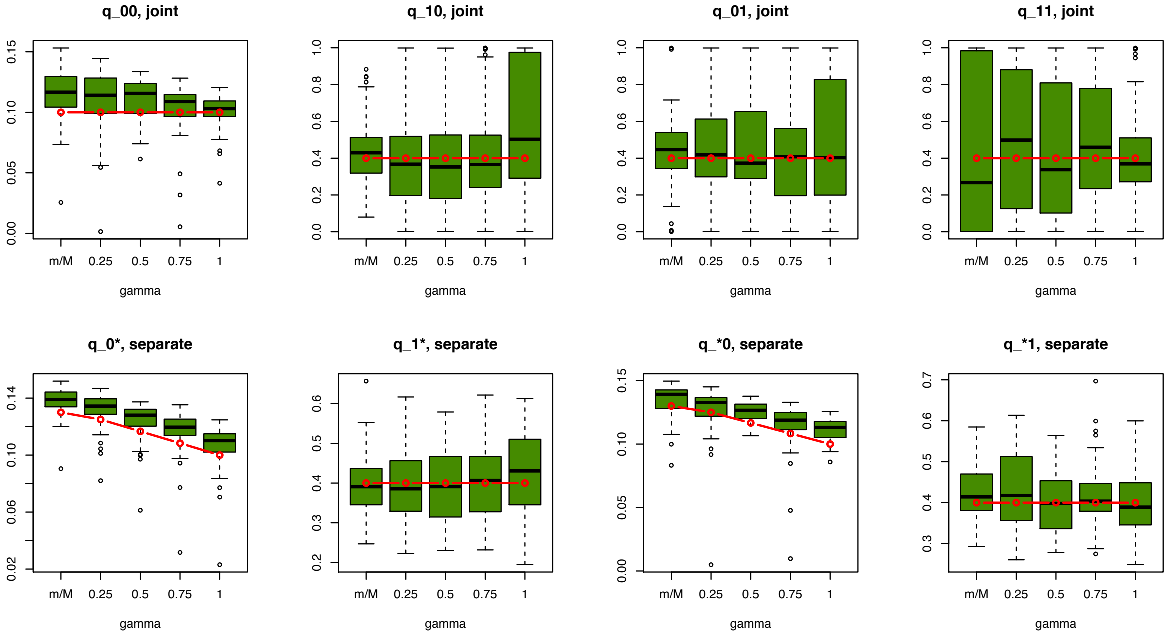

Figure S35: The estimated q at $N = 2000$, $m = 200$. The upper panel shows the estimated q_{00}, q_{10}, q_{01}, q_{11} in the joint analysis of two GWAS. The lower panel shows the estimated q_{0*}, q_{1*} and q_{*0}, q_{*1} in the separate analysis for the first and second GWAS, respectively. The red line represents the true values.

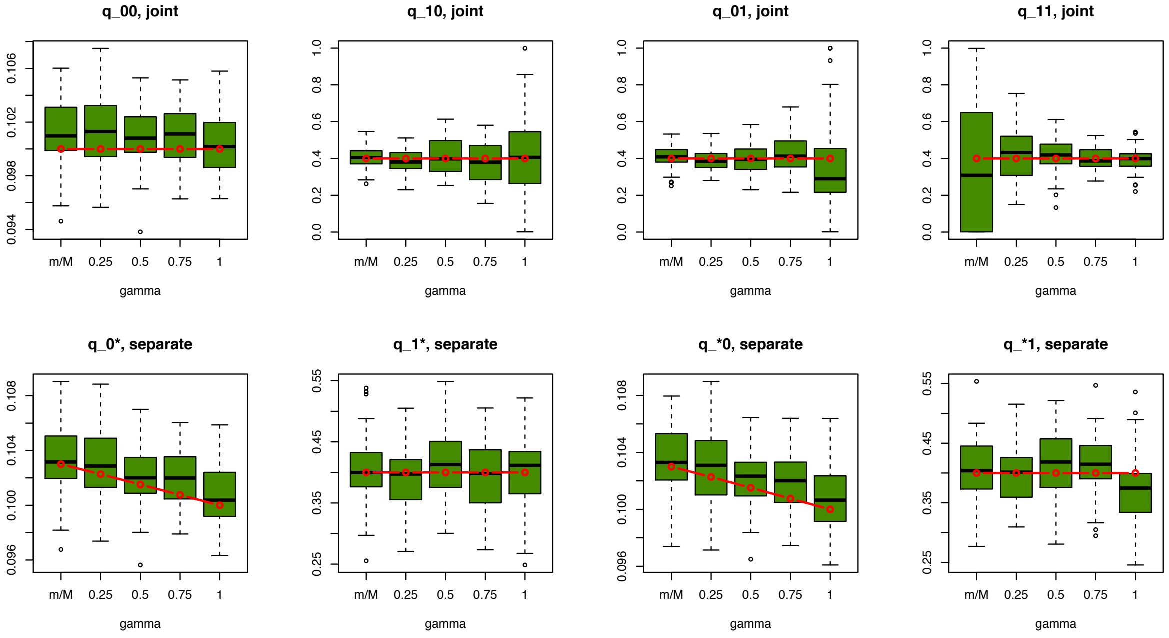

Figure S36: The estimated q at $N = 2000$, $m = 200$. The upper panel shows the estimated q_{00}, q_{10}, q_{01}, q_{11} in the joint analysis of two GWAS. The lower panel shows the estimated q_{0*}, q_{1*} and q_{*0}, q_{*1} in the separate analysis for the first and second GWAS, respectively. The red line represents the true values.

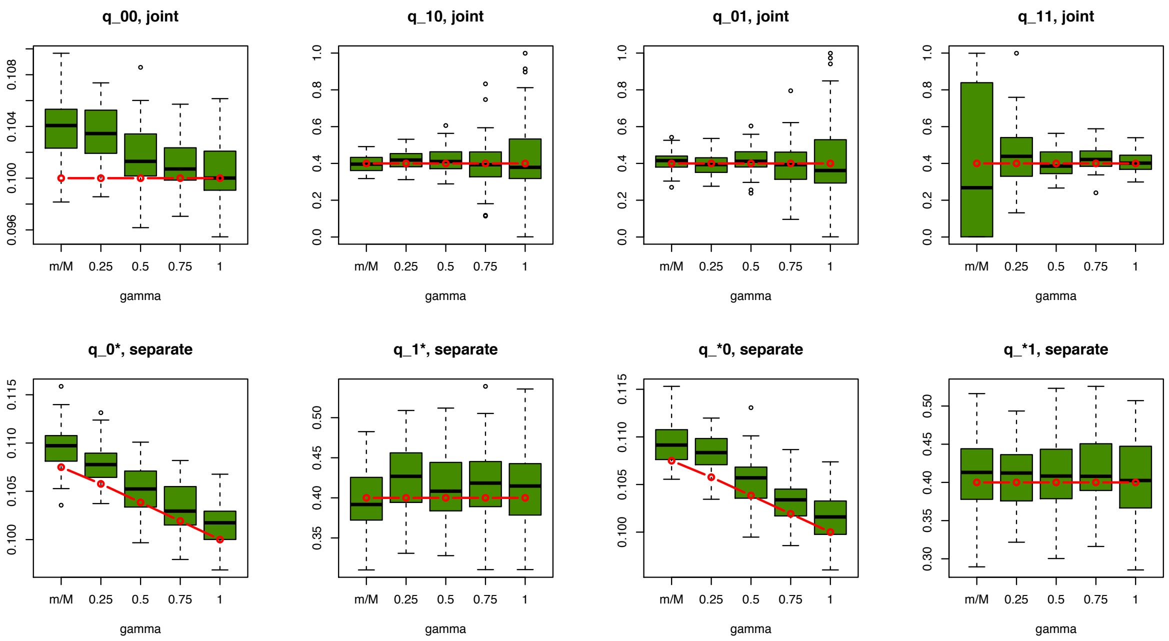

Figure S37: The estimated q at $N = 2000$, $m = 200$. The upper panel shows the estimated q_{00}, q_{10}, q_{01}, q_{11} in the joint analysis of two GWAS. The lower panel shows the estimated q_{0*}, q_{1*} and q_{*0}, q_{*1} in the separate analysis for the first and second GWAS, respectively. The red line represents the true values.

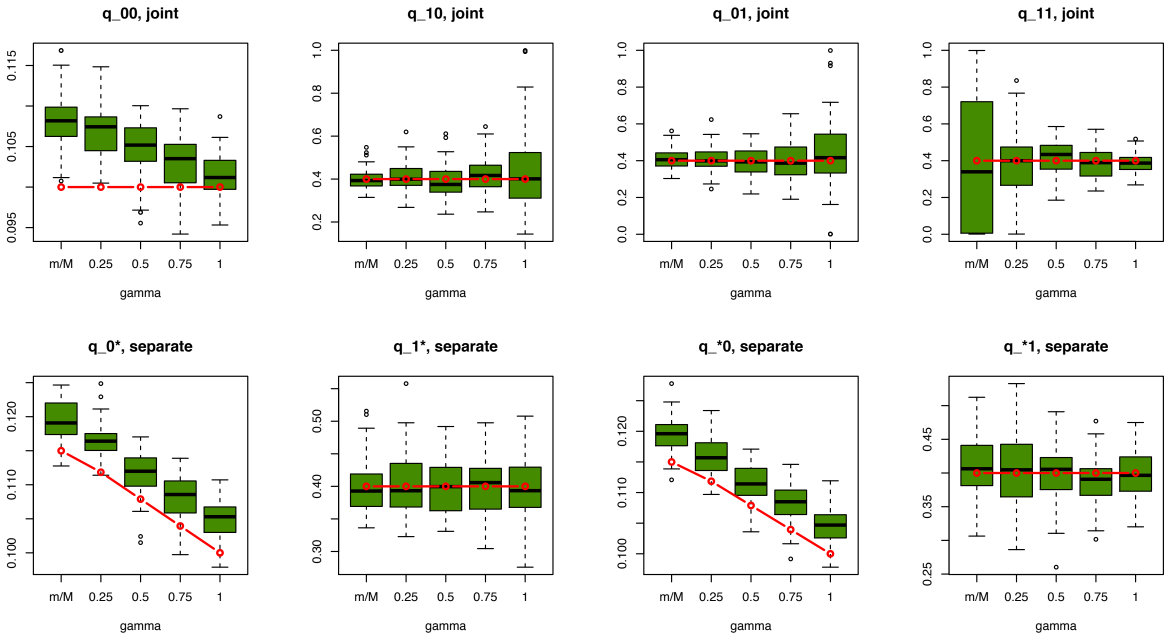

Figure S38: The estimated q at $N = 2000$, $m = 200$. The upper panel shows the estimated q_{00}, q_{10}, q_{01}, q_{11} in the joint analysis of two GWAS. The lower panel shows the estimated q_{0*}, q_{1*} and q_{*0}, q_{*1} in the separate analysis for the first and second GWAS, respectively. The red line represents the true values.

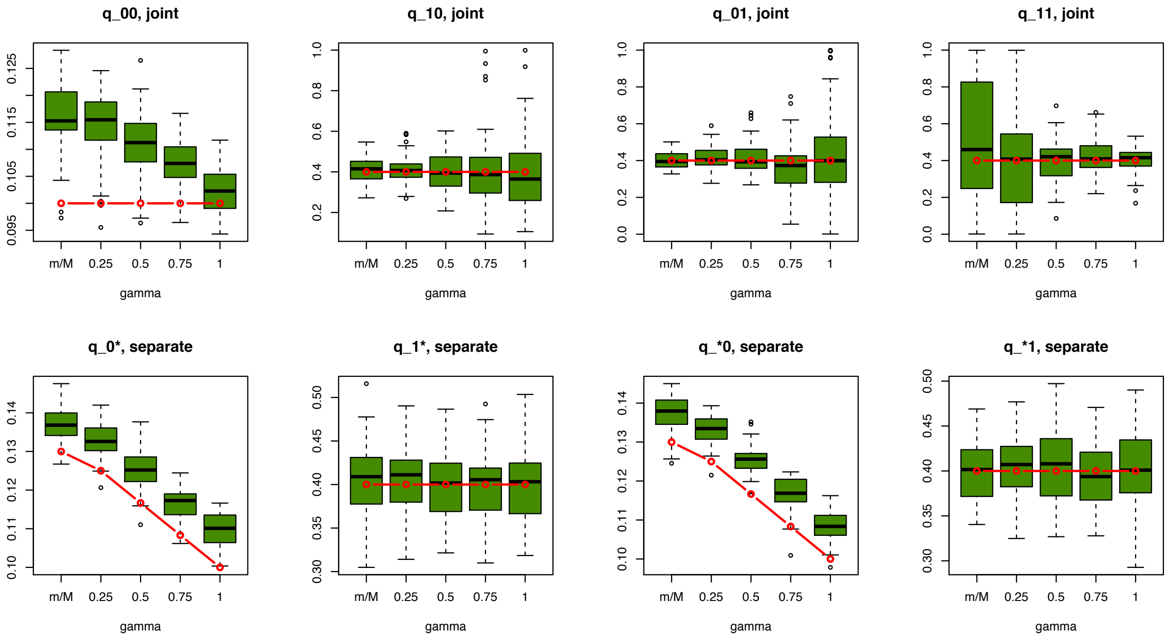

Figure S39: The estimated q at $N = 2000$, $m = 200$. The upper panel shows the estimated q_{00}, q_{10}, q_{01}, q_{11} in the joint analysis of two GWAS. The lower panel shows the estimated q_{0*}, q_{1*} and q_{*0}, q_{*1} in the separate analysis for the first and second GWAS, respectively. The red line represents the true values.

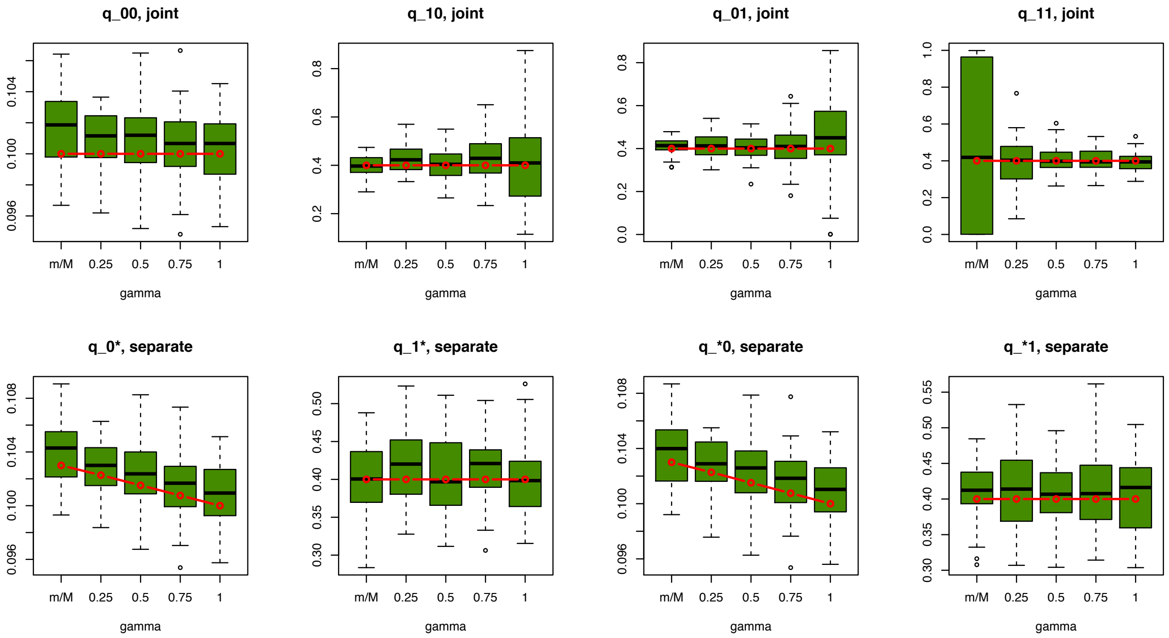

Figure S40: The estimated q at $N = 2000$, $m = 200$. The upper panel shows the estimated q_{00}, q_{10}, q_{01}, q_{11} in the joint analysis of two GWAS. The lower panel shows the estimated q_{0*}, q_{1*} and q_{*0}, q_{*1} in the separate analysis for the first and second GWAS, respectively. The red line represents the true values.

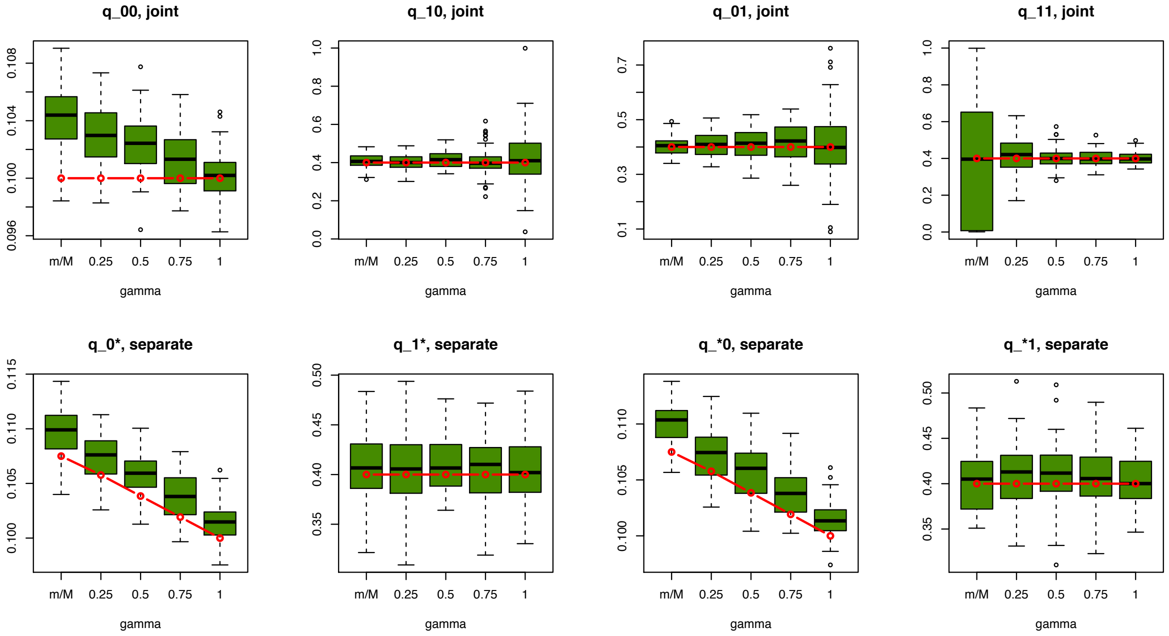

Figure S41: The estimated q at $N = 2000$, $m = 200$. The upper panel shows the estimated q_{00}, q_{10}, q_{01}, q_{11} in the joint analysis of two GWAS. The lower panel shows the estimated q_{0*}, q_{1*} and q_{*0}, q_{*1} in the separate analysis for the first and second GWAS, respectively. The red line represents the true values.

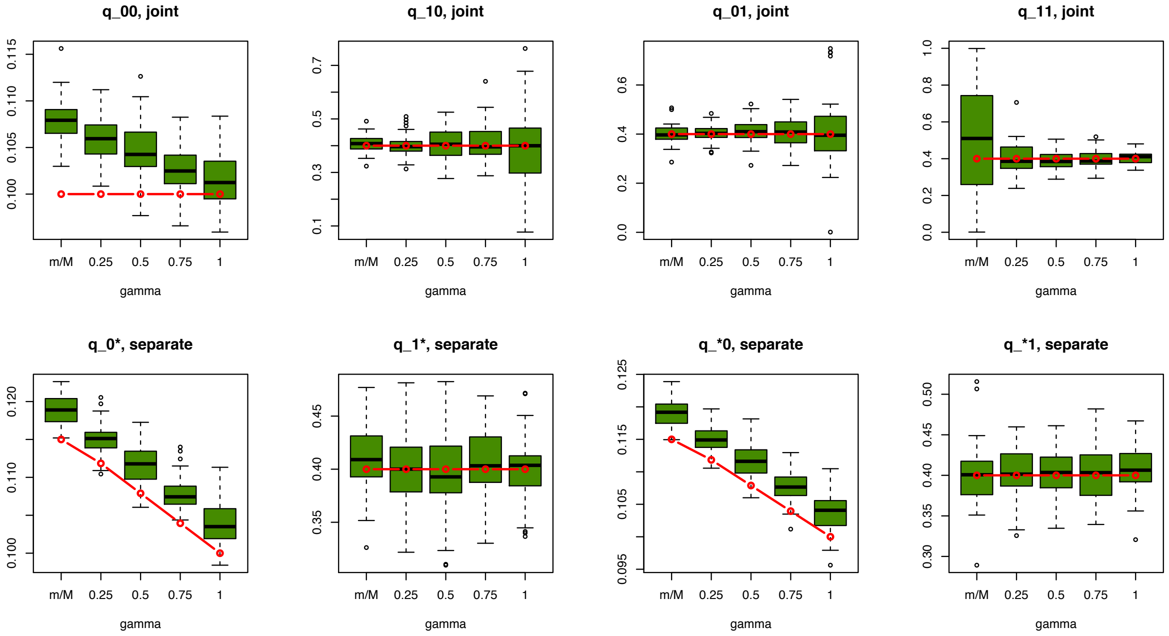

Figure S42: The estimated q at $N = 2000$, $m = 200$. The upper panel shows the estimated q_{00}, q_{10}, q_{01}, q_{11} in the joint analysis of two GWAS. The lower panel shows the estimated q_{0*}, q_{1*} and q_{*0}, q_{*1} in the separate analysis for the first and second GWAS, respectively. The red line represents the true values.

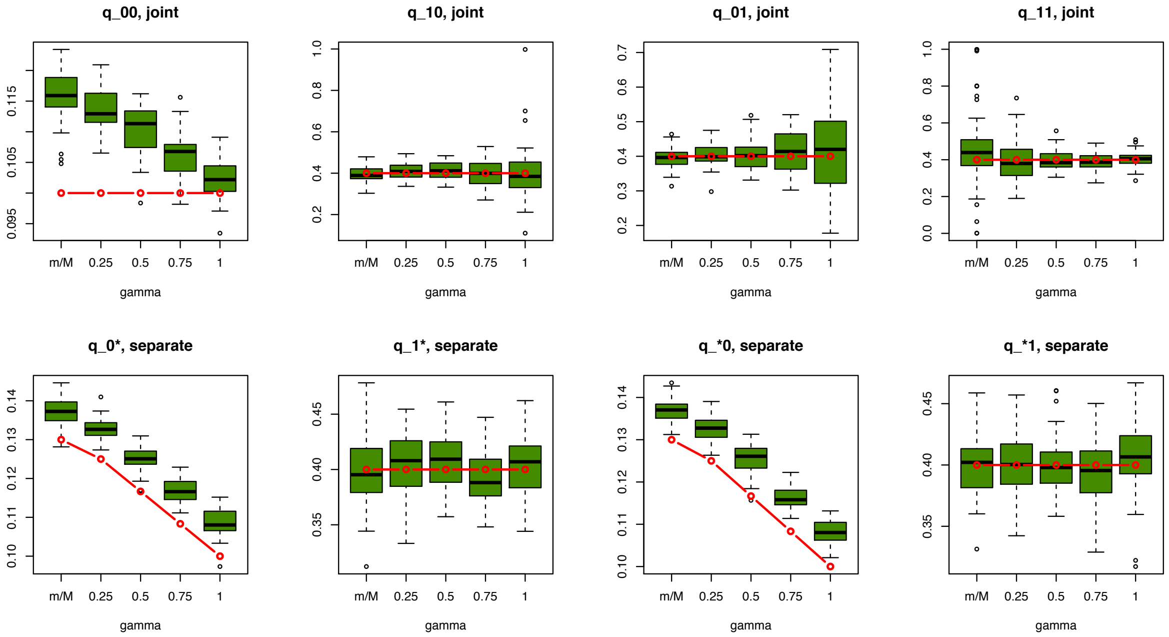

Figure S43: The estimated q at $N = 2000$, $m = 200$. The upper panel shows the estimated q_{00}, q_{10}, q_{01}, q_{11} in the joint analysis of two GWAS. The lower panel shows the estimated q_{0*}, q_{1*} and q_{*0}, q_{*1} in the separate analysis for the first and second GWAS, respectively. The red line represents the true values.

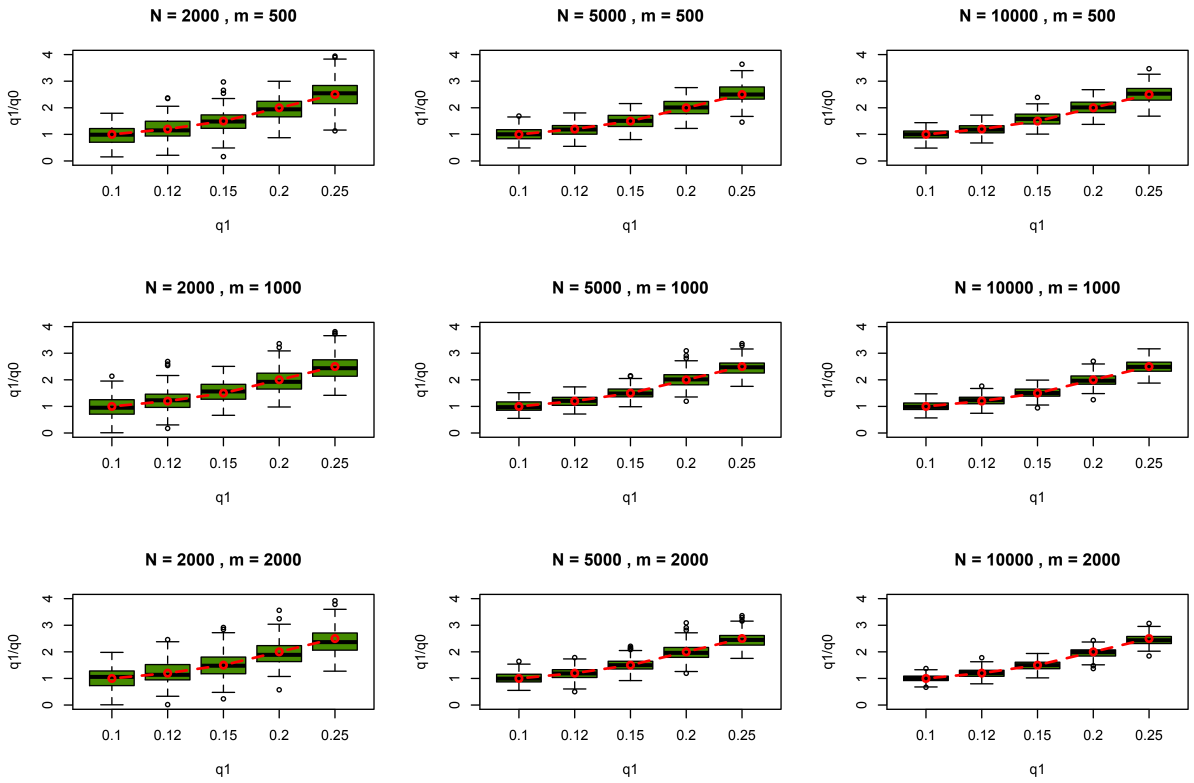

Figure S44: The fold of annotation enrichment q_1/q_0 estimation with sample size $N = 2000, 5000, 10000$ and number of risk SNPs $m = 500, 1000, 2000$. Here the red dotted line indicates the true value.

8.4 Parameter $\{\pi_l\}$ estimation in separate analysis (single GWAS) based on the random-effects model

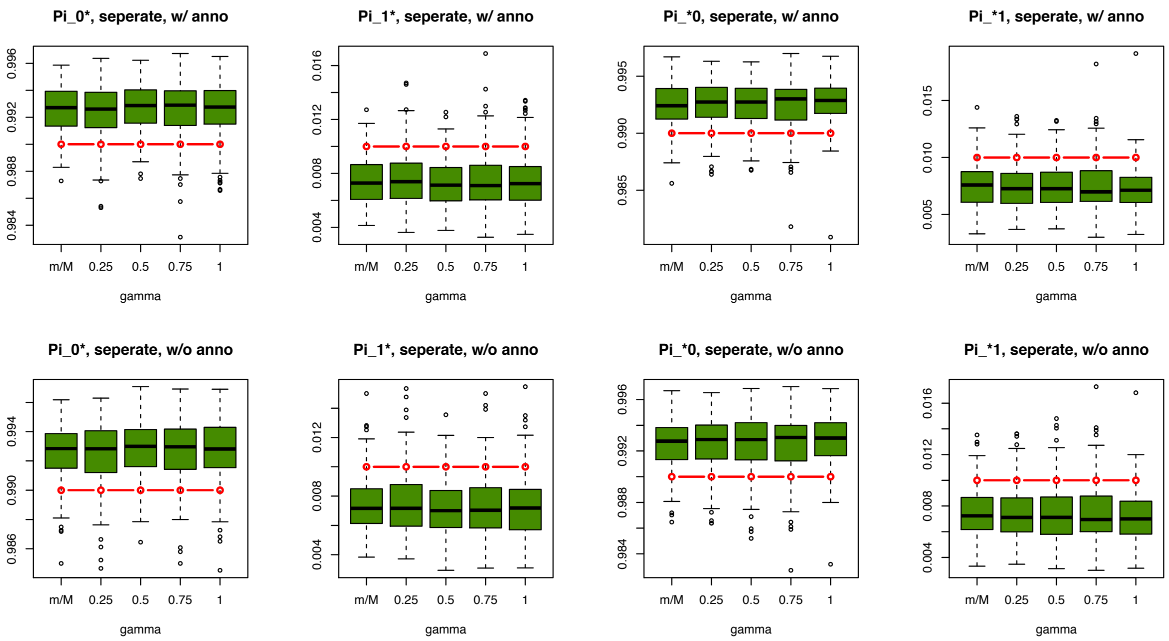

Figure S45: The estimated $\{\pi_l\}$ of separate analysis (single GWAS) at $N = 2000$, $m = 200$. The upper panel shows the estimated π_{0*}, π_{1*} and π_{*0}, π_{*1} with annotation, and the lower panel shows the estimated π_{0*}, π_{1*} and π_{*0}, π_{*1} without annotation. The red lines represent the true values.

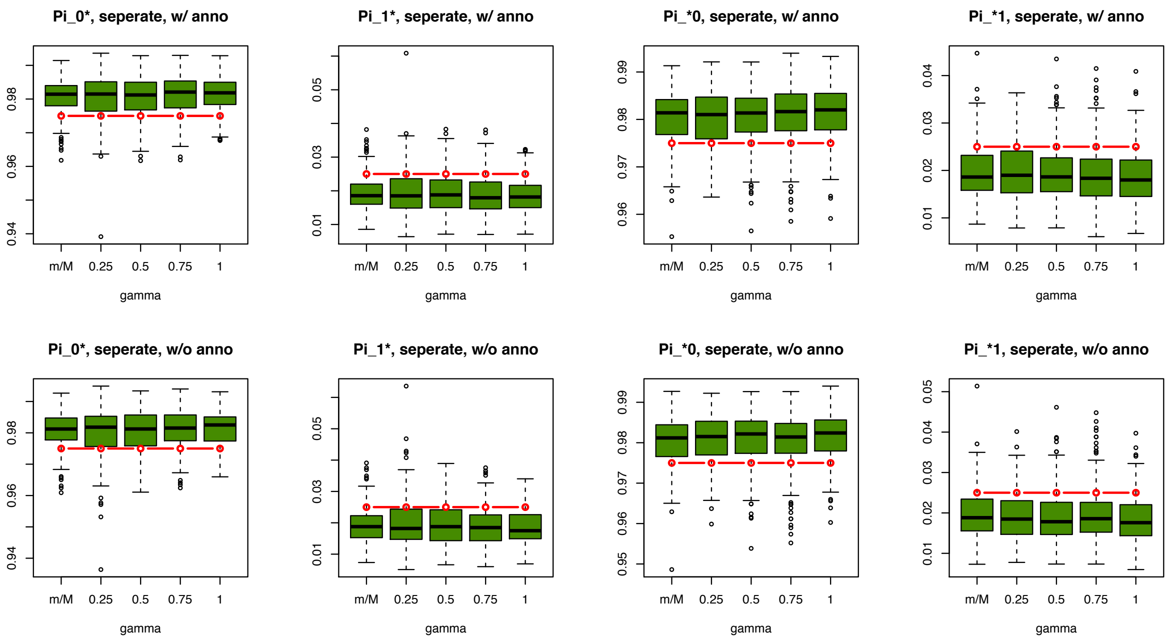

Figure S46: The estimated $\{\pi_l\}$ of separate analysis (single GWAS) at $N = 2000$, $m = 500$. The upper panel shows the estimated π_{0*}, π_{1*} and π_{*0}, π_{*1} with annotation, and the lower panel shows the estimated π_{0*}, π_{1*} and π_{*0}, π_{*1} without annotation. The red lines represent the true values.

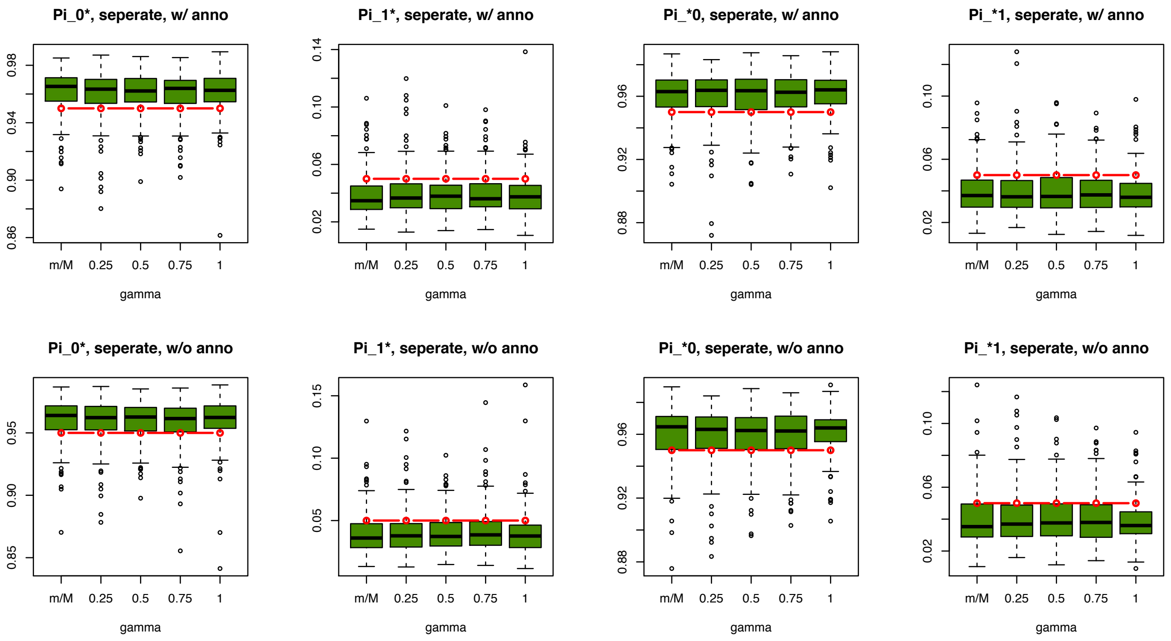

Figure S47: The estimated $\{\pi_l\}$ of separate analysis (single GWAS) at $N = 2000$, $m = 1000$. The upper panel shows the estimated π_{0*}, π_{1*} and π_{*0}, π_{*1} with annotation, and the lower panel shows the estimated π_{0*}, π_{1*} and π_{*0}, π_{*1} without annotation. The red lines represent the true values.

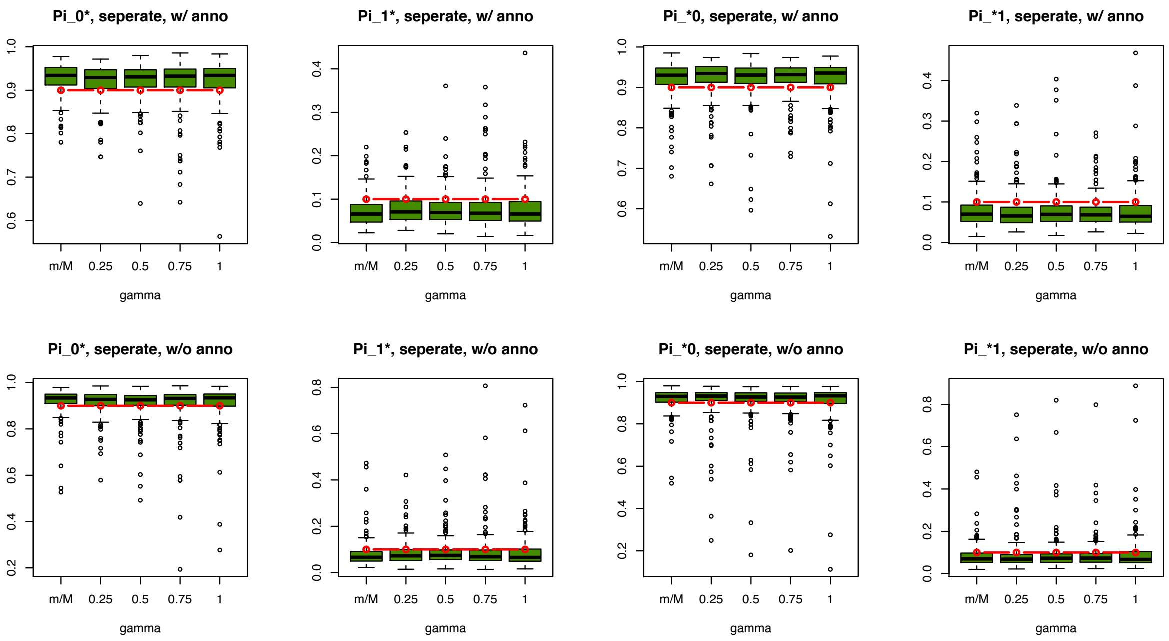

Figure S48: The estimated $\{\pi_l\}$ of separate analysis (single GWAS) at $N = 2000$, $m = 2000$. The upper panel shows the estimated π_{0*}, π_{1*} and π_{*0}, π_{*1} with annotation, and the lower panel shows the estimated π_{0*}, π_{1*} and π_{*0}, π_{*1} without annotation. The red lines represent the true values.

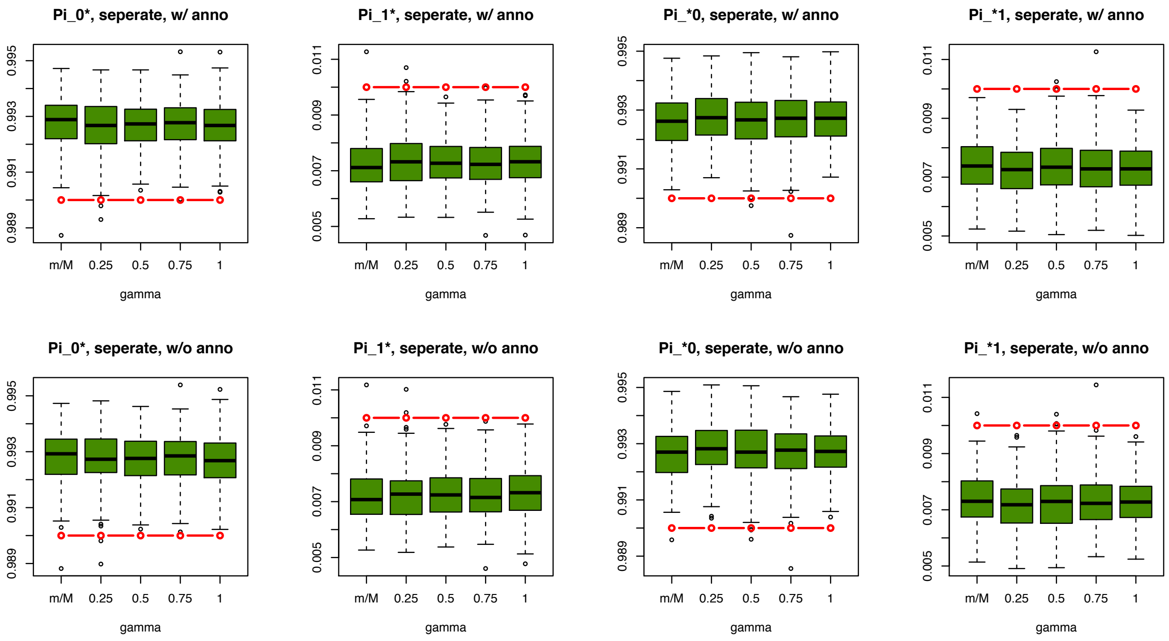

Figure S49: The estimated $\{\pi_l\}$ of separate analysis (single GWAS) at $N = 5000$, $m = 200$. The upper panel shows the estimated π_{0*}, π_{1*} and π_{*0}, π_{*1} with annotation, and the lower panel shows the estimated π_{0*}, π_{1*} and π_{*0}, π_{*1} without annotation. The red lines represent the true values.

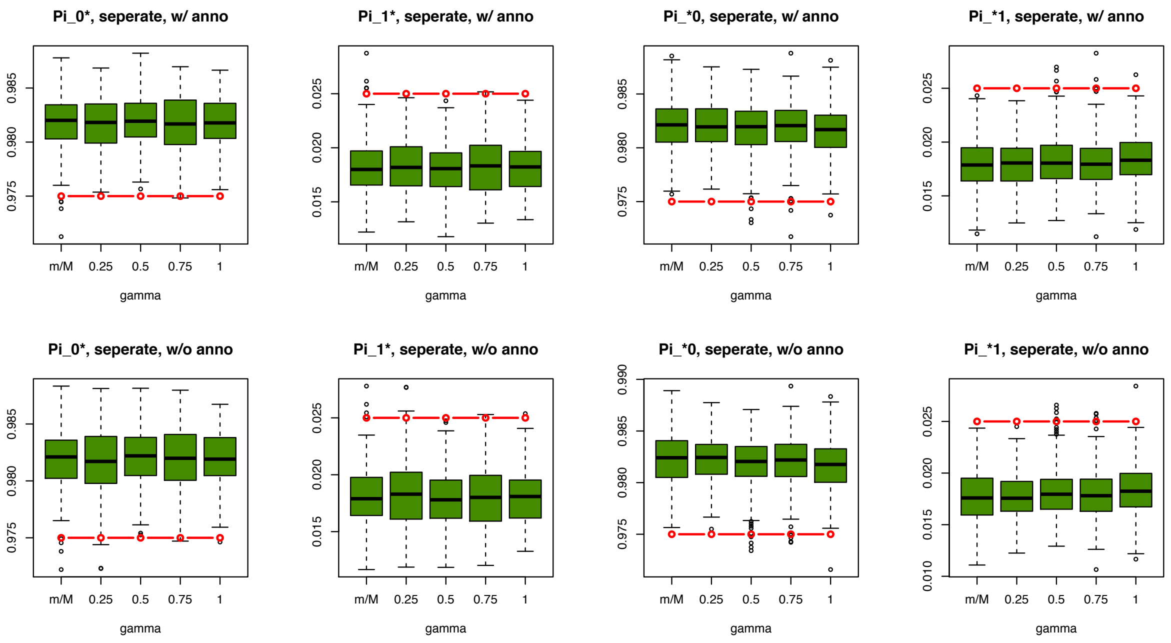

Figure S50: The estimated $\{\pi_l\}$ of separate analysis (single GWAS) at $N = 5000$, $m = 500$. The upper panel shows the estimated π_{0*}, π_{1*} and π_{*0}, π_{*1} with annotation, and the lower panel shows the estimated π_{0*}, π_{1*} and π_{*0}, π_{*1} without annotation. The red lines represent the true values.

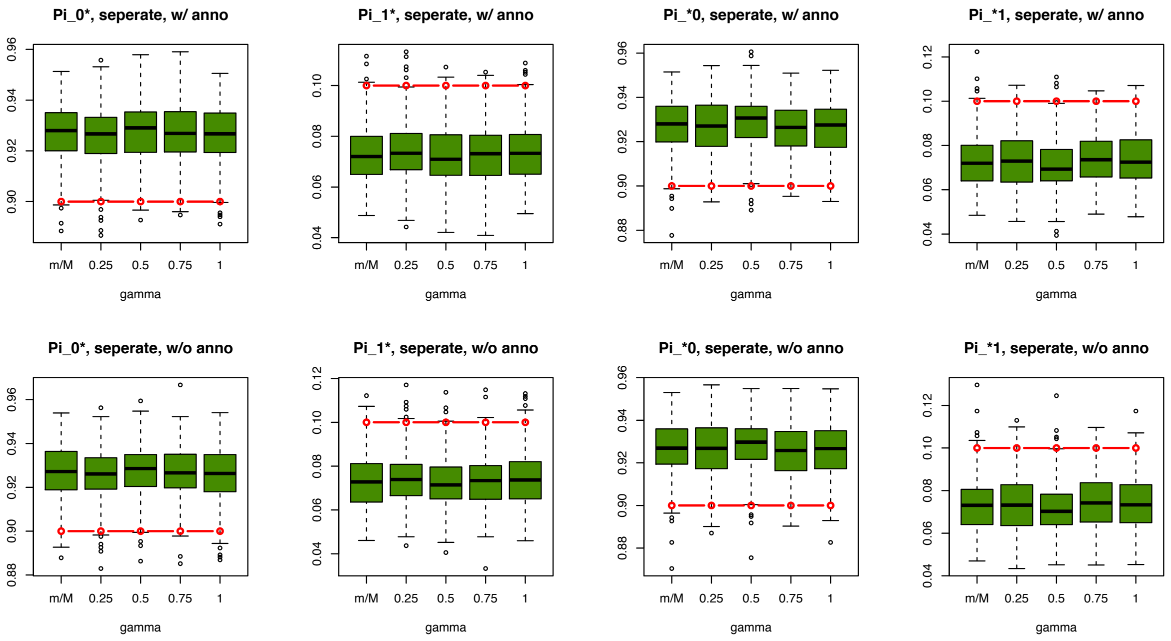

Figure S51: The estimated $\{\pi_l\}$ of separate analysis (single GWAS) at $N = 5000$, $m = 2000$. The upper panel shows the estimated π_{0*}, π_{1*} and π_{*0}, π_{*1} with annotation, and the lower panel shows the estimated π_{0*}, π_{1*} and π_{*0}, π_{*1} without annotation. The red lines represent the true values.

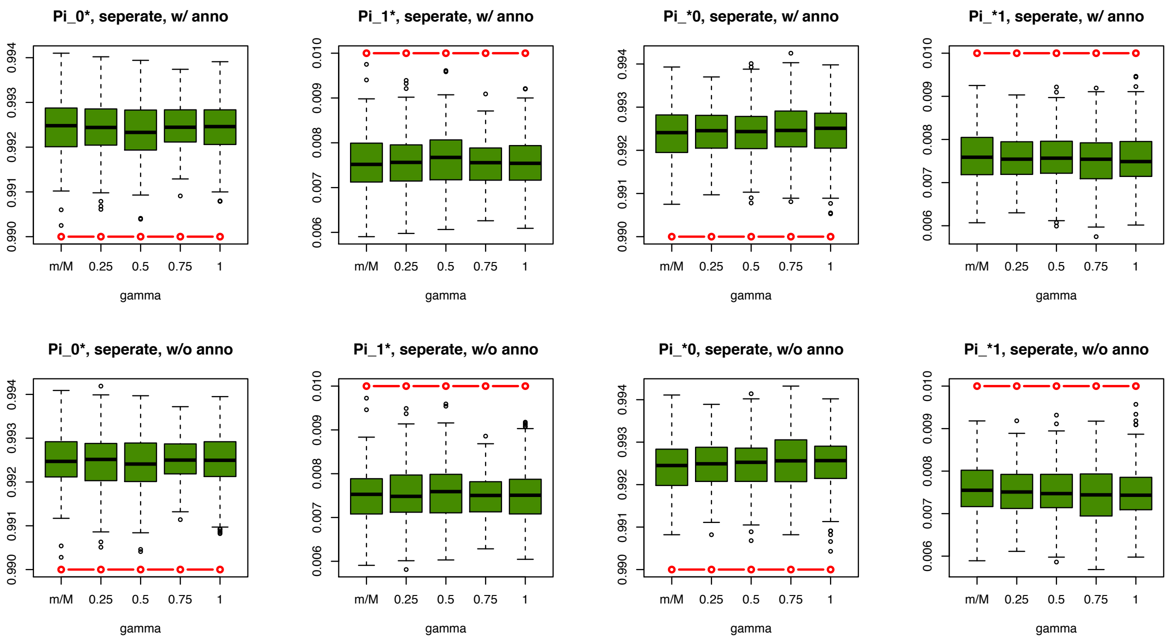

Figure S52: The estimated $\{\pi_l\}$ of separate analysis (single GWAS) at $N = 10000$, $m = 200$. The upper panel shows the estimated π_{0*}, π_{1*} and π_{*0}, π_{*1} with annotation, and the lower panel shows the estimated π_{0*}, π_{1*} and π_{*0}, π_{*1} without annotation. The red lines represent the true values.

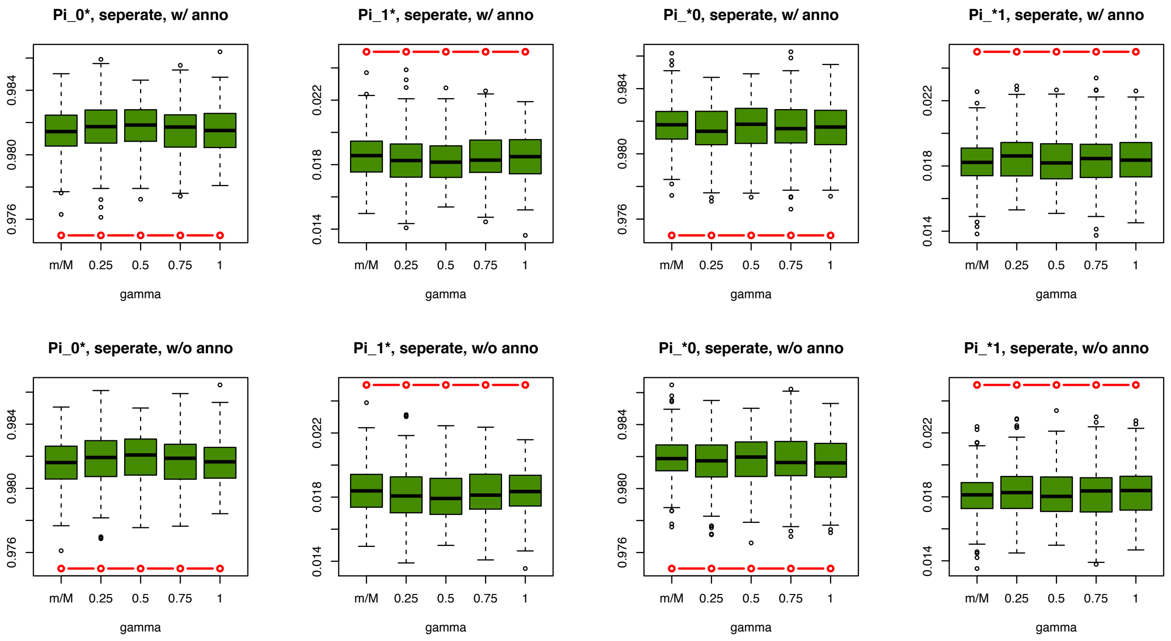

Figure S53: The estimated $\{\pi_l\}$ of separate analysis (single GWAS) at $N = 10000$, $m = 500$. The upper panel shows the estimated π_{0*}, π_{1*} and π_{*0}, π_{*1} with annotation, and the lower panel shows the estimated π_{0*}, π_{1*} and π_{*0}, π_{*1} without annotation. The red lines represent the true values.

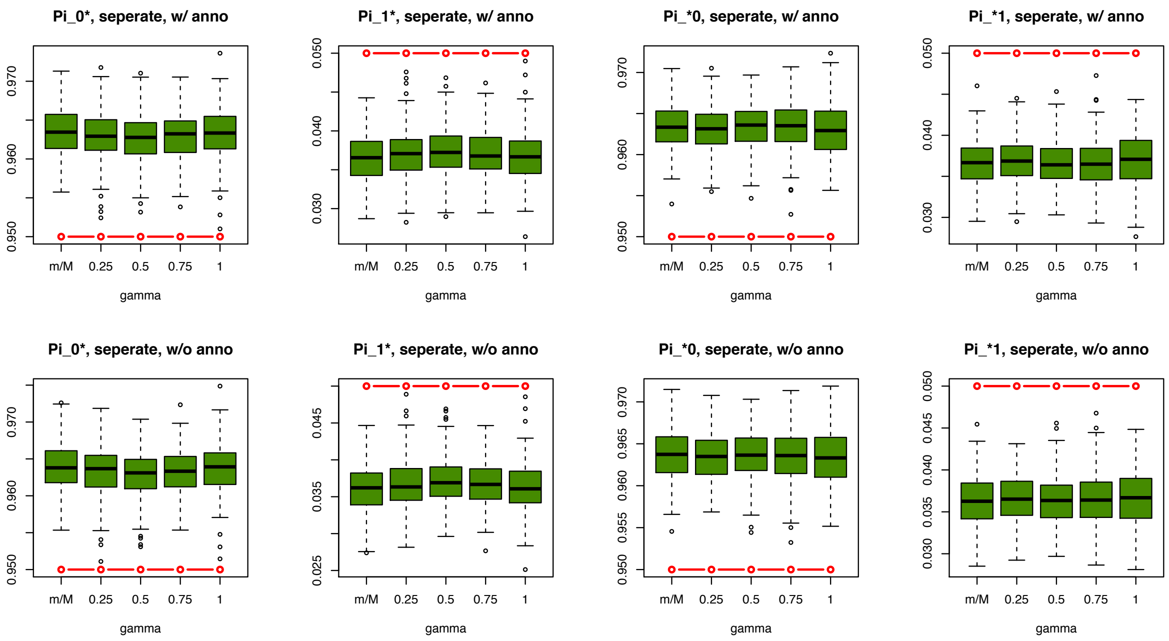

Figure S54: The estimated $\{\pi_l\}$ of separate analysis (single GWAS) at $N = 10000$, $m = 1000$. The upper panel shows the estimated π_{0*}, π_{1*} and π_{*0}, π_{*1} with annotation, and the lower panel shows the estimated π_{0*}, π_{1*} and π_{*0}, π_{*1} without annotation. The red lines represent the true values.

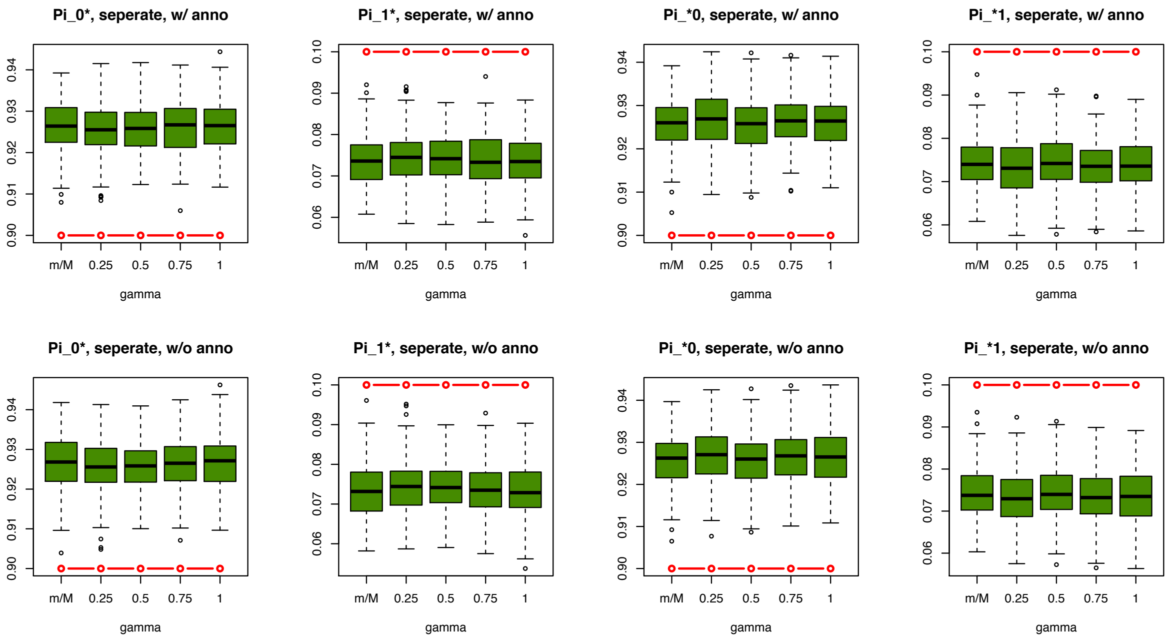

Figure S55: The estimated $\{\pi_l\}$ of separate analysis (single GWAS) at $N = 10000$, $m = 2000$. The upper panel shows the estimated π_{0*}, π_{1*} and π_{*0}, π_{*1} with annotation, and the lower panel shows the estimated π_{0*}, π_{1*} and π_{*0}, π_{*1} without annotation. The red lines represent the true values.

8.5 Parameter $\{\pi_l\}$ estimation in joint analysis (two GWAS) based on the random-effects model

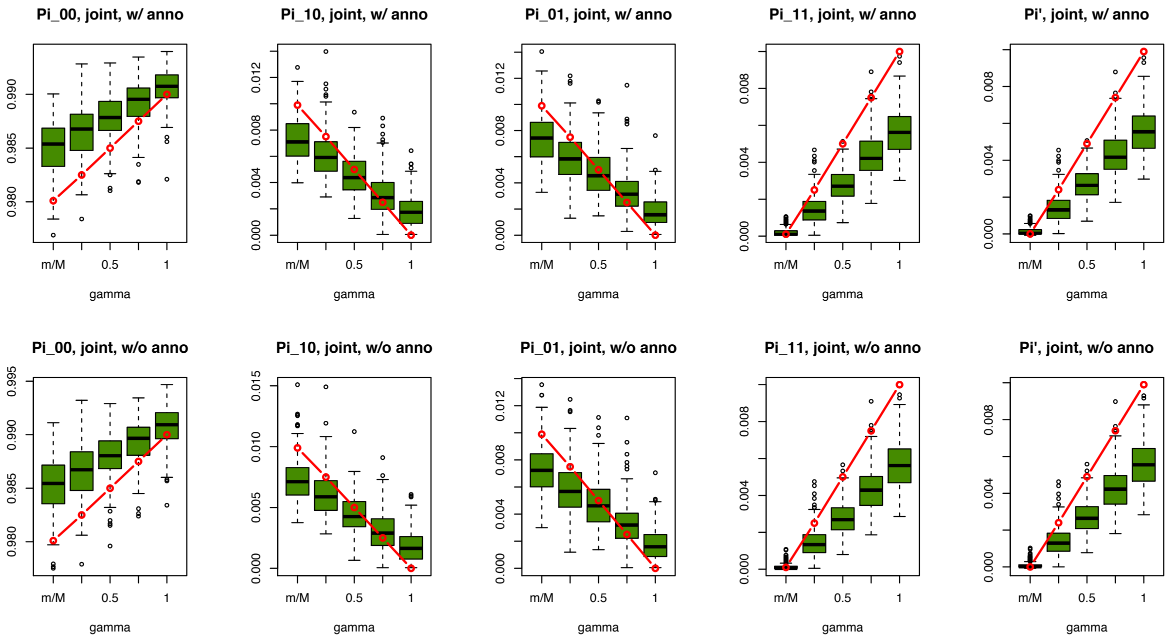

Figure S56: The estimated $\{\pi_l\}$ of joint analysis (two GWAS) at $N = 2000$, $m = 200$. The upper panel shows the estimated π_{00}, π_{10}, π_{01}, π_{11} with annotation, and the lower panel shows the estimated π_{00}, π_{10}, π_{01}, π_{11} without annotation. The last column $\pi' = \pi_{11} - (\pi_{01} + \pi_{11})(\pi_{10} + \pi_{11})$ indicates the level pleiotropy. The red lines represent the true values.

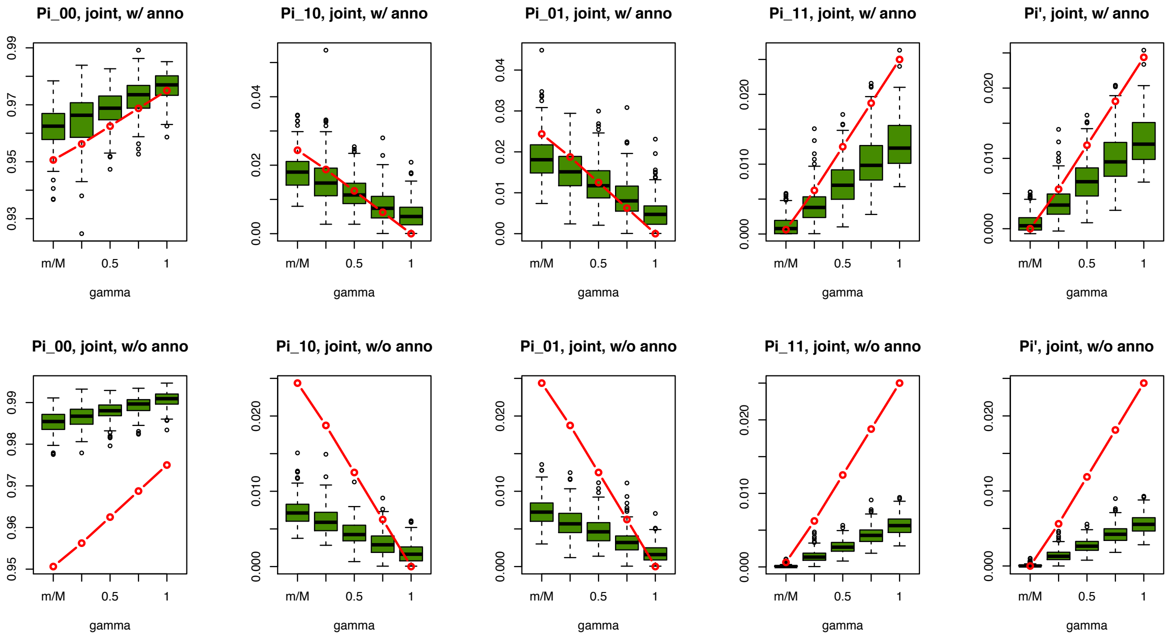

Figure S57: The estimated $\{\pi_l\}$ of joint analysis (two GWAS) at $N = 2000$, $m = 500$. The upper panel shows the estimated π_{00}, π_{10}, π_{01}, π_{11} with annotation, and the lower panel shows the estimated π_{00}, π_{10}, π_{01}, π_{11} without annotation. The last column $\pi' = \pi_{11} - (\pi_{01} + \pi_{11})(\pi_{10} + \pi_{11})$ indicates the level pleiotropy. The red lines represent the true values.

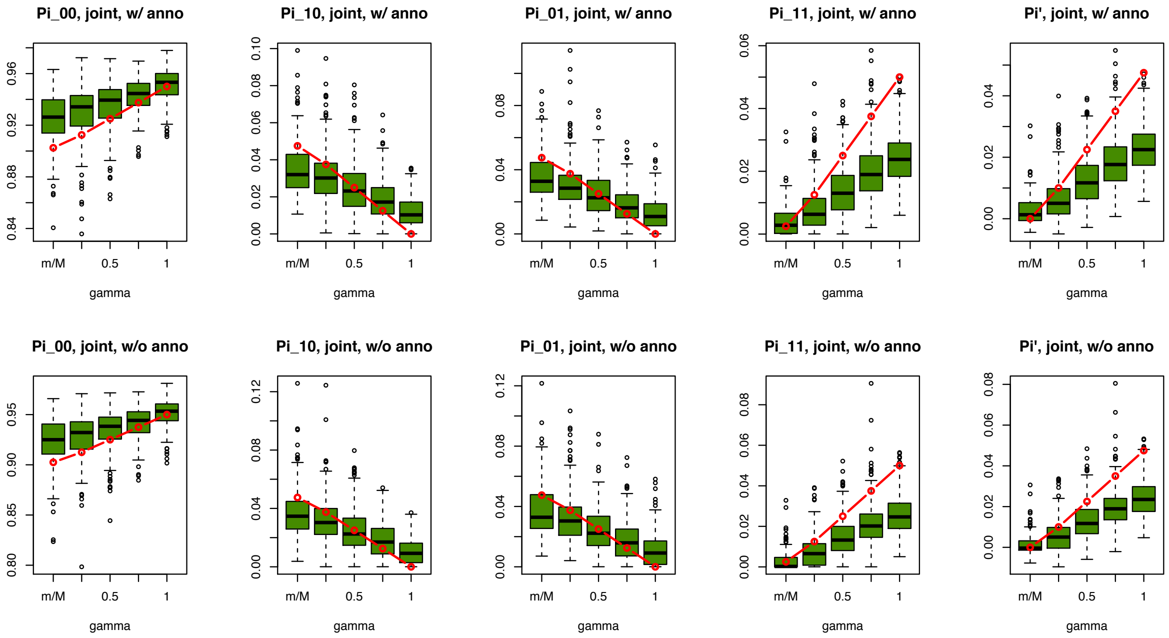

Figure S58: The estimated $\{\pi_l\}$ of joint analysis (two GWAS) at $N = 2000$, $m = 1000$. The upper panel shows the estimated π_{00}, π_{10}, π_{01}, π_{11} with annotation, and the lower panel shows the estimated π_{00}, π_{10}, π_{01}, π_{11} without annotation. The last column $\pi' = \pi_{11} - (\pi_{01} + \pi_{11})(\pi_{10} + \pi_{11})$ indicates the level pleiotropy. The red lines represent the true values.

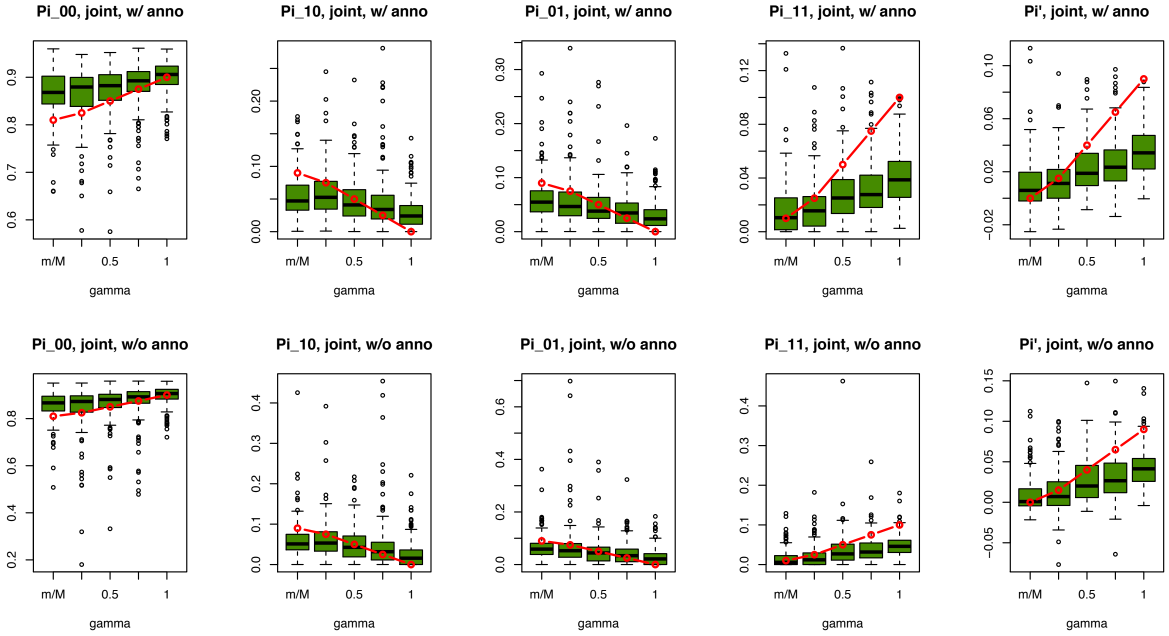

Figure S59: The estimated $\{\pi_l\}$ of joint analysis (two GWAS) at $N = 2000$, $m = 2000$. The upper panel shows the estimated π_{00}, π_{10}, π_{01}, π_{11} with annotation, and the lower panel shows the estimated π_{00}, π_{10}, π_{01}, π_{11} without annotation. The last column $\pi' = \pi_{11} - (\pi_{01} + \pi_{11})(\pi_{10} + \pi_{11})$ indicates the level pleiotropy. The red lines represent the true values.

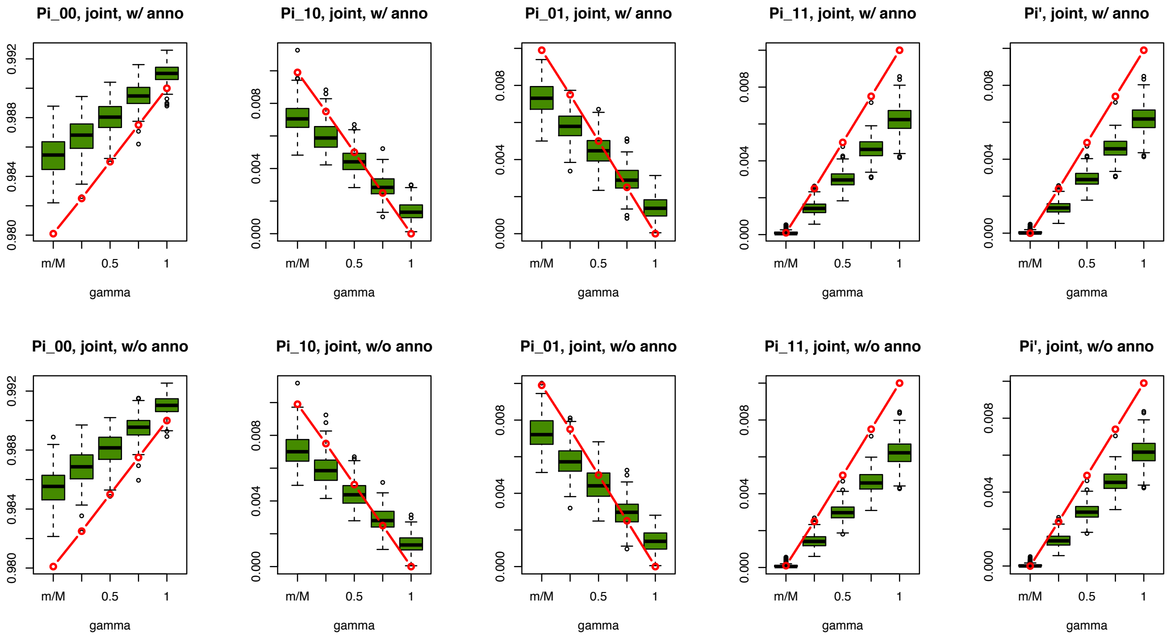

Figure S60: The estimated $\{\pi_l\}$ of joint analysis (two GWAS) at $N = 5000$, $m = 200$. The upper panel shows the estimated π_{00}, π_{10}, π_{01}, π_{11} with annotation, and the lower panel shows the estimated π_{00}, π_{10}, π_{01}, π_{11} without annotation. The last column $\pi' = \pi_{11} - (\pi_{01} + \pi_{11})(\pi_{10} + \pi_{11})$ indicates the level pleiotropy. The red lines represent the true values.

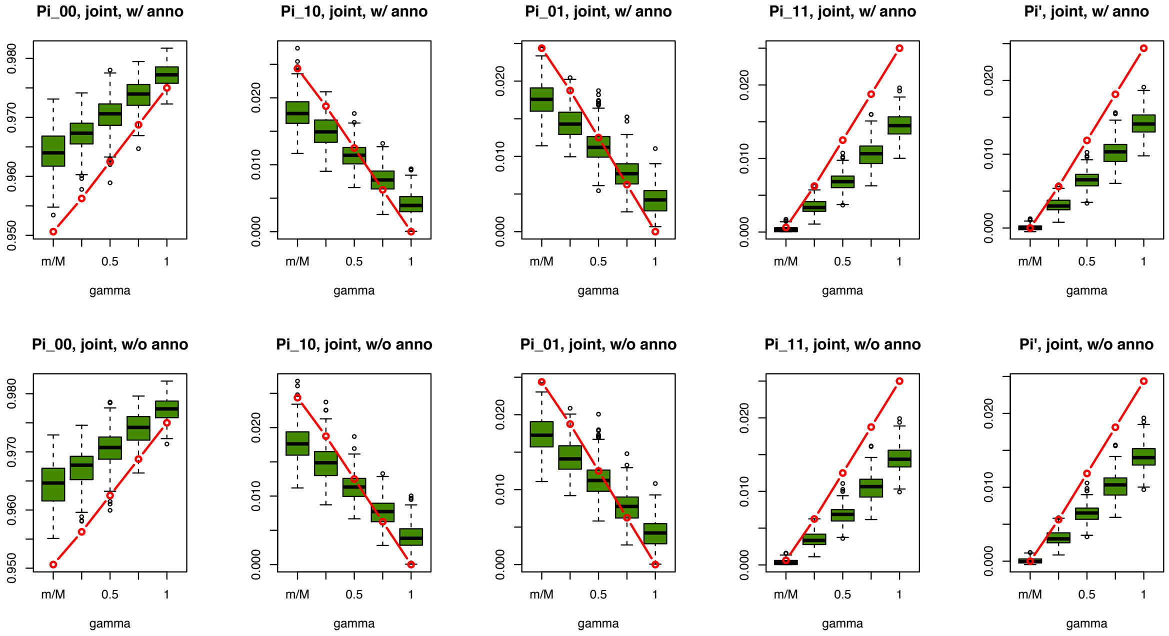

Figure S61: The estimated $\{\pi_l\}$ of joint analysis (two GWAS) at $N = 5000$, $m = 500$. The upper panel shows the estimated π_{00}, π_{10}, π_{01}, π_{11} with annotation, and the lower panel shows the estimated π_{00}, π_{10}, π_{01}, π_{11} without annotation. The last column $\pi' = \pi_{11} - (\pi_{01} + \pi_{11})(\pi_{10} + \pi_{11})$ indicates the level pleiotropy. The red lines represent the true values.

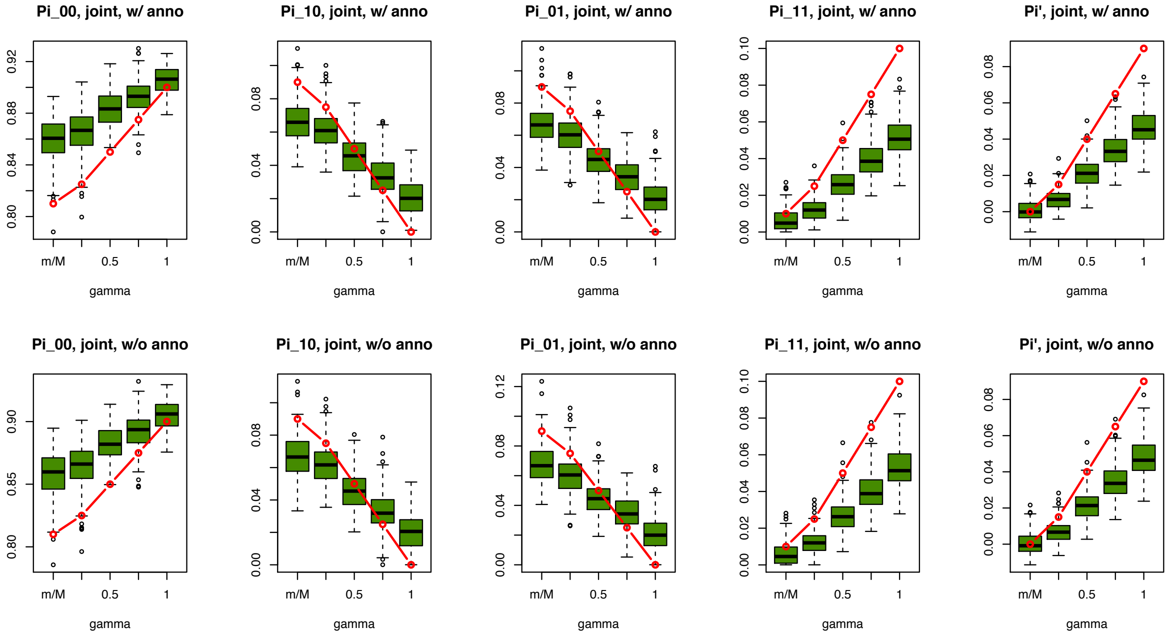

Figure S62: The estimated $\{\pi_l\}$ of joint analysis (two GWAS) at $N = 5000$, $m = 2000$. The upper panel shows the estimated π_{00}, π_{10}, π_{01}, π_{11} with annotation, and the lower panel shows the estimated π_{00}, π_{10}, π_{01}, π_{11} without annotation. The last column $\pi' = \pi_{11} - (\pi_{01} + \pi_{11})(\pi_{10} + \pi_{11})$ indicates the level pleiotropy. The red lines represent the true values.

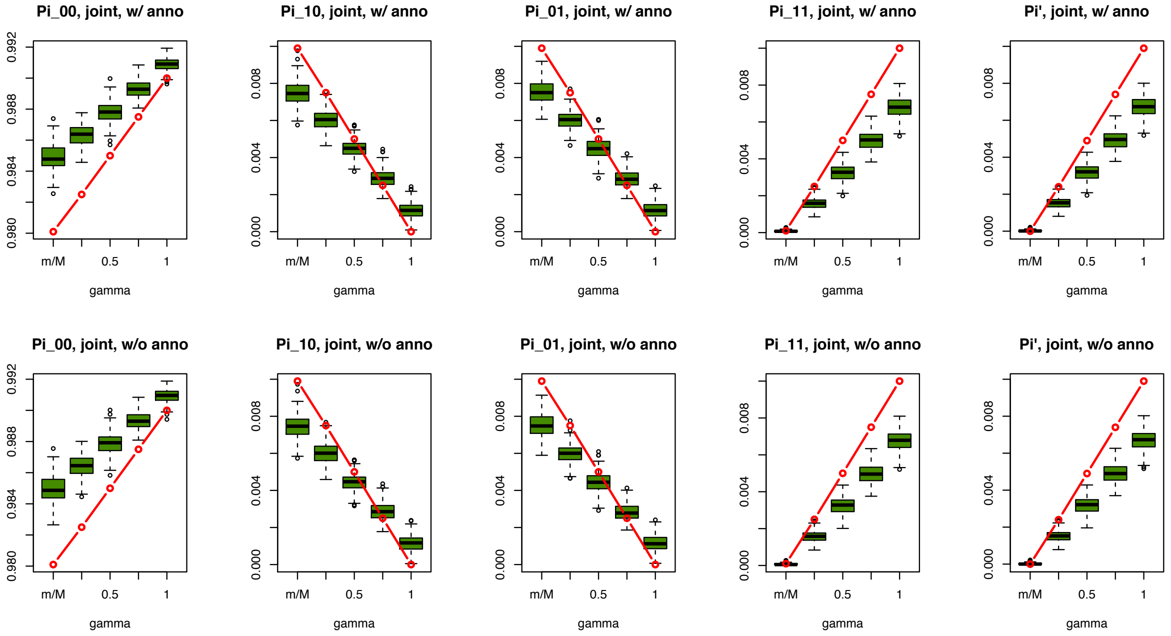

Figure S63: The estimated $\{\pi_l\}$ of joint analysis (two GWAS) at $N = 10000$, $m = 200$. The upper panel shows the estimated π_{00}, π_{10}, π_{01}, π_{11} with annotation, and the lower panel shows the estimated π_{00}, π_{10}, π_{01}, π_{11} without annotation. The last column $\pi' = \pi_{11} - (\pi_{01} + \pi_{11})(\pi_{10} + \pi_{11})$ indicates the level pleiotropy. The red lines represent the true values.

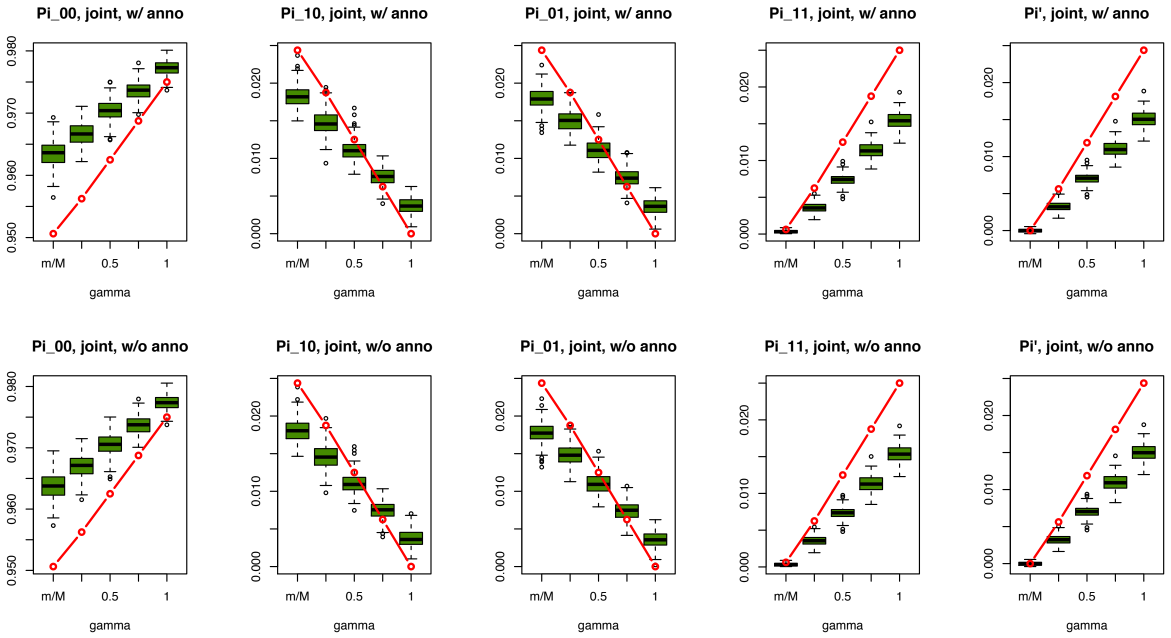

Figure S64: The estimated $\{\pi_l\}$ of joint analysis (two GWAS) at $N = 10000$, $m = 500$. The upper panel shows the estimated π_{00}, π_{10}, π_{01}, π_{11} with annotation, and the lower panel shows the estimated π_{00}, π_{10}, π_{01}, π_{11} without annotation. The last column $\pi' = \pi_{11} - (\pi_{01} + \pi_{11})(\pi_{10} + \pi_{11})$ indicates the level pleiotropy. The red lines represent the true values.

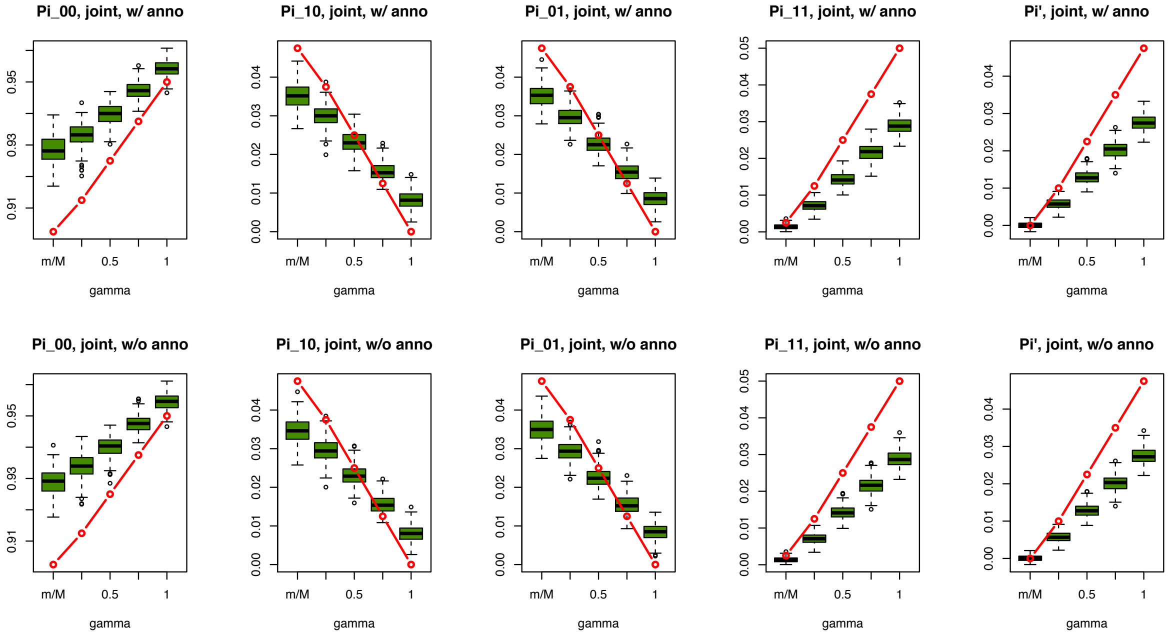

Figure S65: The estimated $\{\pi_l\}$ of joint analysis (two GWAS) at $N = 10000$, $m = 1000$. The upper panel shows the estimated π_{00}, π_{10}, π_{01}, π_{11} with annotation, and the lower panel shows the estimated π_{00}, π_{10}, π_{01}, π_{11} without annotation. The last column $\pi' = \pi_{11} - (\pi_{01} + \pi_{11})(\pi_{10} + \pi_{11})$ indicates the level pleiotropy. The red lines represent the true values.

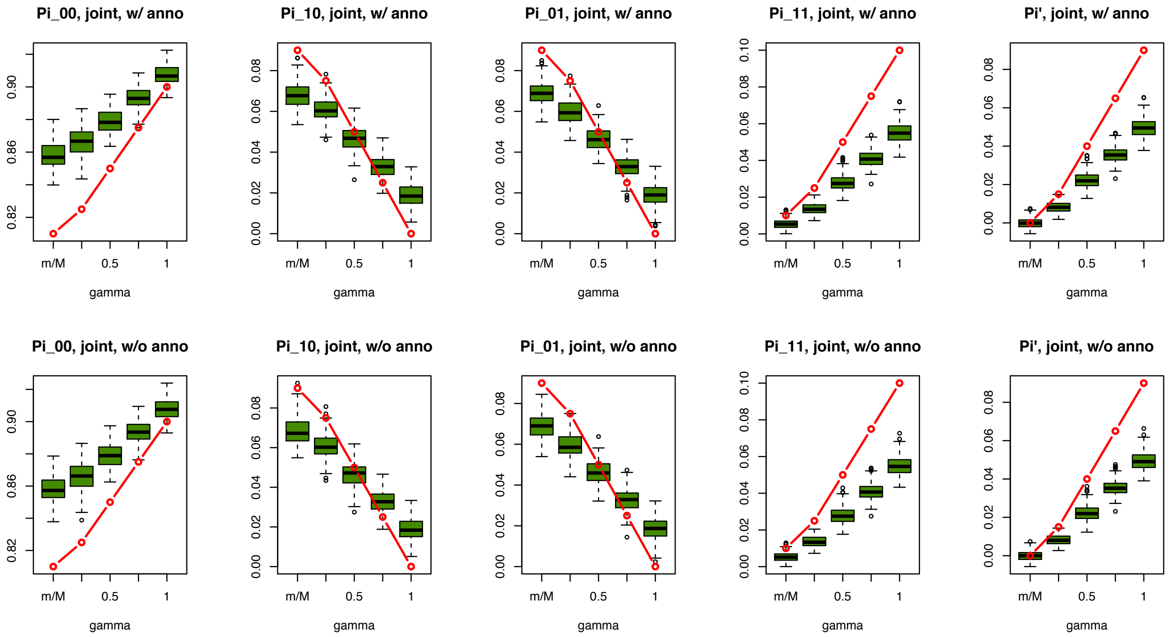

Figure S66: The estimated $\{\pi_l\}$ of joint analysis (two GWAS) at $N = 10000$, $m = 2000$. The upper panel shows the estimated π_{00}, π_{10}, π_{01}, π_{11} with annotation, and the lower panel shows the estimated π_{00}, π_{10}, π_{01}, π_{11} without annotation. The last column $\pi' = \pi_{11} - (\pi_{01} + \pi_{11})(\pi_{10} + \pi_{11})$ indicates the level pleiotropy. The red lines represent the true values.

8.6 Parameter $\{\pi_l\}$ estimation in separate analysis (single GWAS) based the GPA model

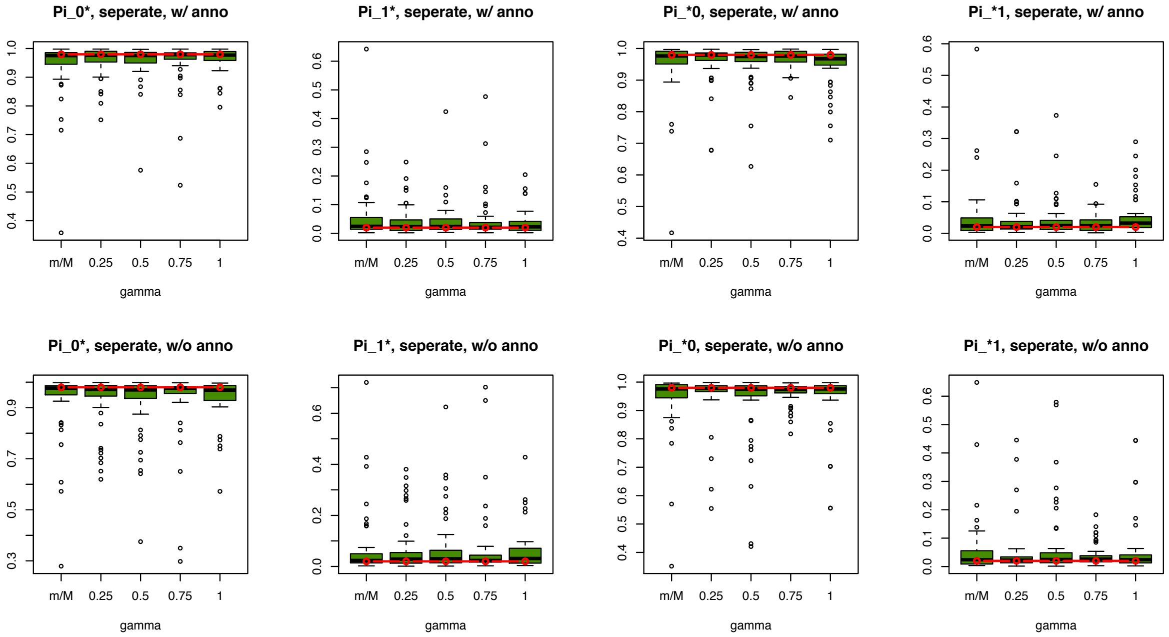

Figure S67: The estimated $\{\pi_l\}$ of separate analysis (single GWAS) at $M = 20000$, $m = 400$ with p-values of risk SNPs being simulated from Beta(0.6, 1). The upper panel shows the estimated π_{0*}, π_{1*} and π_{*0}, π_{*1} with annotation, and the lower panel shows the estimated π_{0*}, π_{1*} and π_{*0}, π_{*1} without annotation. The red lines represent the true values.

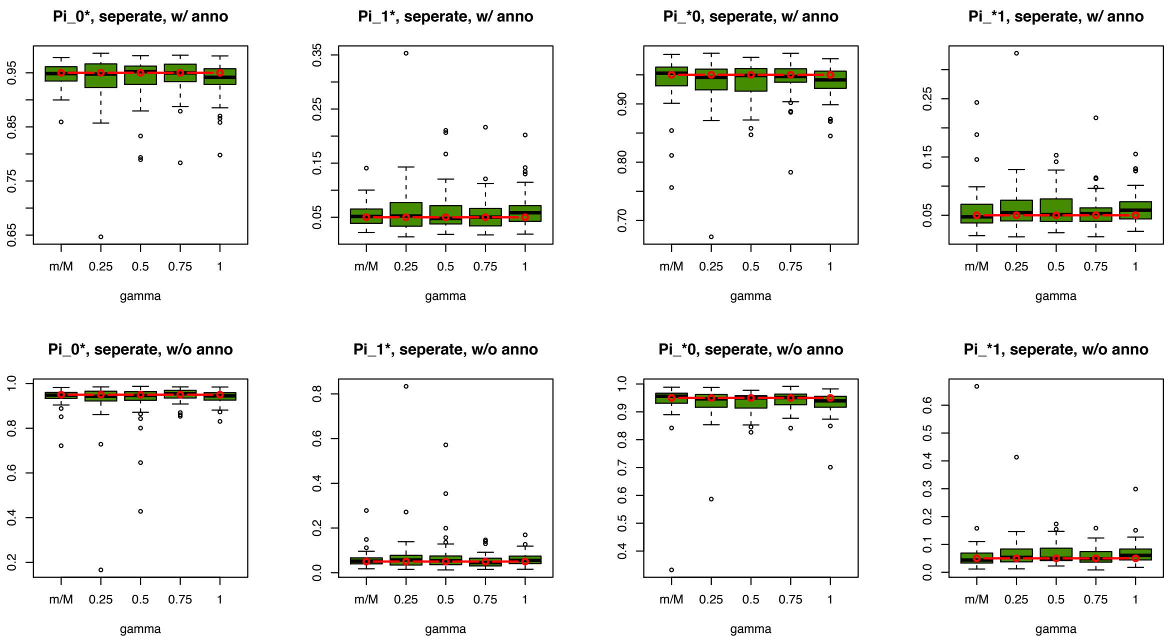

Figure S68: The estimated $\{\pi_l\}$ of separate analysis (single GWAS) at $M = 20000$, $m = 1000$ with p-values of risk SNPs being simulated from Beta(0.6, 1). The upper panel shows the estimated π_{0*}, π_{1*} and π_{*0}, π_{*1} with annotation, and the lower panel shows the estimated π_{0*}, π_{1*} and π_{*0}, π_{*1} without annotation. The red lines represent the true values.

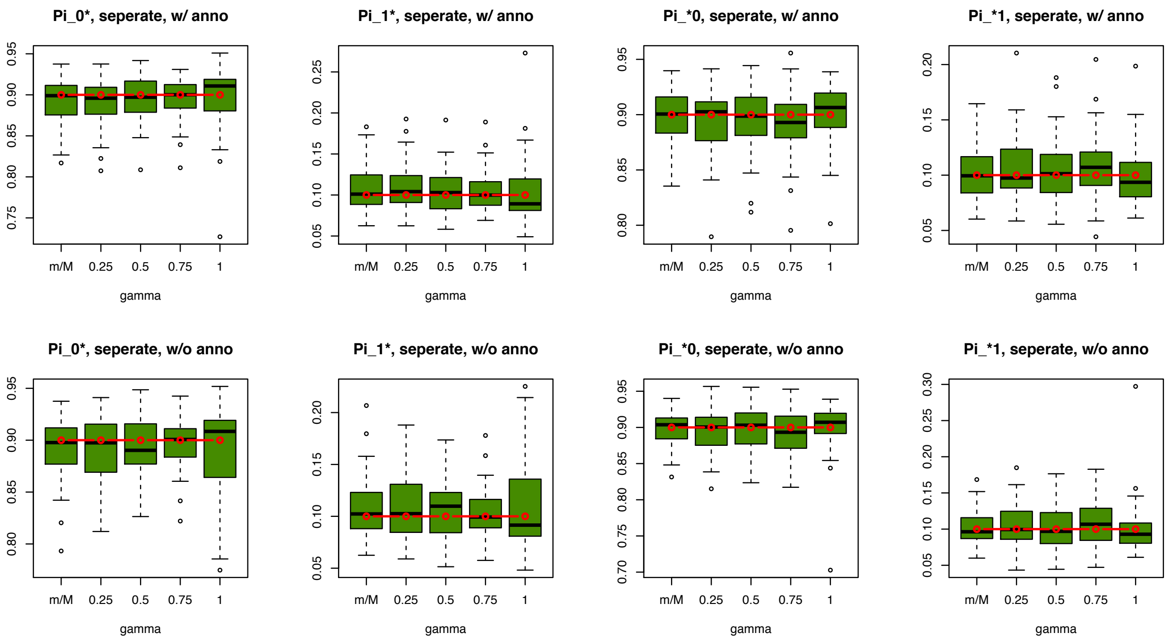

Figure S69: The estimated $\{\pi_l\}$ of separate analysis (single GWAS) at $M = 20000$, $m = 2000$ with p-values of risk SNPs being simulated from Beta(0.6, 1). The upper panel shows the estimated π_{0*}, π_{1*} and π_{*0}, π_{*1} with annotation, and the lower panel shows the estimated π_{0*}, π_{1*} and π_{*0}, π_{*1} without annotation. The red lines represent the true values.

8.7 Parameter $\{\pi_l\}$ estimation in joint analysis (two GWAS) based the GPA model

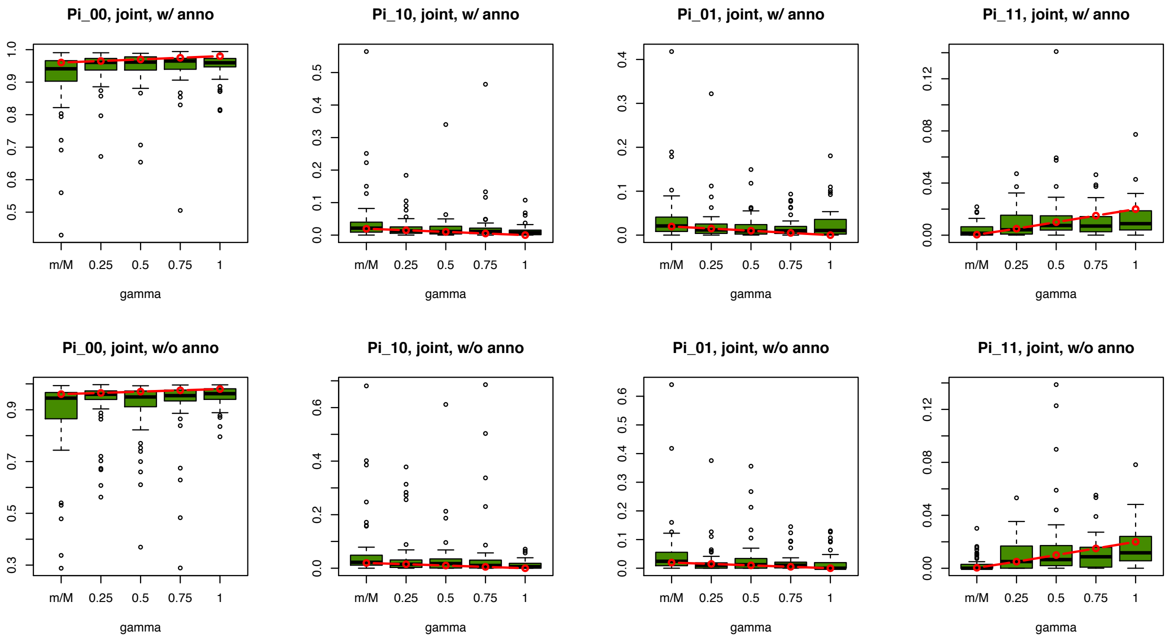

Figure S70: The estimated $\{\pi_l\}$ of joint analysis (two GWAS) at $M = 20000$, $m = 400$ with p values of risk SNPs being simulated from Beta (0.6, 1). The upper panel shows the estimated π_{00}, π_{10}, π_{01}, π_{11} with annotation, and the lower panel shows the estimated π_{00}, π_{10}, π_{01}, π_{11} without annotation. The red lines represent the true values.

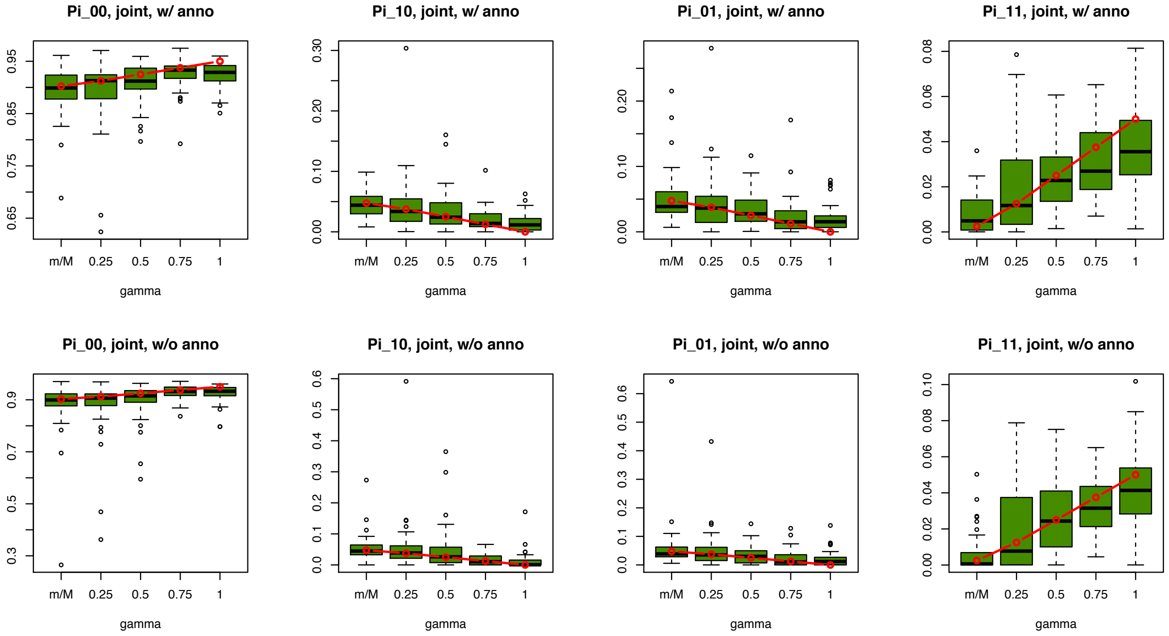

Figure S71: The estimated $\{\pi_l\}$ of joint analysis (two GWAS) at $M = 20000$, $m = 1000$ with p values of risk SNPs being simulated from Beta (0.6, 1). The upper panel shows the estimated π_{00}, π_{10}, π_{01}, π_{11} with annotation, and the lower panel shows the estimated π_{00}, π_{10}, π_{01}, π_{11} without annotation. The red lines represent the true values.

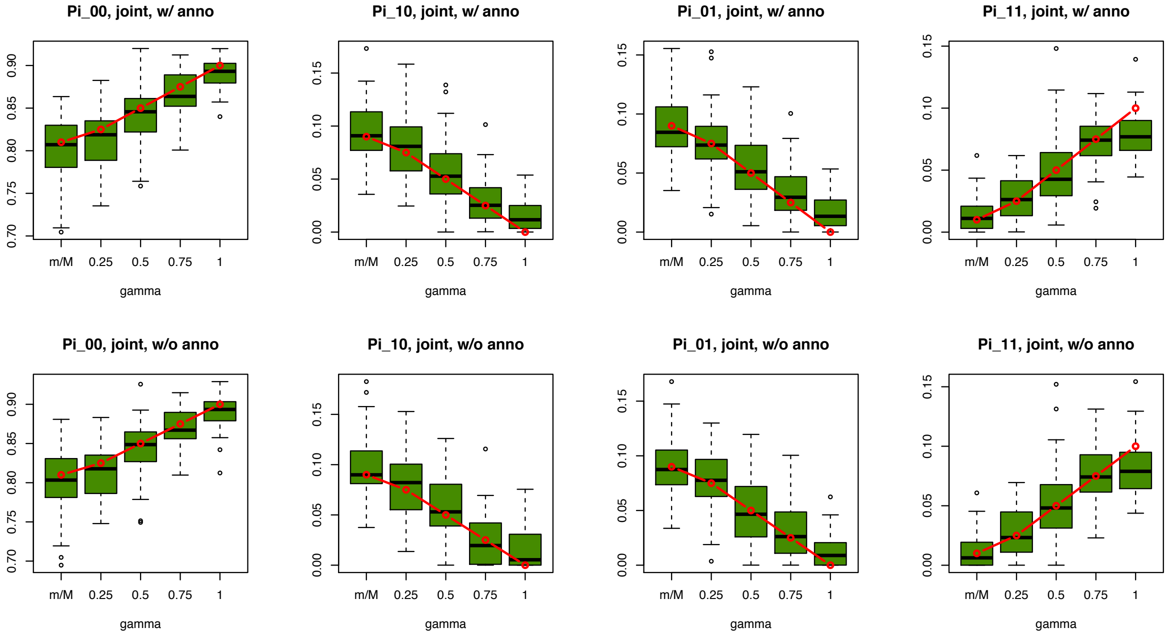

Figure S72: The estimated $\{\pi_l\}$ of joint analysis (two GWAS) at $M = 20000$, $m = 2000$ with p values of risk SNPs being simulated from Beta (0.6, 1). The upper panel shows the estimated π_{00}, π_{10}, π_{01}, π_{11} with annotation, and the lower panel shows the estimated π_{00}, π_{10}, π_{01}, π_{11} without annotation. The red lines represent the true values.

8.8 Performance evaluation with moderate heritability and pleiotropy

We also performed some additional simulations with h^2 being much more moderate ($h^2 = 0.3$). Results for $m = 500$, $N = 2000, 5000, 10000$ are shown in Figures S73-S75. The results demonstrate that, at moderate h^2 levels, GPA can still effectively improve the power by leveraging the pleiotropy between related traits.

We have also noticed that the power improvement could be a function of the sample size. When the sample size is relatively large, the improvement at weak pleiotropy level becomes more visible. To demonstrate this, we performed additional simulations at even weaker pleiotropy level ($\gamma = 0.1, 0.12, 0.15, 0.2$) and even larger sample sizes ($N = 20000, 30000, 40000, 50000$). The results are shown in Figure S76. We can see that GPA will be able to better leverage the pleiotropy between moderately related traits with the increased sample size of GWAS.

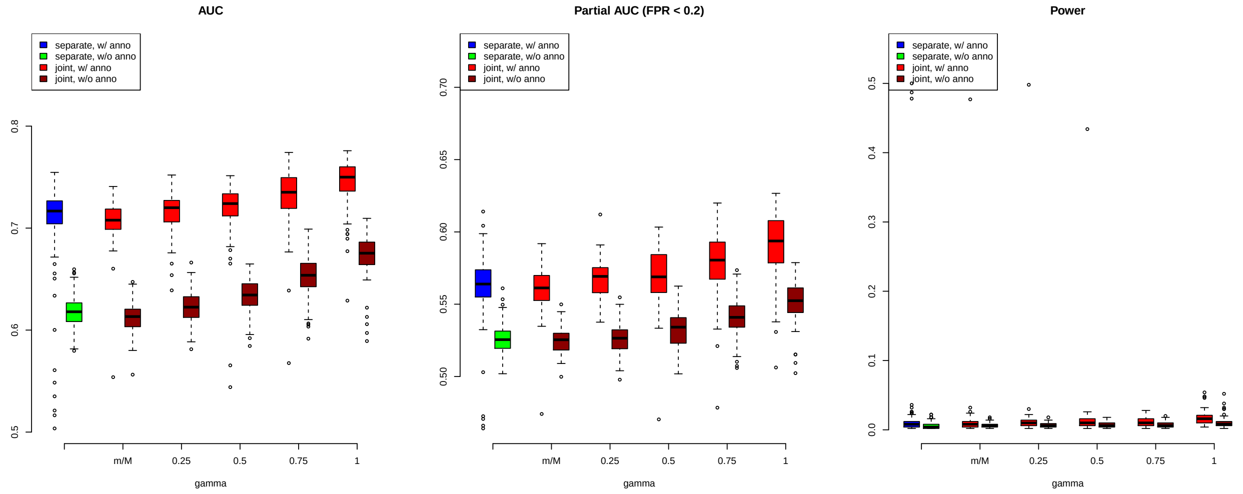

Figure S73: The performance of GPA at moderate heritability level ($h^2 = 0.3$, $m = 500$ and $N = 2000$). Here γ is the proportion of shared risk SNPs between the two GWAS.

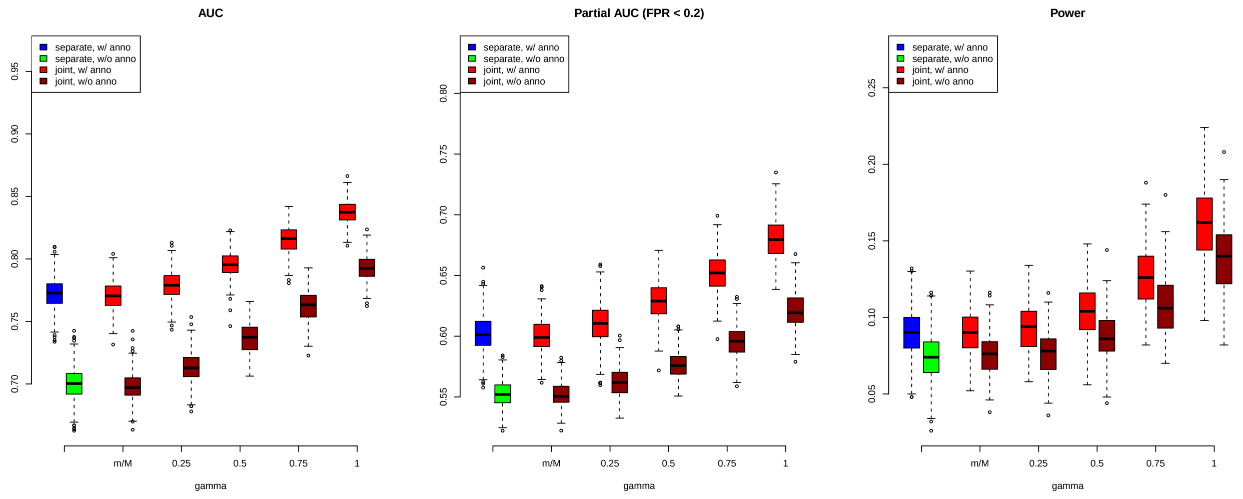

Figure S74: The performance of GPA at moderate heritability level ($h^2 = 0.3$, $m = 500$ and $N = 5000$). Here γ is the proportion of shared risk SNPs between the two GWAS.

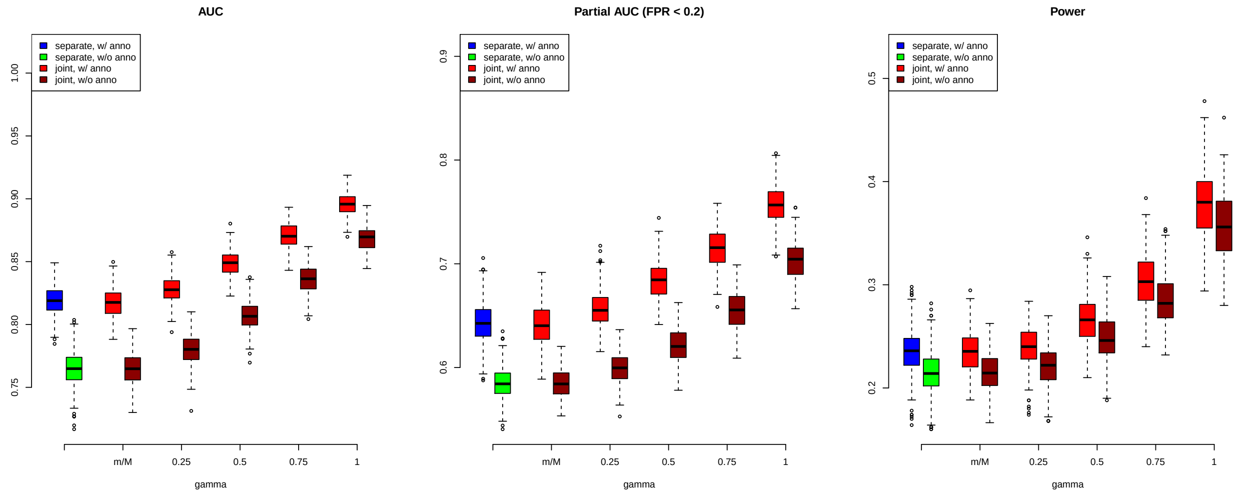

Figure S75: The performance of GPA at moderate heritability level ($h^2 = 0.3$, $m = 500$ and $N = 10000$). Here γ is the proportion of shared risk SNPs between the two GWAS.

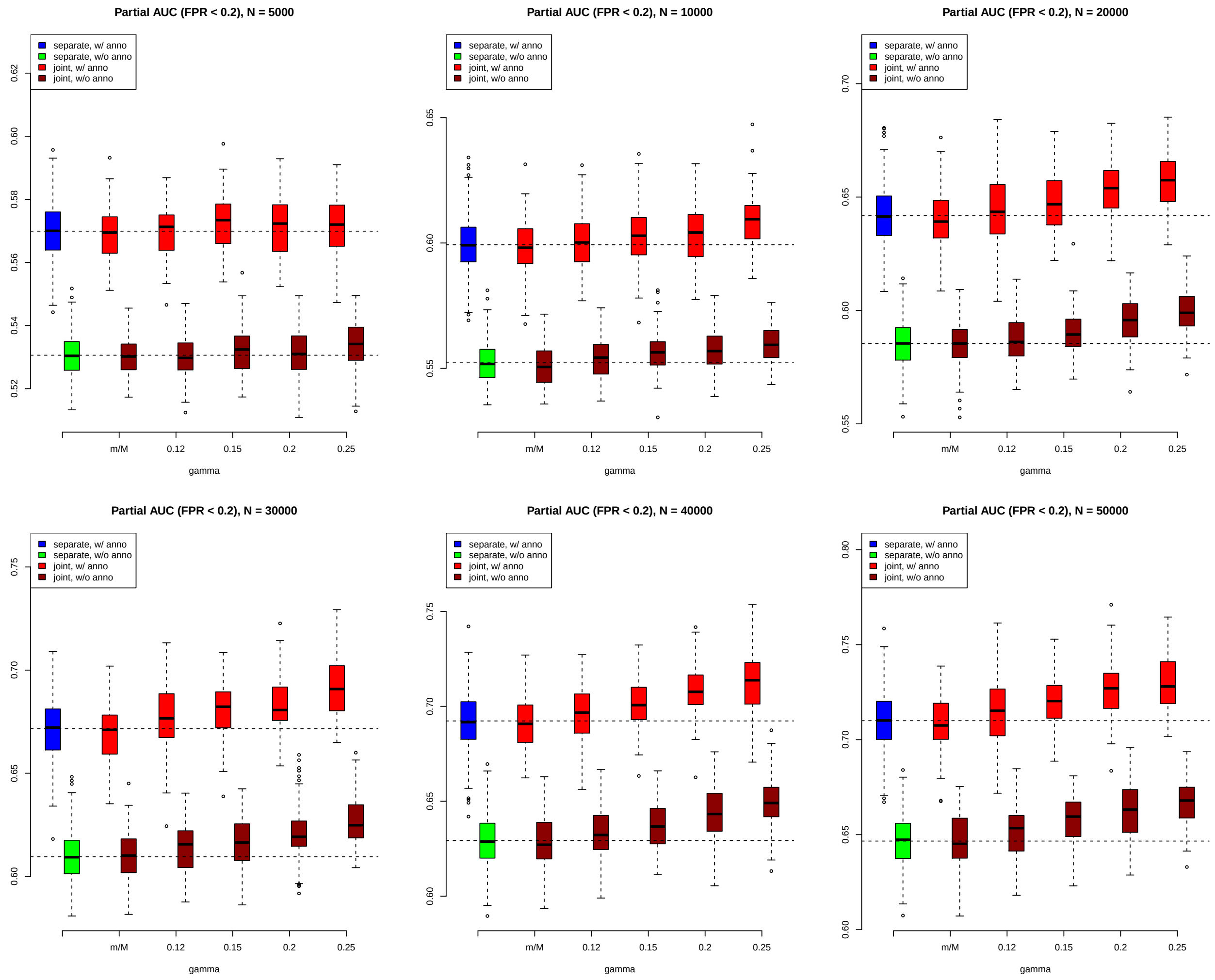

Figure S76: The performance of GPA at different sample sizes with weak pleiotropy ($h^2 = 0.3$ and $m = 500$). Here γ is the proportion of shared risk SNPs between the two GWAS.

8.9 Performance comparison between GPA and conditional FDR

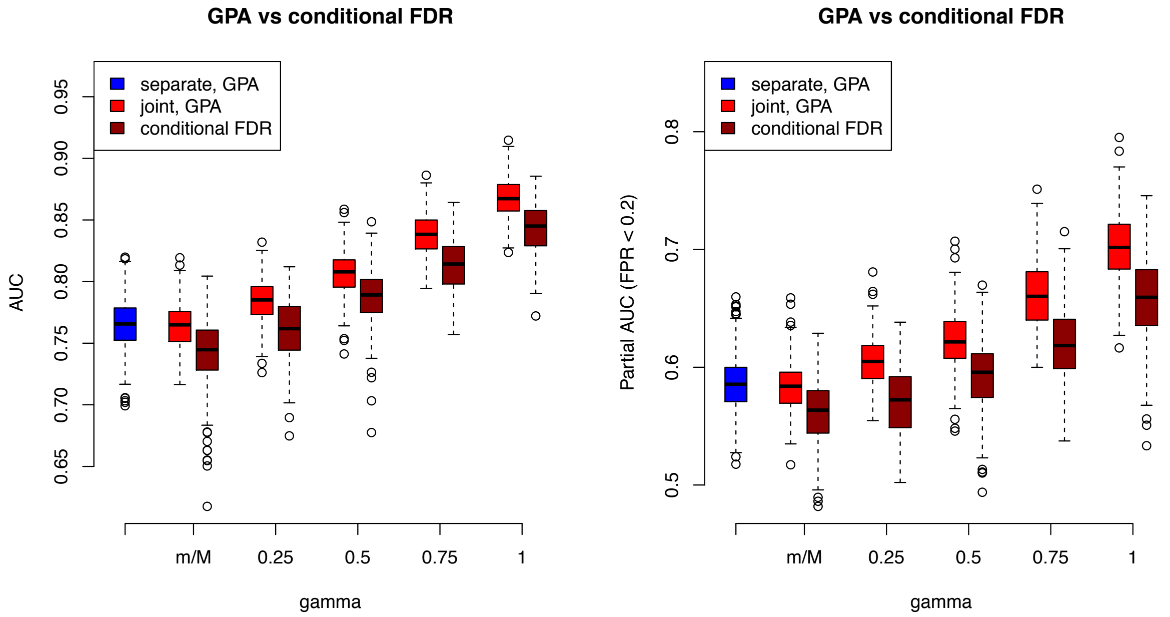

Figure S77: The comparison of AUC and partial AUC between GPA and the conditional FDR approach at $N = 2000$, $m = 200$.

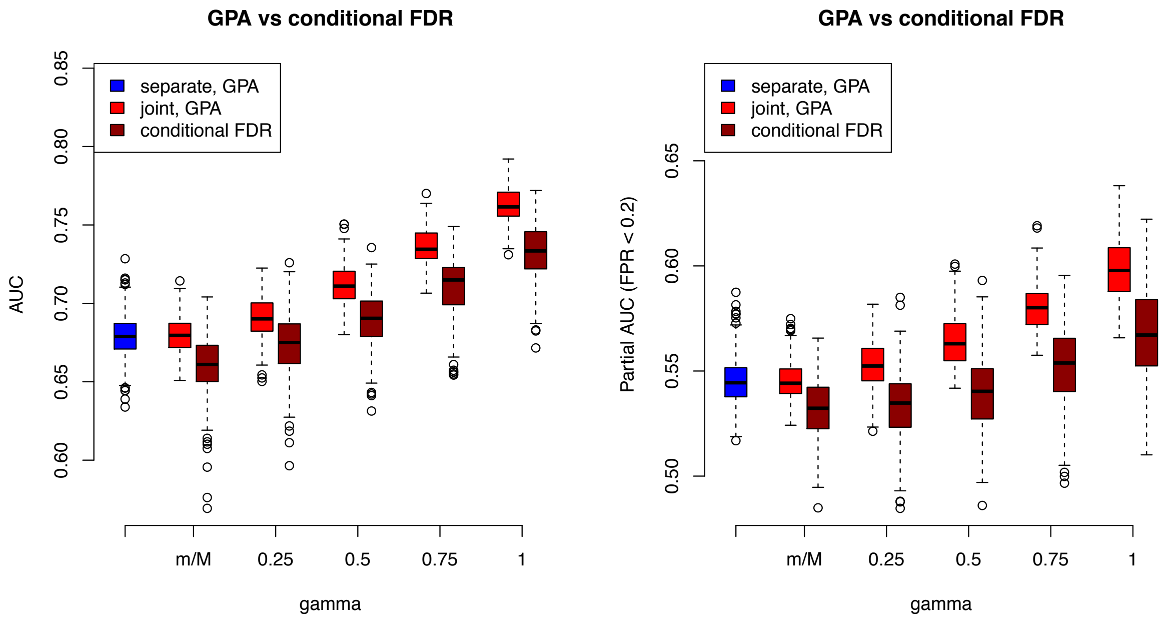

Figure S78: The comparison of AUC and partial AUC between GPA and the conditional FDR approach at $N = 2000$, $m = 500$.

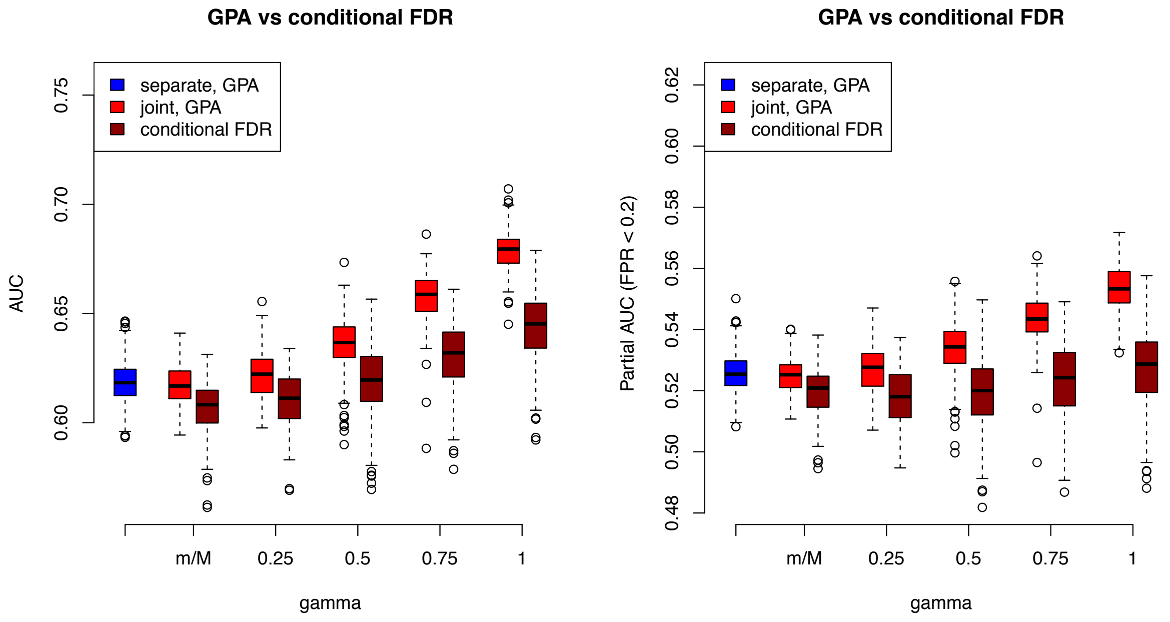

Figure S79: The comparison of AUC and partial AUC between GPA and the conditional FDR approach at $N = 2000$, $m = 1000$.

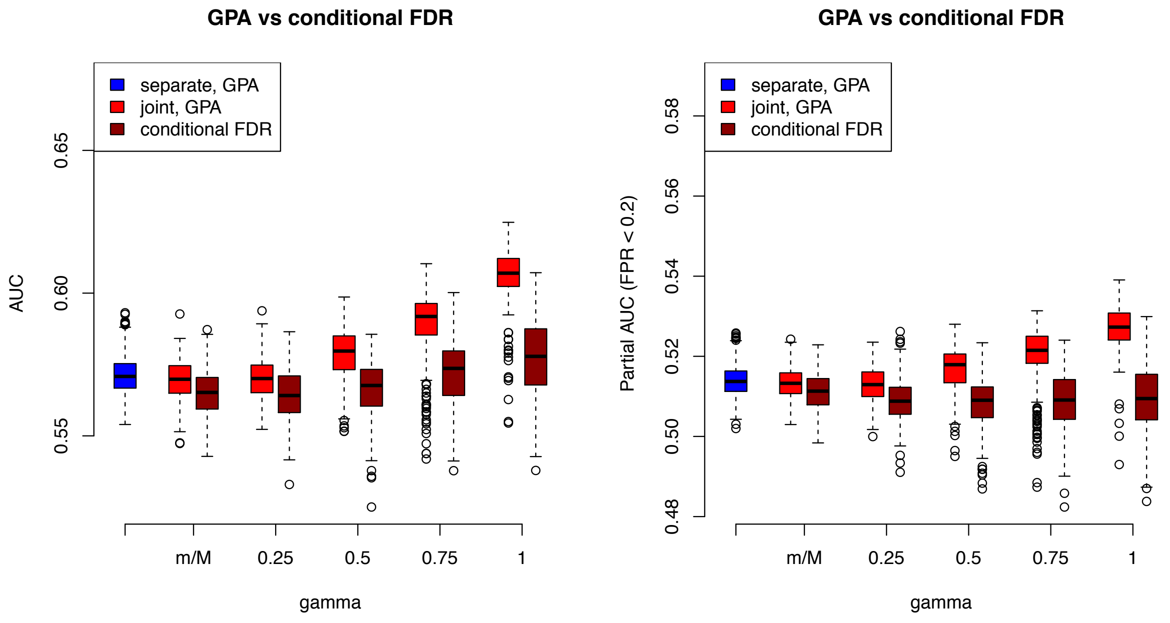

Figure S80: The comparison of AUC and partial AUC between GPA and the conditional FDR approach at $N = 2000$, $m = 2000$.

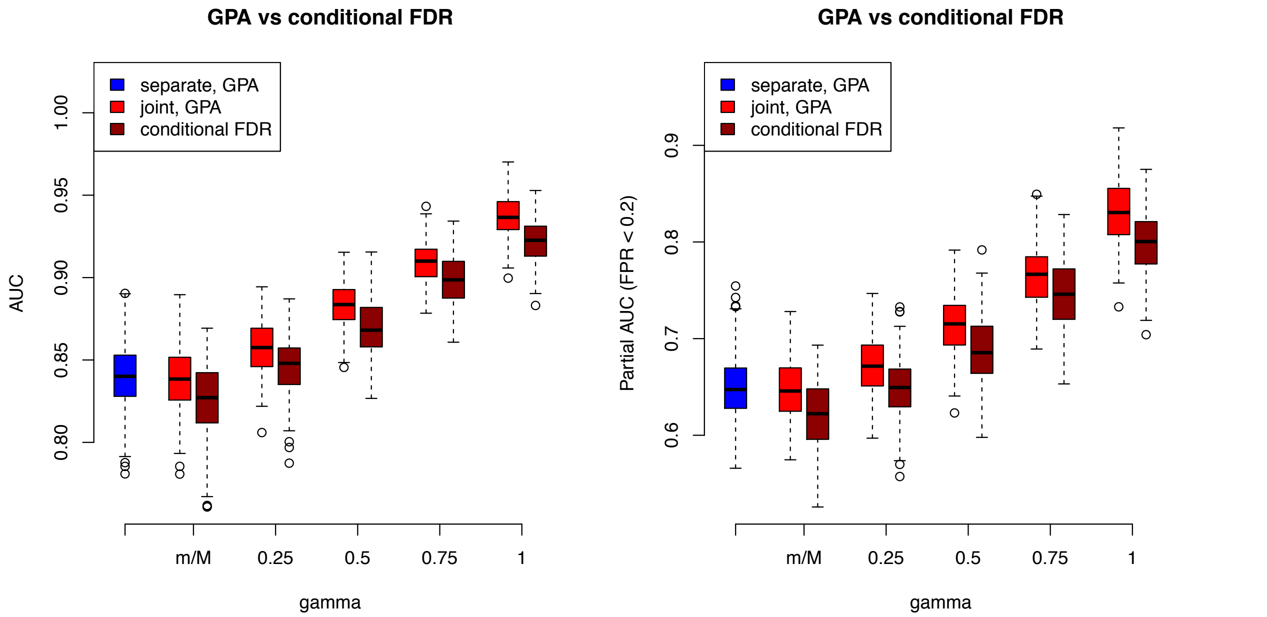

Figure S81: The comparison of AUC and partial AUC between GPA and the conditional FDR approach at $N = 5000$, $m = 200$.

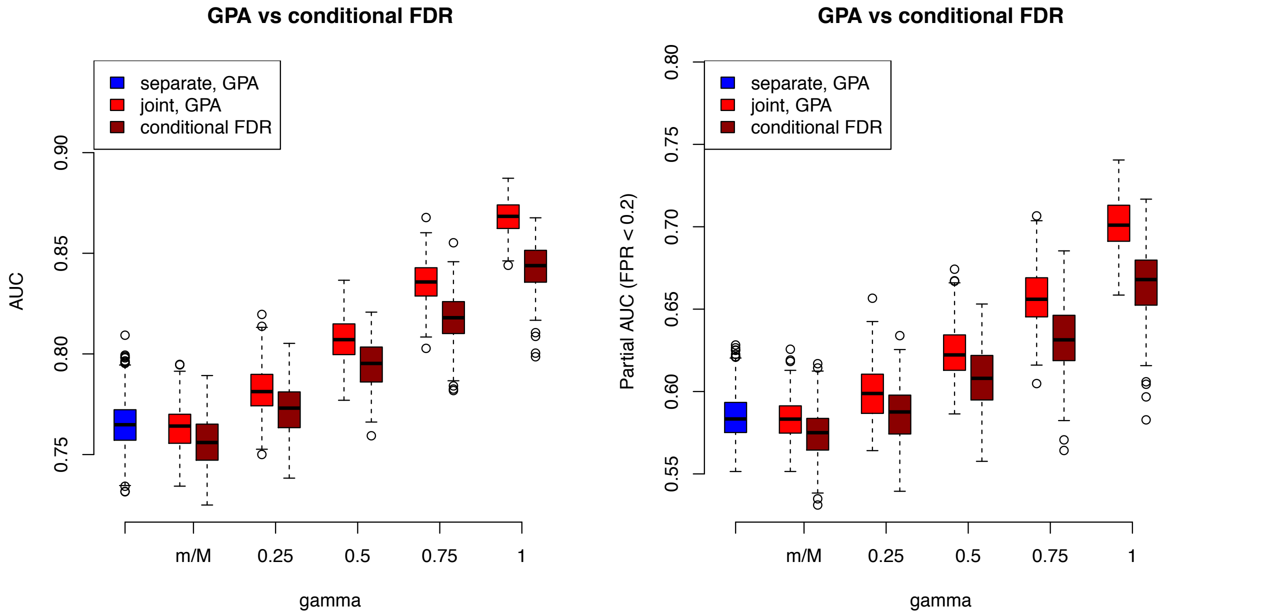

Figure S82: The comparison of AUC and partial AUC between GPA and the conditional FDR approach at $N = 5000$, $m = 500$.

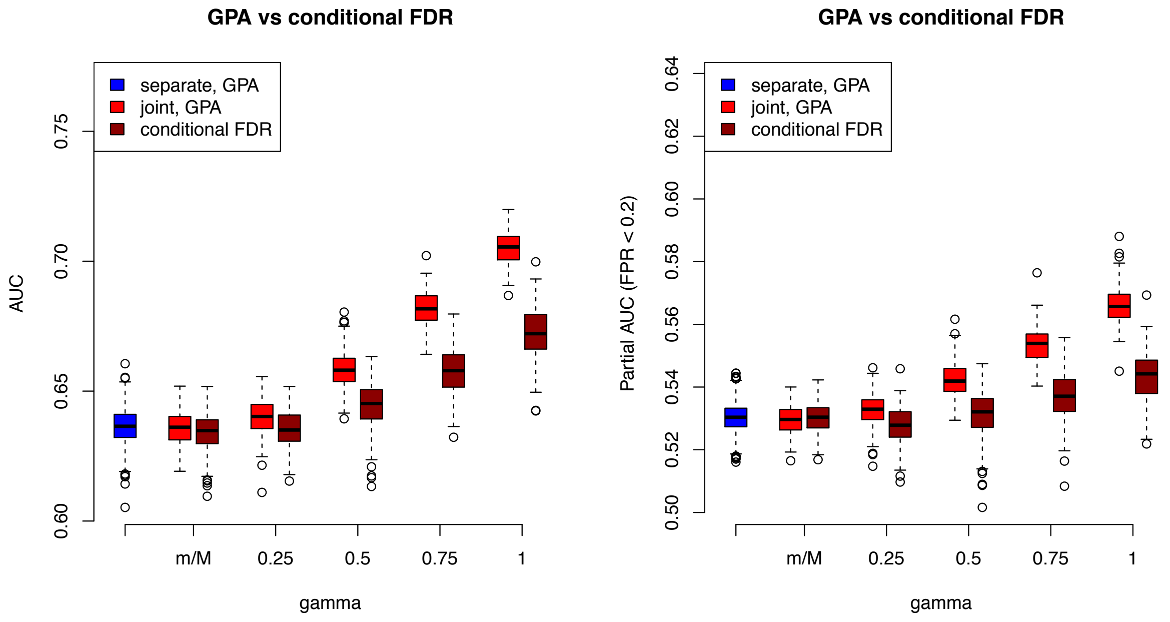

Figure S83: The comparison of AUC and partial AUC between GPA and the conditional FDR approach at $N = 5000$, $m = 1000$.

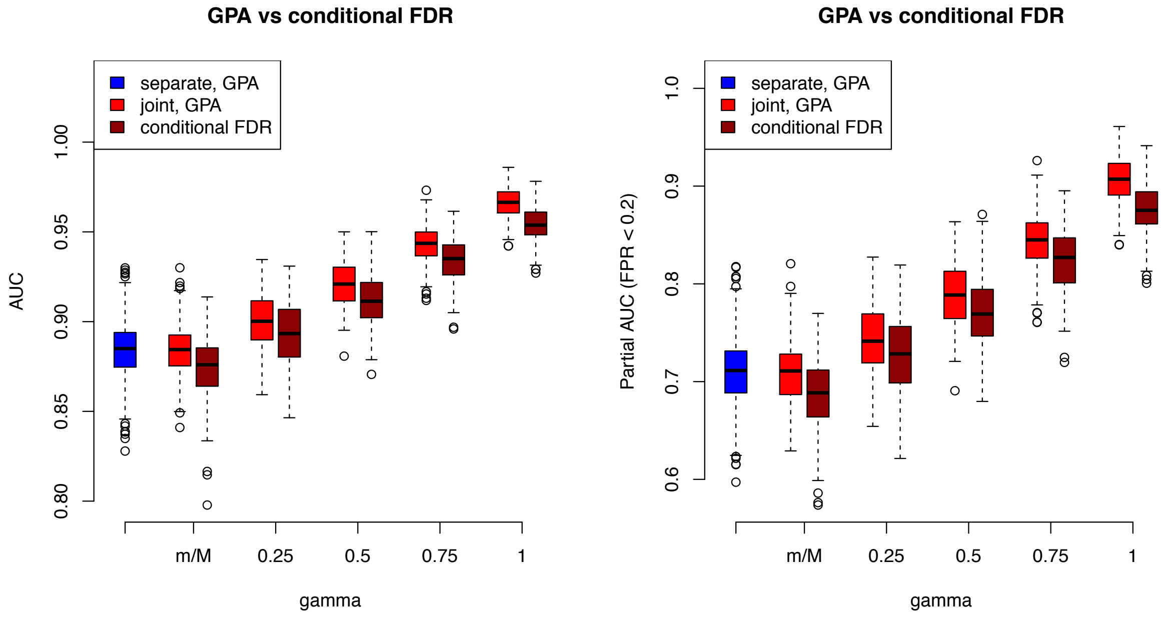

Figure S84: The comparison of AUC and partial AUC between GPA and the conditional FDR approach at $N = 5000$, $m = 2000$.

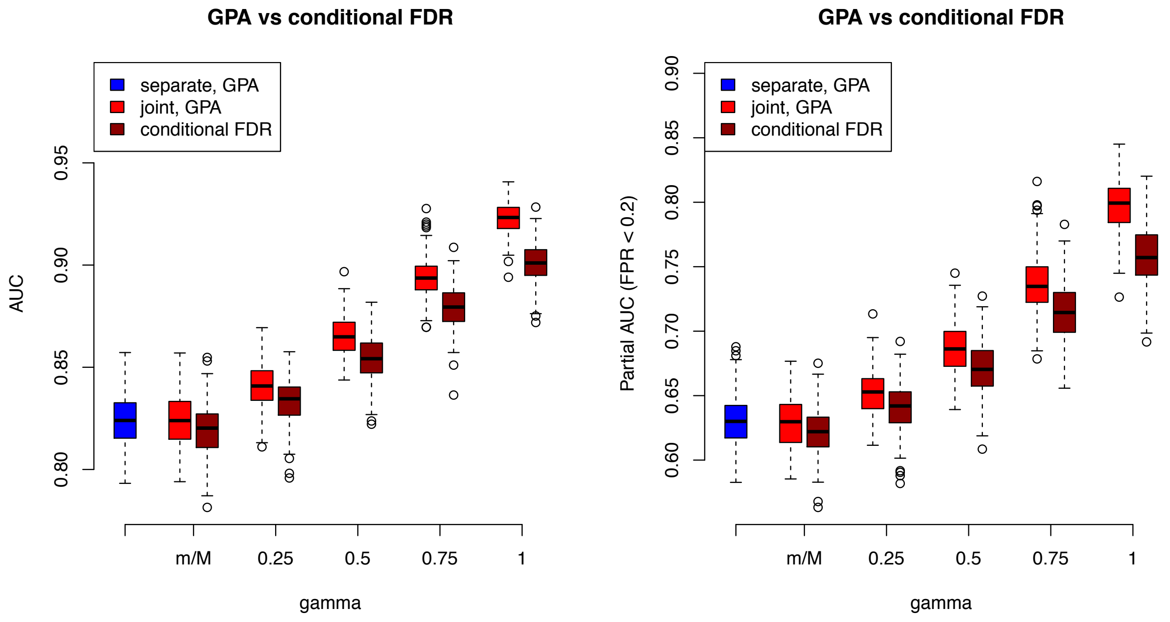

Figure S85: The comparison of AUC and partial AUC between GPA and the conditional FDR approach at $N = 10000$, $m = 200$.

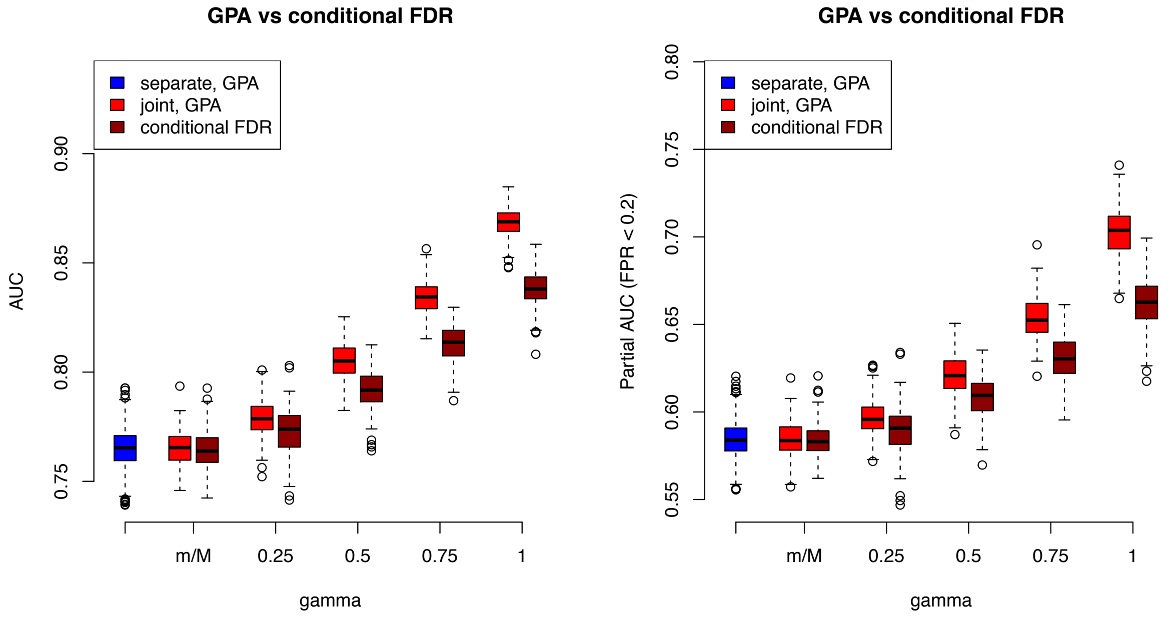

Figure S86: The comparison of AUC and partial AUC between GPA and the conditional FDR approach at $N = 10000$, $m = 1000$.

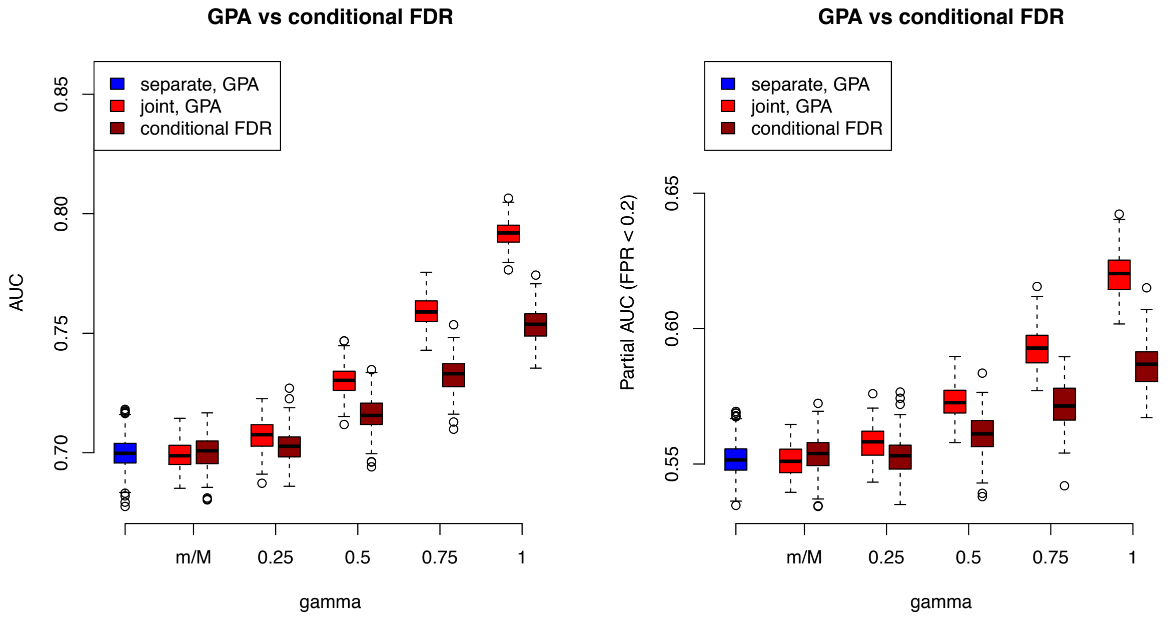

Figure S87: The comparison of AUC and partial AUC between GPA and the conditional FDR approach at $N = 10000$, $m = 2000$.

9 Goodness of fit between GPA and real data sets from psychiatric Genomics Consortium

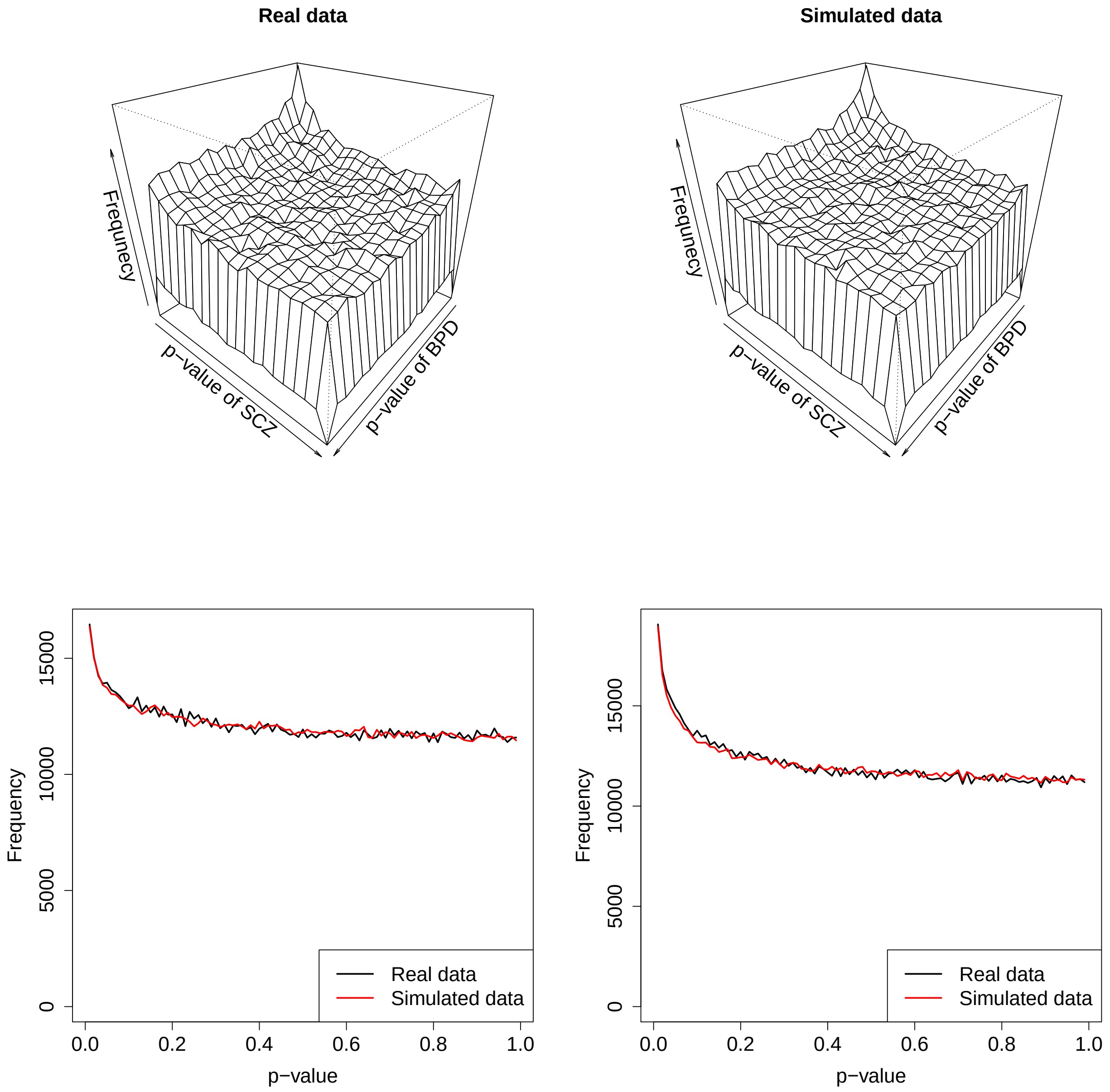

Figure S88: Upper left: The p-value histogram of real data (BPD and SCZ). Upper right: The histogram of p-values simulated from fitted GPA model. Lower left: Comparison between the p-values of BPD and the simulated p-values. Lower right: Comparison between the p-values of SCZ and the simulated p-values.

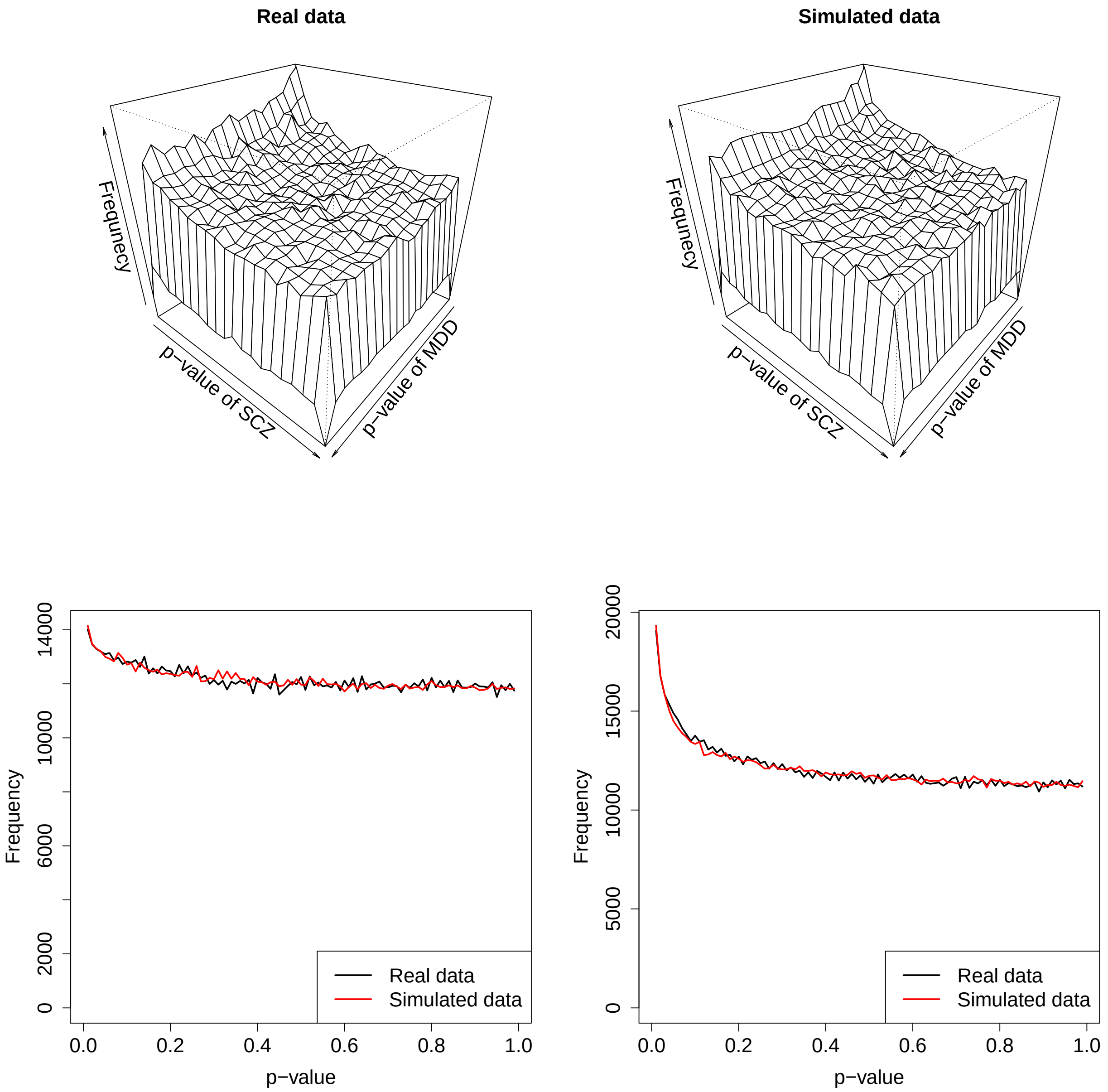

Figure S89: Upper left: The p-value histogram of real data (MDD and SCZ). Upper right: The histogram of p-values simulated from fitted GPA model. Lower left: Comparison between the p-values of MDD and the simulated p-values. Lower right: Comparison between the p-values of SCZ and the simulated p-values.

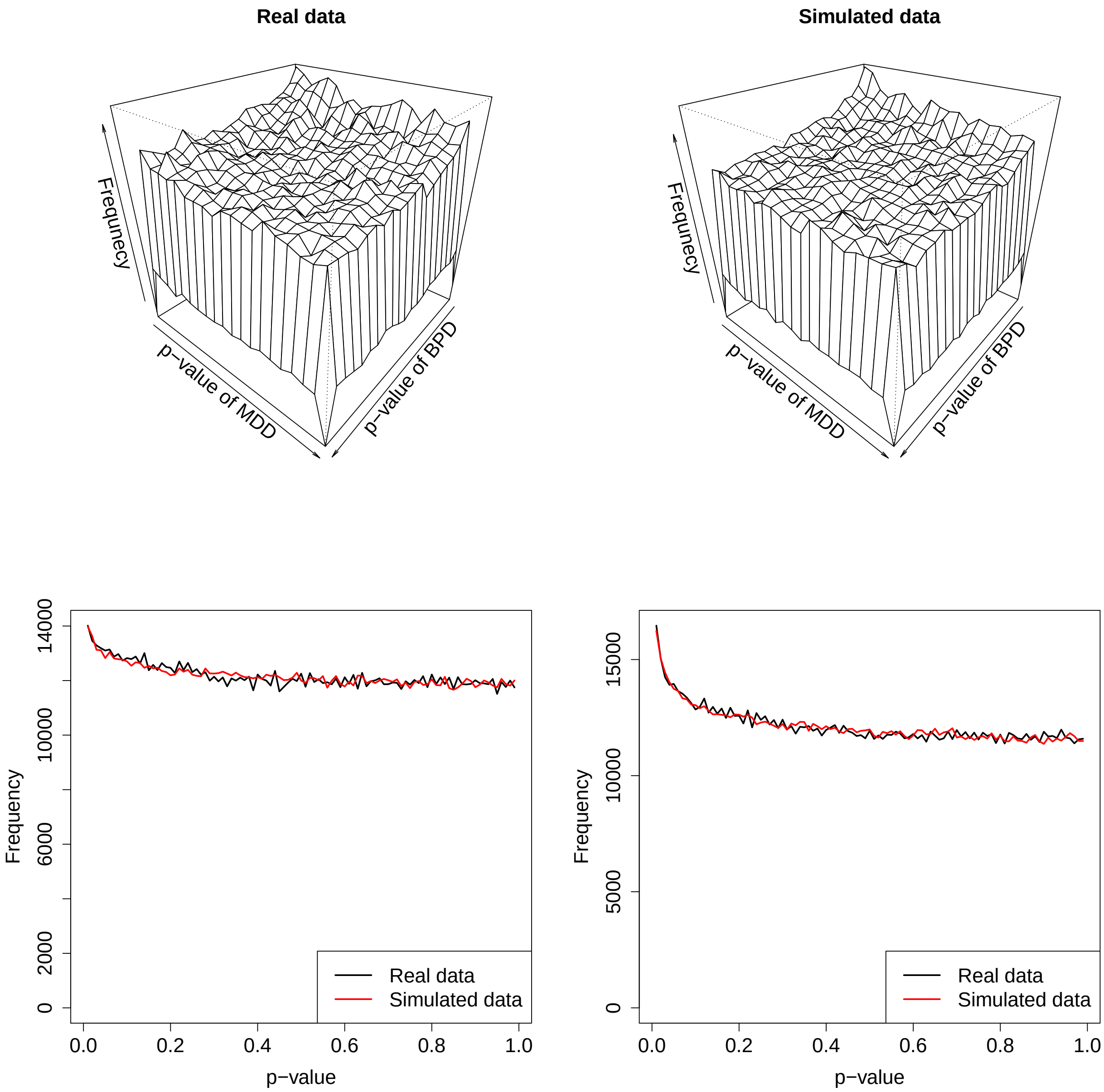

Figure S90: Upper left: The p-value histogram of real data (MDD and BPD). Upper right: The histogram of p-values simulated from fitted GPA model. Lower left: Comparison between the p-values of MDD and the simulated p-values. Lower right: Comparison between the p-values of BPD and the simulated p-values.

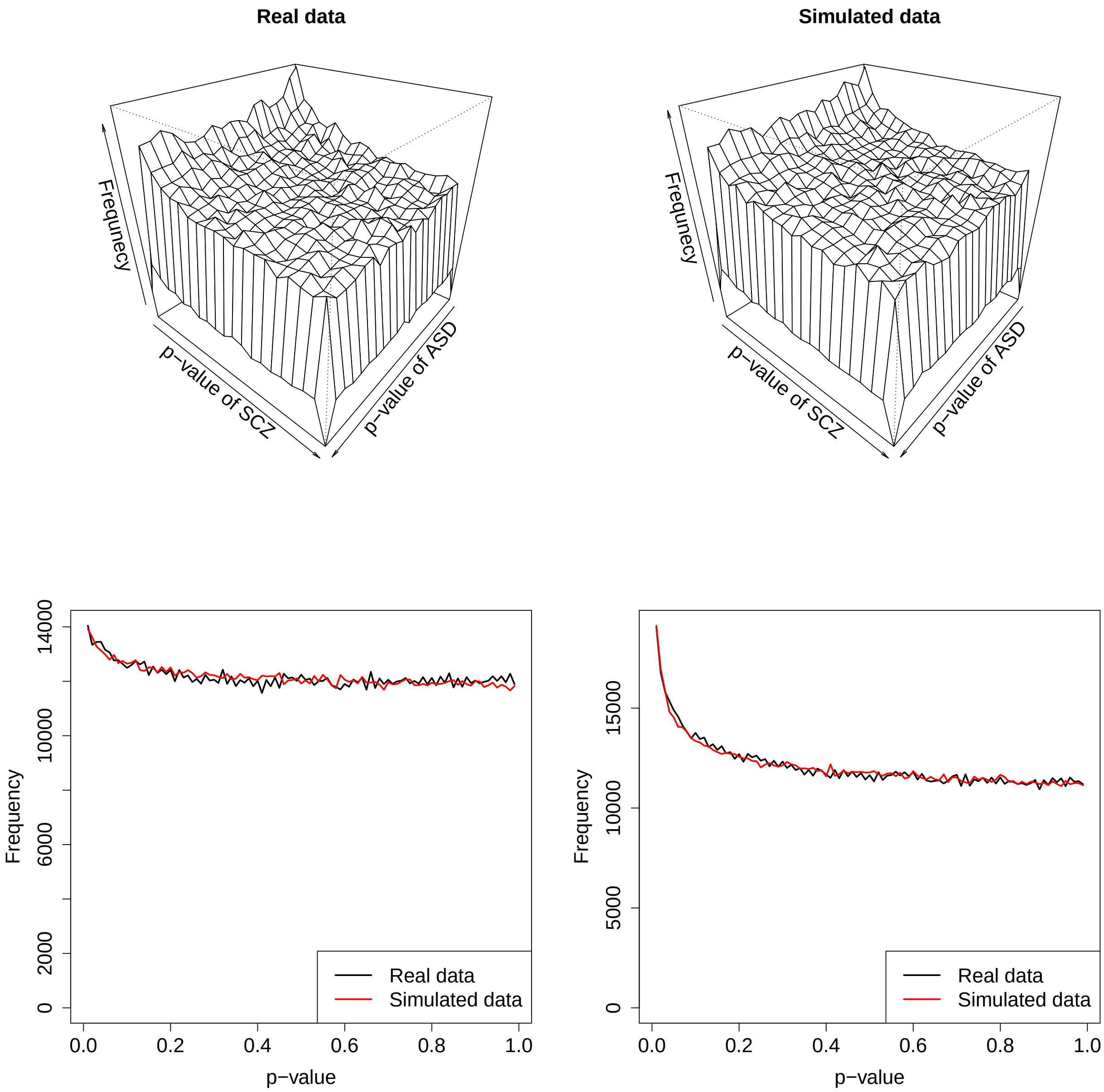

Figure S91: Upper left: The p-value histogram of real data (ASD and SCZ). Upper right: The histogram of p-values simulated from fitted GPA model. Lower left: Comparison between the p-values of ASD and the simulated p-values. Lower right: Comparison between the p-values of SCZ and the simulated p-values.

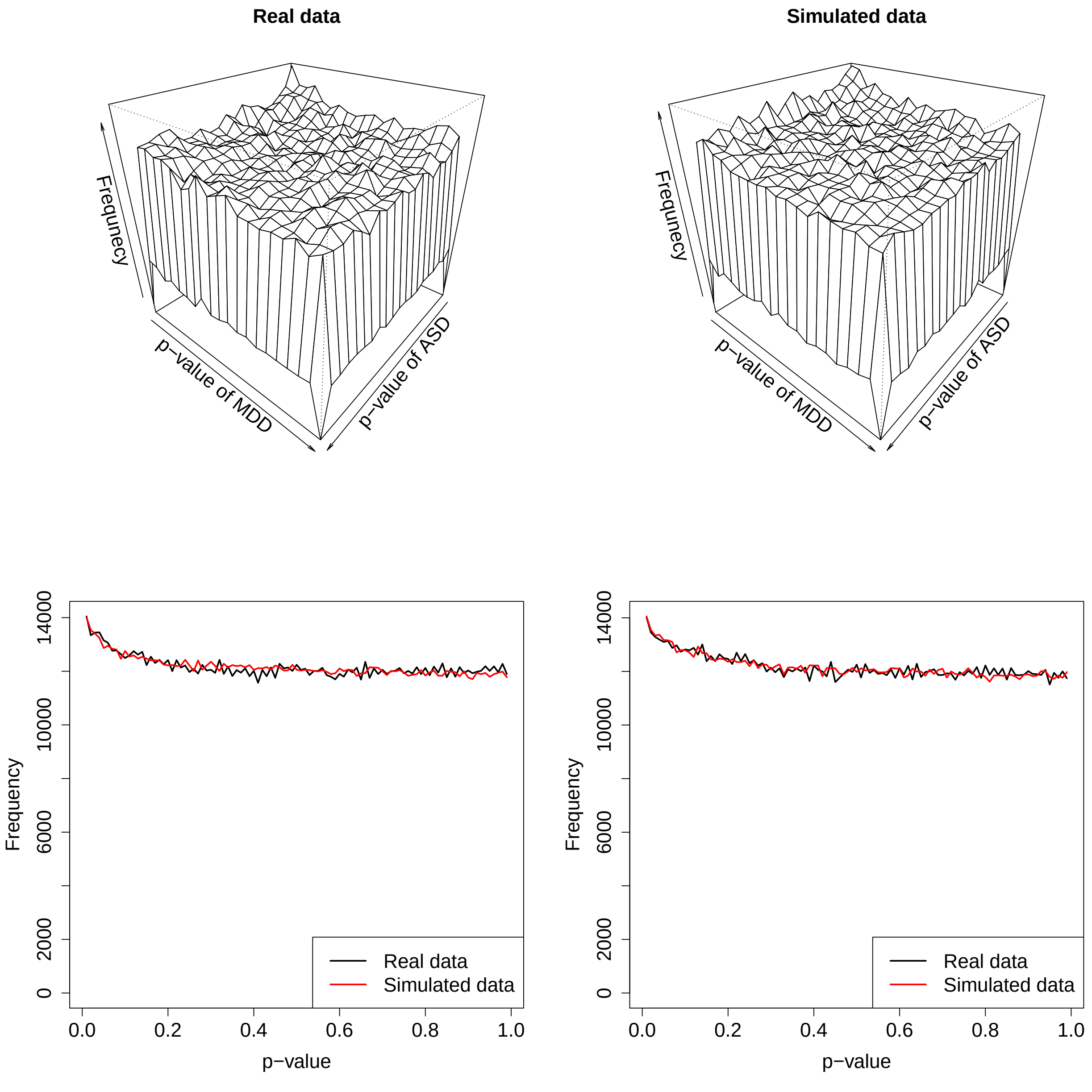

Figure S92: Upper left: The p-value histogram of real data (ASD and MDD). Upper right: The histogram of p-values simulated from fitted GPA model. Lower left: Comparison between the p-values of ASD and the simulated p-values. Lower right: Comparison between the p-values of MDD and the simulated p-values.

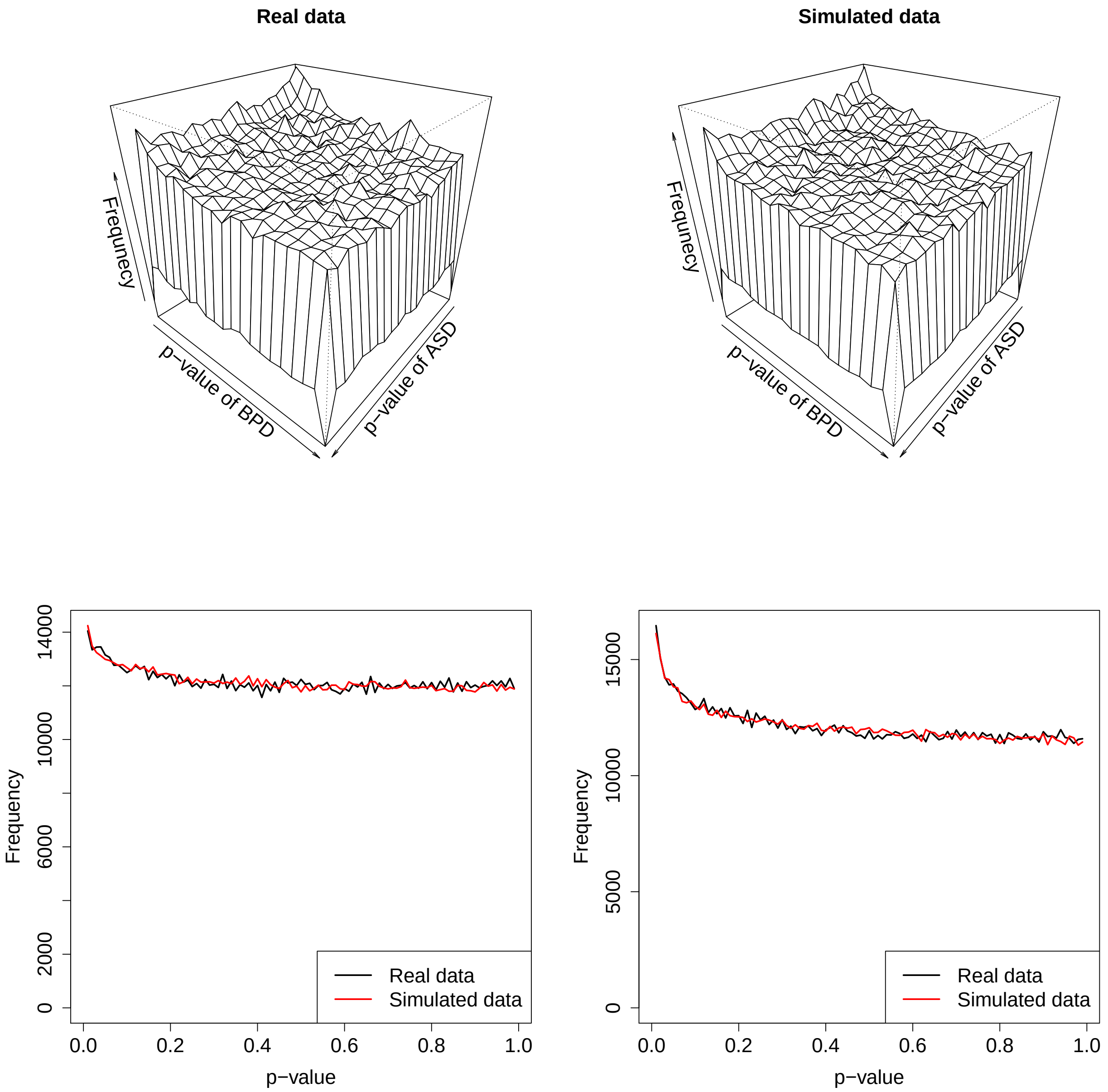

Figure S93: Upper left: The p-value histogram of real data (ASD and BPD). Upper right: The histogram of p-values simulated from fitted GPA model. Lower left: Comparison between the p-values of ASD and the simulated p-values. Lower right: Comparison between the p-values of BPD and the simulated p-values.

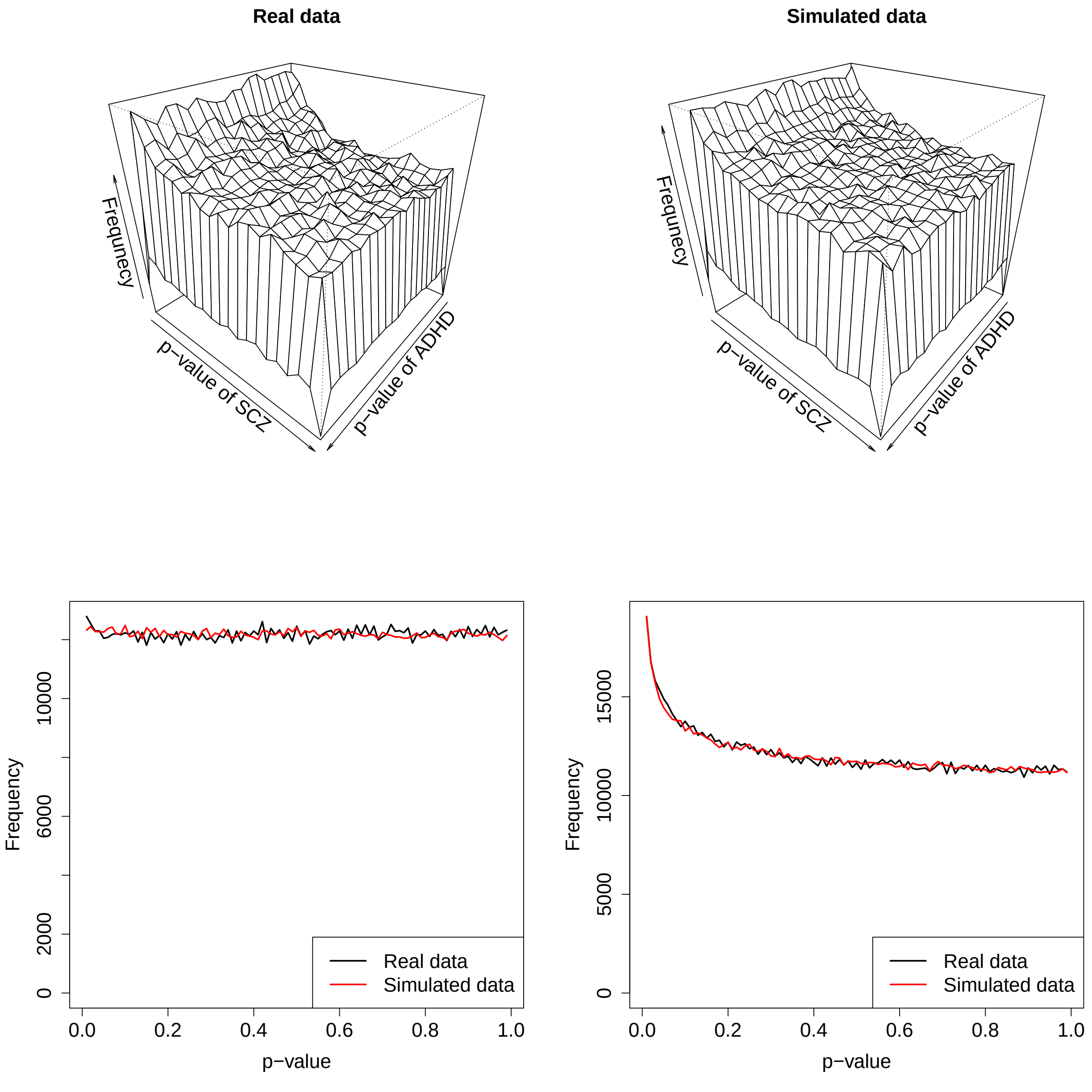

Figure S94: Upper left: The p-value histogram of real data (ADHD and SCZ). Upper right: The histogram of p-values simulated from fitted GPA model. Lower left: Comparison between the p-values of ADHD and the simulated p-values. Lower right: Comparison between the p-values of SCZ and the simulated p-values.

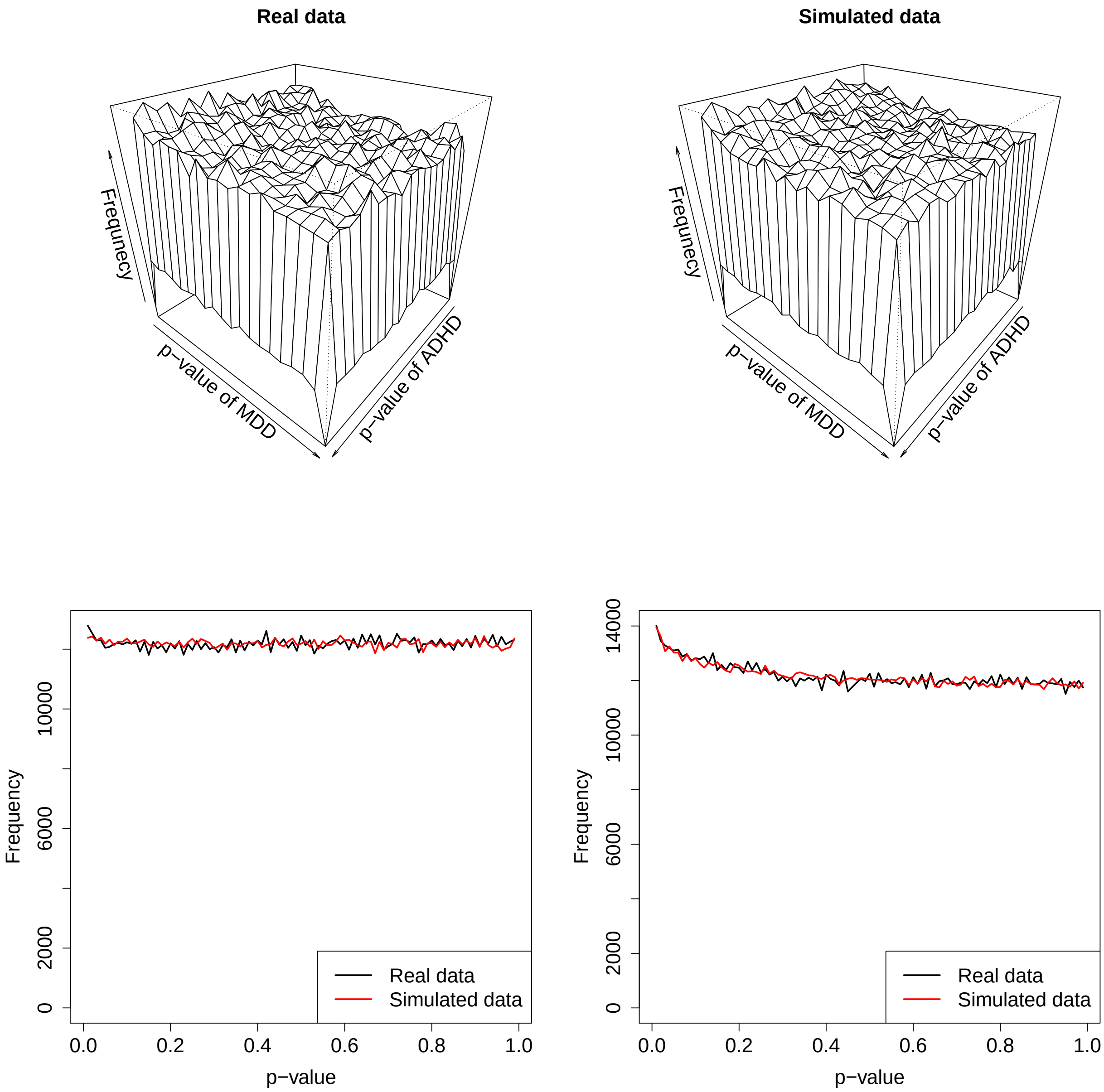

Figure S95: Upper left: The p-value histogram of real data (ADHD and MDD). Upper right: The histogram of p-values simulated from fitted GPA model. Lower left: Comparison between the p-values of ADHD and the simulated p-values. Lower right: Comparison between the p-values of MDD and the simulated p-values.

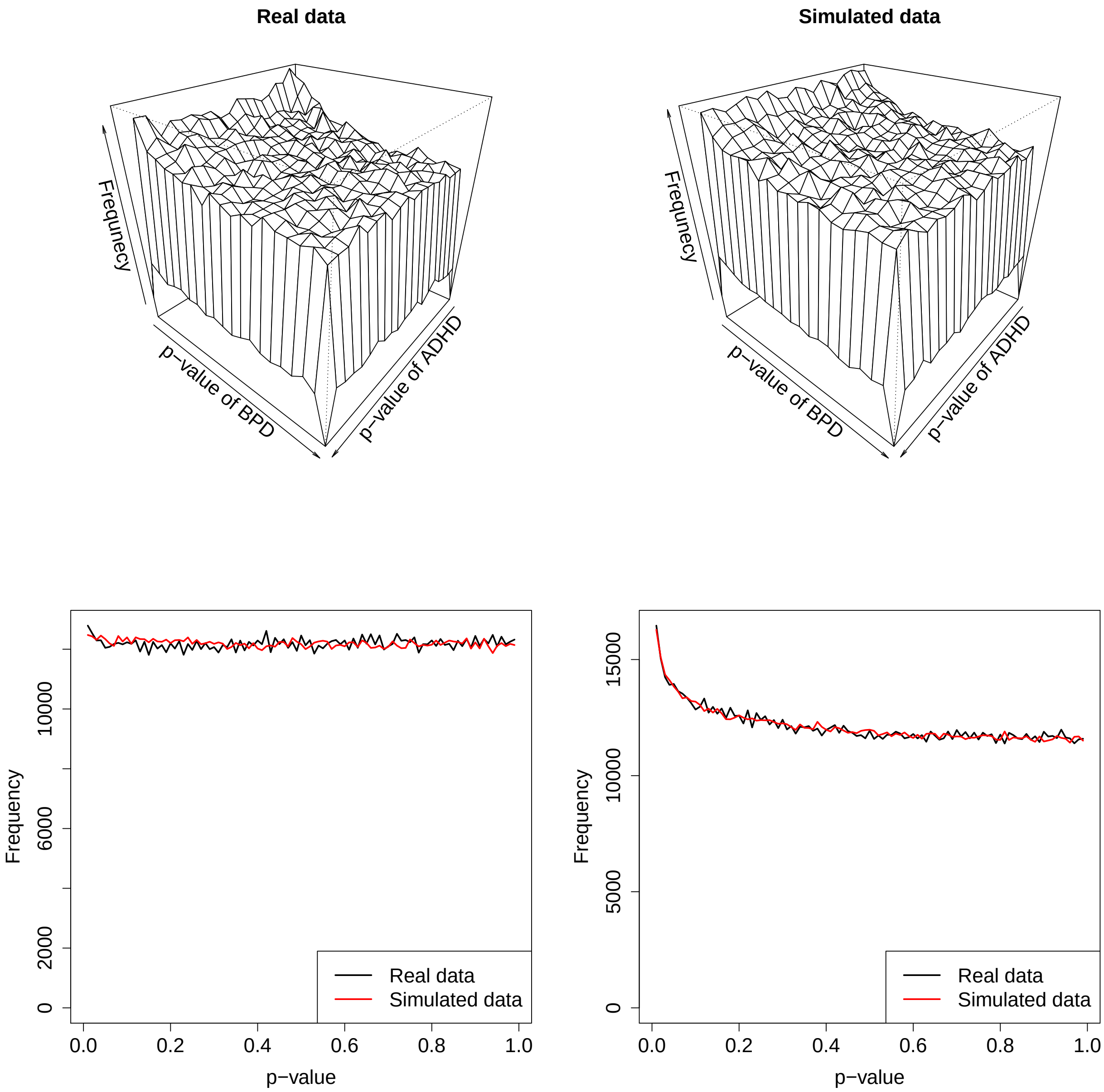

Figure S96: Upper left: The p-value histogram of real data (ADHD and BPD). Upper right: The histogram of p-values simulated from fitted GPA model. Lower left: Comparison between the p-values of ADHD and the simulated p-values. Lower right: Comparison between the p-values of BPD and the simulated p-values.

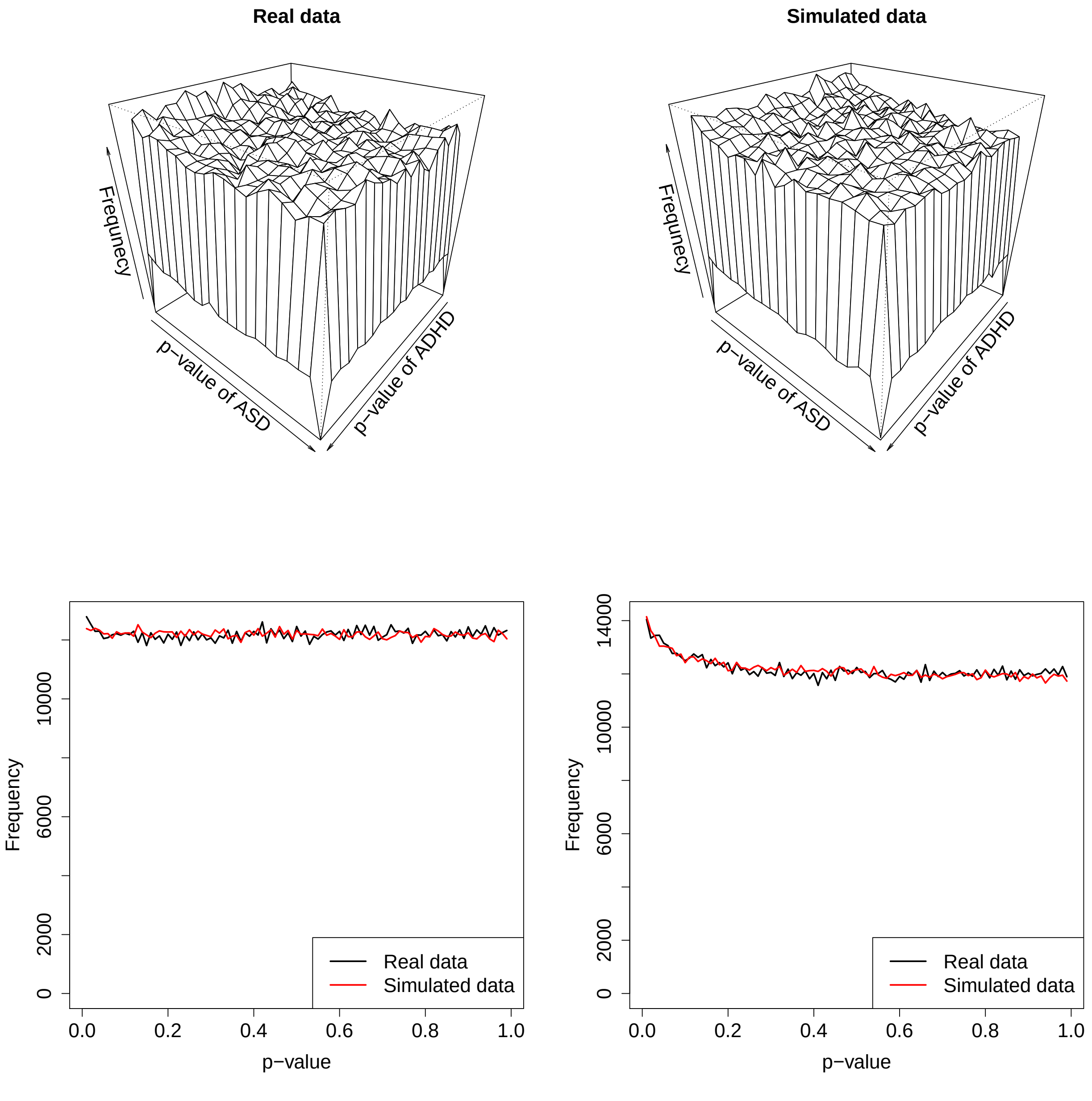

Figure S97: Upper left: The p-value histogram of real data (ADHD and ASD). Upper right: The histogram of p-values simulated from fitted GPA model. Lower left: Comparison between the p-values of ADHD and the simulated p-values. Lower right: Comparison between the p-values of ASD and the simulated p-values.

References

[1] Ole A Andreassen, Srdjan Djurovic, Wesley K Thompson, Andrew J Schork, Kenneth S Kendler, Michael C O'Donovan, and et al. Improved detection of common variants associated with schizophrenia by leveraging pleiotropy with cardiovascular-disease risk factors. *The American Journal of Human Genetics*, 92(2):97–109, 2013.

[2] Geoffrey McLachlan and Thriyambakam Krishnan. *The EM algorithm and extensions*. John Wiley & Sons, 2008.

[3] Michael Newton, Amine Noueiry, Deepayan Sarkar, and Paul Ahlquist. Detecting differential gene expression with a semiparametric hierarchical mixture method. *Biostatistics*, 5(2):155–176, 2004.

[4] Schizophrenia Working Group of the Psychiatric Genomics Consortium et al. Biological insights from 108 schizophrenia-associated genetic loci. *Nature*, 511(7510):421–427, 2014.

[5] Jun Shao. *Mathematical statistics*. Springer, 2nd edition, 2003.
